# Annulated carbocyclic gallylene and bis-gallylene with two-coordinated Ga(i) atoms†

**DOI:** 10.1039/d4sc06782g

**Published:** 2024-11-14

**Authors:** Arne Merschel, Shkelqim Heda, Yury V. Vishnevskiy, Beate Neumann, Hans-Georg Stammler, Rajendra S. Ghadwal

**Affiliations:** a Molecular Inorganic Chemistry and Catalysis, Inorganic and Structural Chemistry, Center for Molecular Materials, Faculty of Chemistry, Universität Bielefeld Universitätsstrasse 25 D-33615 Bielefeld Germany rghadwal@uni-bielefeld.de http://www.ghadwalgroup.de

## Abstract

The first carbocyclic gallylene [(ADC)_2_Ga(GaI_2_)] and bis-gallylene [(ADC)Ga]_2_ (ADC = PhC{N(Dipp)C}_2_; Dipp = 2,6-iPr_2_C_6_H_3_) featuring a central C_4_Ga_2_ ring annulated between two 1,3-imidazole rings are prepared by KC_8_ reductions of [(ADC)GaI_2_]_2_. Treatment of [(ADC)Ga]_2_ with Fe_2_(CO)_9_ affords complex [(ADC)GaFe(CO)_4_]_2_ in which each Ga(i) atom serves as a two-electron donor. [(ADC)Ga]_2_ activates white phosphorus (P_4_) and the C_sp^2^_–F bond of aryl fluorides (ArF) to yield compounds [(ADC)Ga(P_4_)]_2_ and *cis*-/*trans*-[(ADC)GaF(Ar)]_2_, respectively. [(ADC)Ga]_2_ undergoes oxidation with (Me_2_S)AuCl to give [(ADC)GaCl_2_]_2_, while with PhN=NPh it forms [1 + 4]-cycloaddition product [(ADC)GaN(Ph)N=C_6_H_5_]_2_ by the dearomatization of one of the phenyl rings.

## Abstract 2

The first carbocyclic gallylene [(ADC)_2_Ga(GaI_2_)] and bis-gallylene [(ADC)Ga]_2_ (ADC = PhC{N(Dipp)C}_2_; Dipp = 2,6-iPr_2_C_6_H_3_) featuring a central C_4_Ga_2_ ring annulated between two 1,3-imidazole rings are prepared by KC_8_ reductions of [(ADC)GaI_2_]_2_.

## Graphical abstract

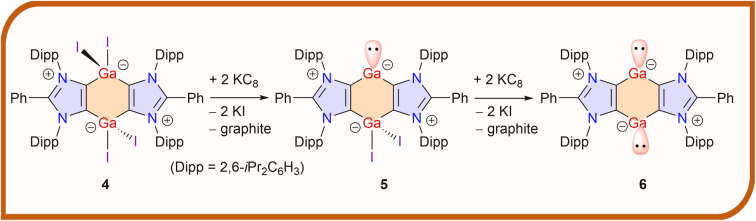

## Introduction

**Fig. 1:**
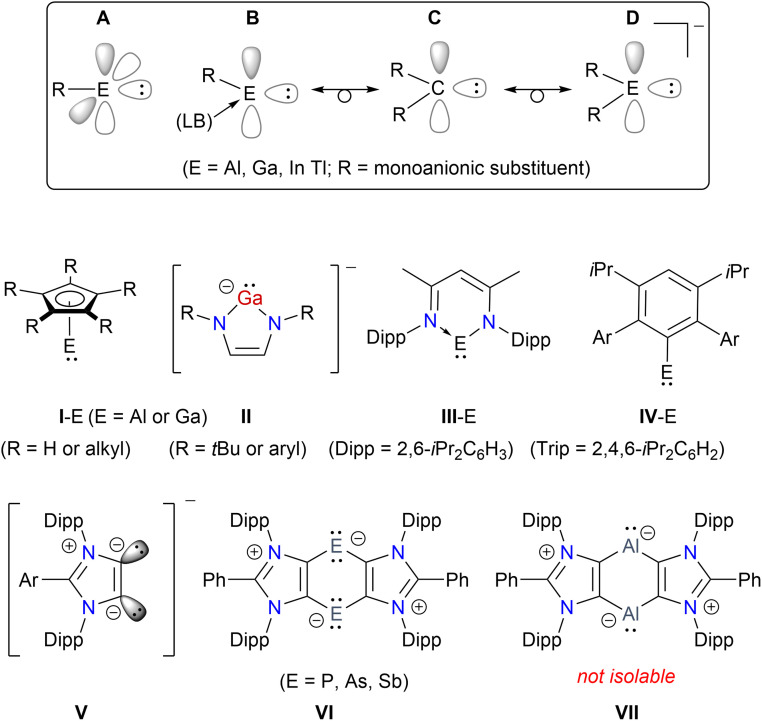
Schematic illustration of Group 13 metallylenes (A), Lewis base (LB) stabilized metallylenes (B), singlet carbenes (C), and anionic species (D). Representative examples E(I) compounds (I-E–IV-E). Anionic dicarbene (ADC) V derived Group 15 heterocycles VI and a related transient Al(i) compound VII.

**Scheme 1:**
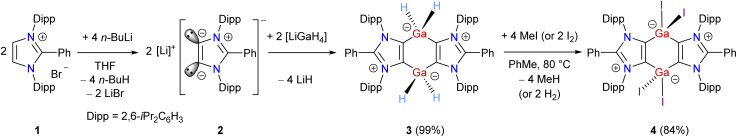
Synthesis of Ga(iii) hydride 3 and iodide 4.

**Scheme 2:**
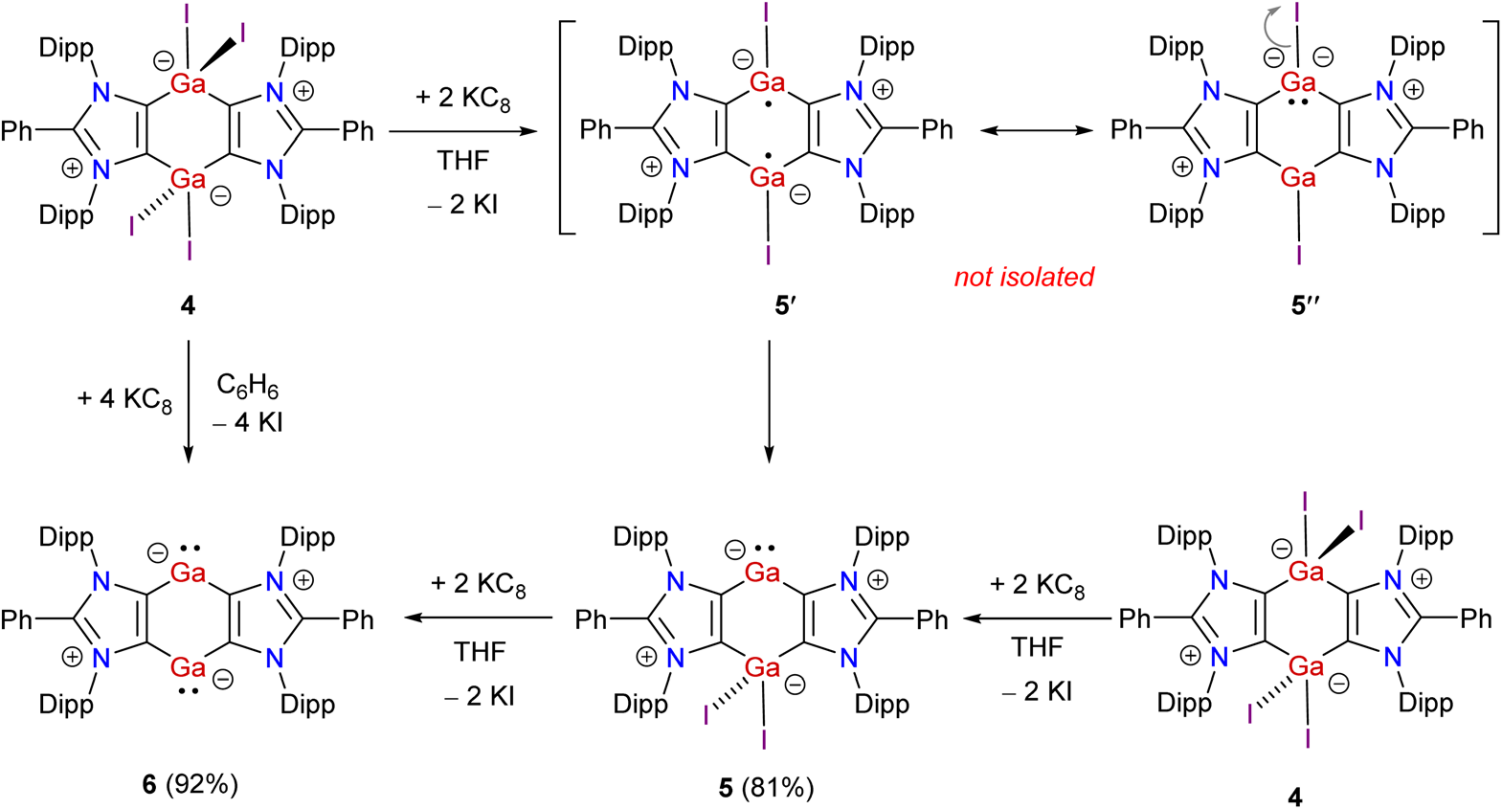
Synthesis of mixed-valent Ga(i/iii) compound 5 and Ga(i) compound 6.

The isolation of first crystalline N-heterocyclic carbene (NHC) by Arduengo^1^ prompted the search for stable carbene analogues of heavier Group 13 and 14 elements, *i.e.* metallylenes.^2^ Like carbenes, metallylenes are in general highly reactive species. The first Al(i) and Ga(i) compounds were reported as tetrameric species (I-E)_4_ (ref. 3) and [GaC(SiMe_3_)_3_]_4_ (ref. 4) in the solid state (Fig. 1). In 1999, Schmidbaur reported the first anionic Ga(i) compound II (R = *t*Bu).^5^ Subsequently, serval other anionic as well as neutral compounds with a two-coordinated Ga(i) or Al(i) atom were reported.^2,6^ In 2000, Roesky and Power independently reported the first aluminylene^7^ and gallylene^8^ compounds (III-E), respectively, based on a bulky β-diketiminate ligand. Over the past years, III-E have been extensively investigated to access a variety of Al/Ga-compounds with intriguing structures and properties.^6*a*^ The fascinating chemistry of these species prompted further interests in the isolation of new thermally stable Group 13 metallylenes.^9^ Among mono-coordinated metallylenes, Power *et al.* reported the first Ga(i) species Ar(Me_3_Si)NGa (Ar = 2,6-Mes_2_C_6_H_3_; Mes = 2,4,6-Me_3_C_6_H_2_) in 2006 (ref. 10) and the first Al(i) compound IV-E in 2020.^11^ Subsequently, the research groups of Liu,^12^ Hinz,^13^ and Tan^14^ reported monocoordinated Group 13 metallylene compounds using a bulky carbozole ligand. Very recently, Kretschmer and colleagues isolated a mono-coordinated Ga(i) compound.^15^ Like singlet carbenes, Group 13 metallylenes are promising ligands in organometallic chemistry.^12,16^ In general, most of the known examples of neutral as well as anionic metallylene species^9*b*,17^ are based on chelating N-donor ligands.^2,6^ The use of singlet carbenes for the stabilization of borylene species has been shown,^18^ however, related heavier metallylenes remained rather scarce.^19^ Like other main-group homonuclear heavier alkenes (*i.e.* for example the dimers of metallylenes),^20^ digallenes may also be regarded as dimers of gallylenes.^10,21^ Among three coordinated Ga(i) compounds, Lewis base-stabilized neutral^22^ as well as dicationic^23^ digallene compounds are known. We have shown the suitability of 1,3-imidazole-based anionic dicarbenes (ADCs) V in accessing a variety of low-valent main-group heterocycles.^24^ Compounds VI featuring formally P(i),^25^ As(i)^26^ or Sb(i)^27^ atoms are accessible as crystalline solids. Attempts to isolate the aluminylene species VII were unfortunately unsuccessful as it underwent C–H bond activation to yield an annulated Al(iii) compound in 76% yield.^28^ Herein, we report the first carbocyclic gallylene as well as bis-gallylene compounds (Schemes 1 and 2) as crystalline solids and showcase the reactivity of the bis-gallylene towards transition metal, white phosphorus, organofluorine, and azobenzene substrates.

## Results and discussion

The starting Ga(iii) hydride [(ADC)GaH_2_]_2_ (3) (ADC = PhC{N(Dipp)C}_2_, Dipp = 2,6-iPr_2_C_6_H_3_) was prepared by reacting freshly prepared LiGaH_4_ and Li(ADC) (2)^29^ as a colorless crystalline solid in 99% yield (Scheme 1). Compound 1 was synthesized by the direct C2-arylation of the corresponding NHC under nickel catalysis.^30^ Compound 3 is stable under an inert gas (N_2_ or Ar) atmosphere but slowly decomposes when exposed to air. The ^1^H and ^13^C{^1^H} NMR spectra of 3 exhibit well-resolved signals, which are fully consistent with the related aluminium species [(ADC)AlH_2_].^31^ The ^1^H NMR spectrum of 3 shows a broad signal at 4.16 ppm for the Ga*H*_2_ moieties that is comparable with those of NHC-stabilized Ga(iii) hydrides.^32^ The FT-IR spectrum of 3 displays two characteristic bands at 1800 and 1830 cm^−1^ for the Ga–H stretching vibrations.^32,33^ Treatment of 3 with methyl iodide (or iodine) at 80 °C affords the Ga(iii) iodide 4 as a white solid. Compound 4 is insoluble in benzene and toluene but sparingly dissolves in chloroform. The ^1^H and ^13^C{^1^H} NMR spectra of 4 show broad signals for the isopropyl groups, which is in line with an easily polarizable nature of iodides.^28^ The ^13^C{^1^H} NMR signal for the gallium bound carbon atoms of 4 (164.0 ppm) is slightly downfield shifted compared to that of 3 (158.3 ppm).

**Fig. 2:**
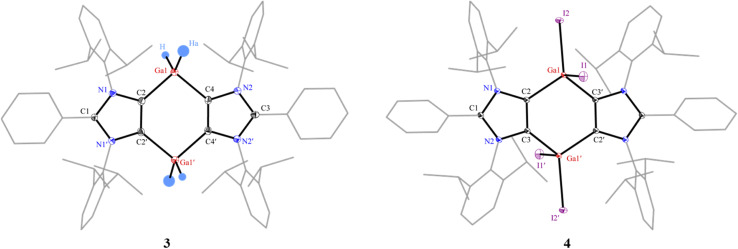
Solid-state molecular structures of 3 and 4. Hydrogen atoms (except on GaH_2_ for 3) and two benzene molecules (for 3) are omitted for clarity. Aryl groups are depicted as wireframes. Thermal displacement ellipsoids at 50%. Selected bond lengths (Å) and angles (°) for 3: C2–Ga1 2.016(3), C4–Ga1 2.015(3), C2′–C2 1.377(6), C2–Ga1–C4 101.2(1), symmetry code: 2 − *x*, 1 − *y*, *z*; and for 4: C2–Ga1 1.993(1), C3′–Ga1 1.993(1), Ga1–I1 2.6117(1), Ga1–I2 2.5230(1), C2–C3 1.378(1), I1–Ga1–I2 107.47(1), C2–Ga1–I1 100.14(2), C2–Ga1–I2 117.85(2), C2–Ga1–C3′ 107.36(3), symmetry code: 1 − *x*, 1 − *y*, 1 − *z*.

The solid-state molecular structures of 3 and 4 (Fig. 2) show the expected atom connectivity.^34^ The four-fold coordinated Ga(iii) atoms in the C_4_Ga_2_ ring of 3 and 4 have a distorted tetrahedral coordination geometry. The C2–Ga1 (2.016(3) Å) and C4–Ga1 (2.015(3) Å) bond lengths of 3 are slightly smaller than those of NHC–Ga(iii) hydrides (2.071(5) Å).^32^ This may be attributed to the stronger σ-donor strength of mesoionic carbenes (iMICs) than NHCs.^35^ The gallium bound hydrogen atoms of 3 were refined isotropically. The C2–Ga1–H (108.1(2)°) and C2–Ga1–Ha (113.7(2)°) bond angles of 3 are distinct. Like the Ga–H/Ha bond lengths in 3 (1.492(3)/1.574(3) Å), the Ga1–I1 (2.6117(1) Å) and Ga1–I2 (2.5230(1) Å) bond lengths of 4 are dissimilar (see below the NBO (Natural Bond Orbital) analysis). The I1 atom of 4 is situated out of the C_4_Ga_2_-ring plane, resulting the C3–C2–Ga1–I1 torsion angle of 104.1(1)°. The C2, Ga1, C3′, and I2 atoms are positioned in a semi-trigonal plane with the C3–C2–Ga1–I1 torsion angle of 168.1(1)°.

Having the desired Ga(iii) compound 4 in hand, we prompted to perform its reduction. Treatment of 4 with 2 equivalents of KC_8_ in THF affords the mixed-valent Ga(i/iii) compound 5 as a brown solid in 81% yield (Scheme 2). The exact mechanism of the formation of 5 is currently not known. Direct reduction of one of the Ga(iii) atoms of 4 by two equivalents of KC_8_ to give 5 is likely. The reduction of 4 to give the putative Ga(ii) species 5′, which may also be viewed as a zwitterionic species 5′′, cannot be ruled out. Finally, the disproportionation of 5′ (*i.e.* formally an intramolecular iodide ion transfer in 5′′) into 5 is calculated to be thermodynamically favored by 17.6 kcal mol^−1^ (see below). Reaction of 4 with 4 equivalents of KC_8_ in benzene leads to the clean formation of bis-gallylene 6 as a red-brown solid in 92% yield. 6 can also be prepared by reacting 5 with KC_8_. 5 and 6 are crystalline solids, soluble in common organic solvents (THF, toluene, benzene), and stable under an inert atmosphere (of N_2_ or Ar) for several weeks. The ^1^H NMR spectrum of mixed-valent Ga(i/iii) compound 5 exhibits four doublets and two septets for the isopropyl groups, suggesting unsymmetrical coordination settings at the Ga atoms. In contrast, the ^1^H NMR spectrum of bis-gallylene 6 shows two doublets and one septet for the isopropyl groups, which are consistent with the higher symmetry of 6 than 5. A similar ^1^H NMR signal pattern is also observed for isostructural compounds featuring Group 14 (ref. 36) or 15 (ref. 25–27) elements in a formally +1 oxidation state. The ^13^C{^1^H} NMR spectrum of 5 shows two resonances at 163.6 (*C*GaI_2_) and 176.4 (*C*Ga) ppm for the *C*_4_Ga_2_ moiety. The ^13^C{^1^H} NMR spectrum of 6 exhibits one signal at 173.6 ppm for the *C*_4_Ga_2_ unit.

**Fig. 3:**
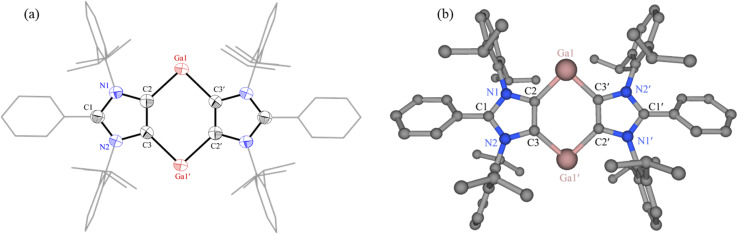
(a) Solid-state molecular structure of 6. Only one of two crystallographic independent molecules in the asymmetric unit is shown. Hydrogen atoms, minor disordered parts and solvent molecules were omitted for clarity. Aryl groups are depicted as wireframes. Thermal displacement ellipsoids at 50%. Selected bond lengths (Å) and angles (°): C2–Ga1 2.092(4), C3′–Ga1 2.107(4), C2–C3 1.388(5), C2–Ga1–C3′ 92.6(1), C3–C2–Ga1 132.6(3), symmetry code: 1 − *x*, 1 − *y*, 1 − *z*. (b) Optimized structure of 6 (r^2^SCAN-3c). Selected equilibrium parameters (in Å/°): Ga1–C2 2.128, Ga1–C3′ 2.113, C2–C3 1.393, C2–Ga1–C3′ 91.8.

The solid-state molecular structure of 6 features a planar C_4_Ga_2_ core embedded between two 1,3-imidazole moieties (Fig. 3a). Compound 6 with two-coordinated Ga(i) atoms may be regarded as a base-stabilized bis-gallylene. Due to the phase transition below 220 K, the reflection data for 6 were collected at 220 K, giving rise to large thermal displacement ellipsoids. The C2–Ga1 (2.092(4) Å) and C3′–Ga1 (2.107(4) Å) bond lengths of 6 are marginally larger than those of Ga(iii) compounds 3 (2.016(3) Å) and 4 (1.993(1) Å). The C2–Ga1–C3′ bond angle of 6 (92.6(1)°) is smaller than those of 3 (101.2(1)°) and 4 (107.4(1)°).

To obtain a further insight into the electronic structures of 5 and 6, we performed quantum chemical calculations at the r^2^SCAN-3c level of theory. The optimized molecular structure of 6 is in good agreement with that of sc-XRD structure (Fig. 3). Calculations reveal closed-shell singlet (CS) ground state for 5 (Fig. S60†) and 6. The triplet (T) solution for the putative intermediate 5′ (Fig. S61†) is 0.26 kcal mol^−1^ more stable than the CS solution. The conversion 5′ → 5 is calculated to be thermodynamically favored by 17.6 kcal mol^−1^. The NBO charges and WBIs (Wiberg Bond Indices) calculated at the PBE0/def2-TZVPP level of theory for 4, 5, and 6 (Table S7†) are consistent with their solid-state structures. With the WBIs of 0.45–0.54 and NBO charges of *ca.* −0.32*e* on C atoms, the Ga–C bonds of 4, 5, and 6 are essentially polar covalent bonds that are polarized towards the C atoms. The NBO charge(s) on the Ga atom(s) of 4 (1.02), 5 (1.02/0.52), and 6 (0.48) is consistent with its formal (I/III) oxidation state.

**Fig. 4:**
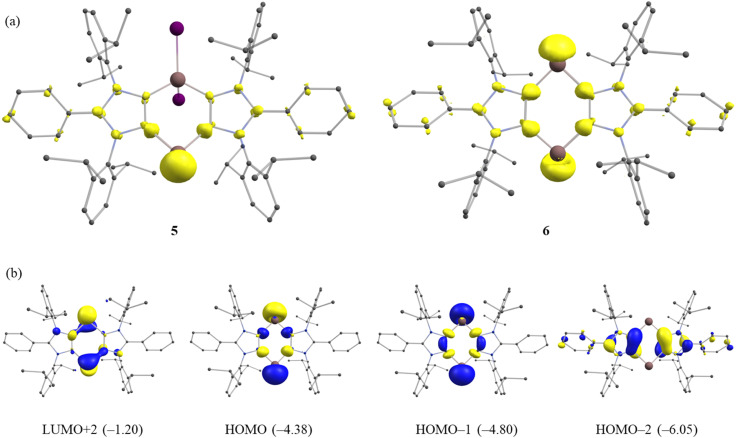
(a) FOD plots (isosurfaces 0.005) of compounds 5 and 6. Hydrogen atoms are omitted for clarity. (b) Selected molecular orbitals (0.05) with respective energies (in eV) of 6 according to RKS-PBE0/def2-TZVPP calculations.

We also performed FOD (fractional occupation number weighed density) calculations^37^ at the PBE0/def2-TZVPP level of theory to analyze the electron correlation in 5 and 6. The FOD analyses provide reliable information on the localization of ‘hot’ (strongly correlated and chemical active) electrons of the molecule.^37^ The FOD calculations (PBE0, *T* = 10 000 K) reveal a moderate level of electron correlation in 5 (*N*_FOD_ = 2.50 *e*) and 6 (*N*_FOD_ = 2.85 *e*) (Fig. 4a). The *N*_FOD_ of 6 is smaller than that of the transient bis-aluminylene VII (*N*_FOD_ = 3.10 *e*).^28^

The HOMO and HOMO−1 of 6 (Fig. 4b) are essentially the electron lone-pairs on the Ga(i) atoms. The LUMO (Fig. S64†) of 6 is based on the ligand, while the LUMO+2 is mainly located on the Ga atoms. The HOMO–LUMO energy gap (Δ*E*_H–L_) for 6 amounts to 2.83 eV, which is larger than that of the corresponding fleeting Al(i) species VII (1.94 eV).^28^ In line with this, the HOMO (Fig. S63†) of mono-gallylene 5 is the lone-pair on Ga(i) atom, while the LUMO+2 is located mainly at the Ga(i) atom. The HOMO of 5 (−4.84 eV) is low-lying than that of 6 (−4.38 eV), suggesting a higher Lewis basicity of the latter. A rather smaller Δ*E*_H–L_ for 6 (2.83 eV) than 5 (3.18 eV) also implies a greater reactivity of 6. The UV-Vis spectrum of 5 and 6 each exhibits a main absorption band (*λ*_max_) at 300 and 312 nm, respectively (Fig. S48 and S49†). This may be assigned to HOMO−1 → LUMO (5) and HOMO−2 → LUMO (6) transition.

**Scheme 3:**
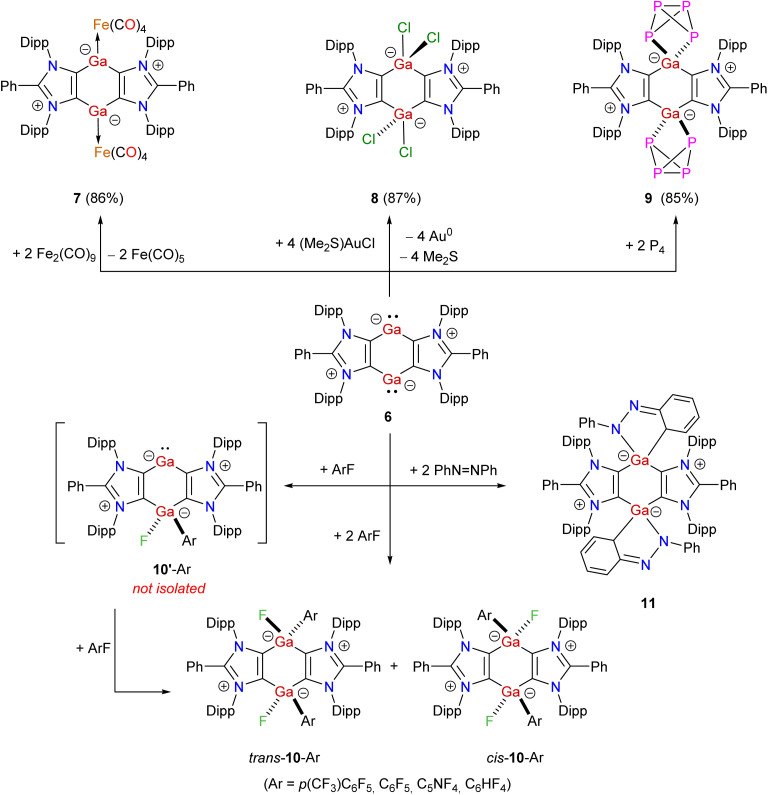
Room temperature reactivity studies of 6 towards different substrates to 7–11.

Treatment of 6 with Fe_2_(CO)_9_ yields the dinuclear Fe(0) complex 7 (Scheme 3). Compound 6 readily oxidizes with (Me_2_S)AuCl to yield Ga(iii) compound 8 as a colorless solid. Reaction of 6 with white phosphorus affords compound 9 as a brown solid. Prompted by the use of low-valent main-group compounds in the activation of C–F bonds,^38^ we performed reactions of 6 with different aryl fluorides (ArF) at room temperature that gave Ga(iii) compounds *cis*-/*trans*-10-Ar (Ar = *p*-(CF_3_)C_6_F_4_, C_6_F_5_, C_5_NF_4_, C_6_HF_4_) as off-white solids. The formation of *cis*-/*trans*-10-Ar isomers is likely due to the stepwise addition of Ar–F to 6*via* mixed-valent Ga(i)/Ga(iii) species 10′-Ar. Treatment of 6 with azobenzene at room temperature leads to the [1 + 4]-cycloaddition product 11 in which one of the phenyl rings of azobenzene has dearomatized. The ^1^H and ^13^C{^1^H} NMR spectra of 7–11 exhibit expected resonances for the ADC moieties. The ^13^C{^1^H} NMR spectrum of 7 features a signal at 216.6 ppm for the carbonyl carbon atoms of the Fe(CO)_4_ fragments.^39^ The ^1^H NMR spectrum of 8 compares well with that of the related aluminium chloride.^28^ The ^31^P{^1^H} NMR spectrum of 9 shows two triplets at 152.3 and −298.7 ppm (^1^*J*_P–P_ = 157 Hz), which are consistent with those of a related isostructural P_4_-activation product.^40^ The ^19^F{^1^H} NMR spectra of *cis*-/*trans*-10-Ar reveal expected signals for the aryl fluoride moieties as well as a signal for the Ga*F* group in the −205.6 to −207.8 ppm region.^38^

**Fig. 5:**
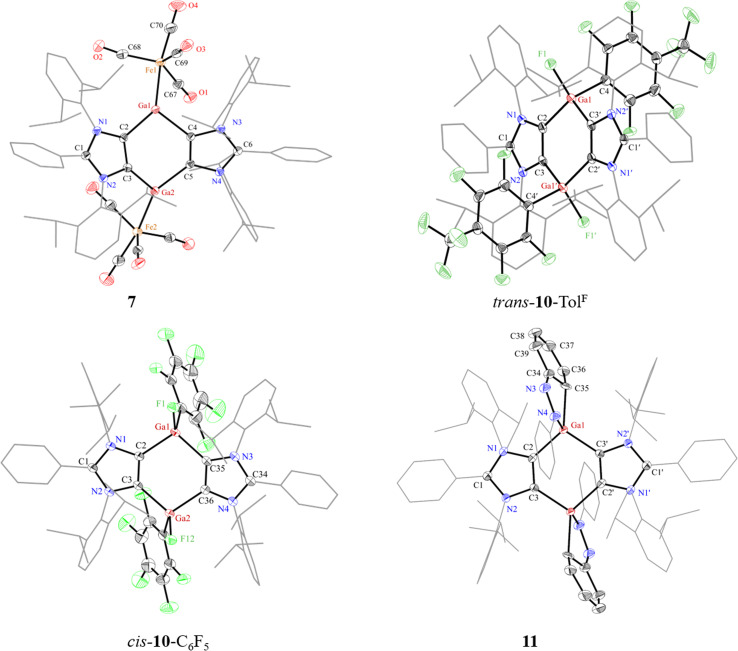
Solid-state molecular structures of 7, *trans*-10-Tol^F^, *cis*-10-C_6_F_5_ and 11. Hydrogen atoms, minor occupied atoms and solvent molecules were omitted for clarity. Aryl groups (except C_6_F_4_/C_6_F_5_ for *trans*-/*cis*-10-Ar and N3=C_6_H_5_ for 11) are depicted as wireframes. Thermal displacement ellipsoids at 50%. For *trans*-10-Tol^F^, only one of two crystallographic independent fragments in the asymmetric unit is shown. Selected bond lengths (Å) and angles (°) for 7: C2–Ga1 2.021(2), C4–Ga1 2.010(2), C3–Ga2 2.009(2), C5–Ga2 2.028(2), Ga1–Fe1 2.327(1), Ga2–Fe2 2.320(1), C2–Ga1–C4 99.9(1), C2–Ga1–Fe1 119.6(1), C4–Ga1–Fe1 139.7(1), C3–Ga2–C5 99.8(1), C3–Ga2–Fe2 139.2(1), C5–Ga2–Fe2 120.3(1); for *trans*-10-Tol^F^: C2–Ga1 1.997(3), C3′–Ga1 1.997(3), C4–Ga1 2.017(3), Ga1–F1 1.795(2), C2–C3 1.375(4), C2–Ga1–C3′ 106.5(1), symmetry code: 1 − *x*, 1 − *y*, 1 − *z*; for *cis*-10-C_6_F_5_: C2–Ga1 1.999(1), C35–Ga1 2.003(1), C3–Ga2 1.982(1), Ga1–F1 1.800(1), C2–C3 1.369(2), C2–Ga1–C35 103.8(1); for 11: C2–Ga1 2.009(2), C3′–Ga1 2.008(2), Ga1–C35 2.052(2), Ga1–N4 1.969(2), N3–N4 1.394(3), N3–C34 1.294(4), C34–C35 1.472(4), C2–Ga1–C3′ 103.8(1), N4–Ga1–C35 82.4(1).

The solid-state molecular structures of 7 (Fig. 5), 8 (Fig. S54†), *trans*-10-Tol^F^, *cis*-10-C_6_F_5_ (Fig. 5), *trans*-10-C_5_NF_4_ (Fig. S57†), and 11 (Fig. 5) show the expected atom connectivity.^41^ The structure of 7 revealed a minor impurity (∼3%) with two iodine atoms instead of one Fe(CO)_4_ group. This is likely due to the presence of a trace of 5 with 6 used to prepare 7, which was however not observed in the NMR spectroscopic analysis of the same sample. The sum of the angles at each of three-coordinated Ga atoms of 7 amounts to 359° that is consistent with a trigonal planar coordination environment. The Ga1–Fe1 (2.327(1) Å) and Ga2–Fe2 (2.320(1) Å) bond lengths of 7 are larger than that of [(Cp*Ga)Fe(CO)_4_] (2.273(1)Å).^39^ The four-coordinated Ga atoms of 8, 9, *cis*-/*trans*-10-Ar, and 11 show a distorted tetrahedral coordination environment. As expected for an oxidized product, the C2–Ga1 (1.997(3) Å) and C3′–Ga1 (1.997(3) Å) bond lengths of *trans*-10-Tol^F^ are smaller than that of 6 (Ga1–C2: 2.092(4) Å), while the Ga1–F1 (1.795(2) Å) bond lengths of *trans*-10-Tol^F^ agree well with other Ga(iii) fluorides.^38^ A similar trend was shown by *cis*-10-C_6_F_5_ and *trans*-10-C_5_NF_4_. In 11, the N3–N4 (1.394(3) Å) and C34–C35 (1.472(4) Å) bond lengths are consistent with a single bond,^42^ while N3–C34 (1.294(4) Å) bond length corresponds to a C=N double bond.^43^ These features show the dearomatization of one of the phenyl groups of azobenzene in 11.

## Conclusions

In conclusion, the first carbocyclic gallylene 5 and bis-gallylene 6 have been reported as crystalline solids. The formation of the mixed-valent Ga(i/iii) compound 5 may likely a result of the disproportionation of the transient Ga(ii) species 5′. In addition to the sc-XRD structures of 4 and 6, electronic structures of 4, 5, 5′, and 6 have been investigated by quantum chemical calculations. Preliminary reactivity studies of 6 have been presented with Fe_2_(CO)_9_ (coordination chemistry), Au(i) chloride (oxidation), white phosphorus (small molecule activation), aryl fluoride (C–F activation), and azobenzene (dearomative cycloaddition) to afford compounds 7, 8, 9, *cis*-/*trans*-10-Ar, and 11, respectively. Further reactivity studies and use of 5 and 6 as ligands as well as substrates for new gallium compounds, in particular aromatic systems and radicals, are expected to add new facets in low-valent gallium chemistry.

## Data availability

Experimental details, the plots of NMR, FT-IR, and UV-Vis spectra as well as the detail of X-ray crystallography and theoretical studies of the reported compounds are given in the ESI.† The assigned CCDC identification numbers and the compounds in the study are as follows: CCDC 2334945 (3), 2334948 (4), 2334949 (6), 2334950 (7), 2334951 (*trans*-10-Tol^F^), 2334952 (8), 2379059 (*cis*-10-C_6_F_5_), 2379060 (*trans*-10-C_5_NF_4_), and 2334953 (11).

## Author contributions

AM and SH: experimental investigation, data collection and analysis, writing. YVV: calculations, data collection and analysis, writing. BN and HGS: sc-XRD, data collection and analysis, writing. RSG: conceptualization, investigation, data analysis, writing, editing, supervision. All authors approved the manuscript.

## Conflicts of interest

The authors declare no conflict of interest.

## Supplementary Material

SC-OLF-D4SC06782G-s001

SC-OLF-D4SC06782G-s002
