# Supplementary material for: Annulated carbocyclic gallylene and bis-gallylene with two-coordinated Ga(i) atoms
Source: Chem Sci. 2024 Nov 14;16(5):2222–30. doi: 10.1039/d4sc06782g (PMC11706234; doi:10.1039/d4sc06782g)
Supplement: SC-016-D4SC06782G-s001 [file SC-016-D4SC06782G-s001.pdf]

Supplementary Information (SI) for Chemical Science.
This journal is © The Royal Society of Chemistry 2025

*Supporting Information*

*for*

# Annulated Carbocyclic Gallylene and Bis-Gallylene with Two-Coordinated Ga(I) Atoms

Arne Merschel, Shkelqim Heda, Yury V. Vishnevskiy, Beate Neumann, Hans-Georg Stammler, and Rajendra S. Ghadwal*

*Molecular Inorganic Chemistry and Catalysis, Inorganic and Structural Chemistry, Center for Molecular Materials, Faculty of Chemistry, Universität Bielefeld, Universitätsstrasse 25, D-33615, Bielefeld, Germany.*

E-mail: rghadwal@uni-bielefeld.de; Homepage: www.ghadwalgroup.de

# Table of Contents


Materials and Methods ........ S1

Plots of NMR Spectra ........ S10

Plots of FT-IR Spectra ........ S34

Plots of UV-Vis Spectra ........ S35

Crystallographic Details ........ S36

Computational Details ........ S44

Cartesian Coordinates ........ S55

References ........ S76

# Materials and Methods

All syntheses and manipulations were carried out under an inert gas atmosphere (Ar or $N_2$) using standard Schlenk techniques or a glove box (MBraun LABMasterPro). Solvents were dried over appropriate drying agents, distilled, and stored over 4 Å molecular sieve prior to use. Deuterated solvents were dried over appropriate drying agents, distilled, and stored inside a glove box. NMR spectra were recorded on a Bruker Avance III 500 or a Bruker Avance III 500 HD spectrometer. Chemical shifts (in $\delta$, ppm) are referenced to the solvent residual signals [$CDCl_3$: $^1H$, 7.26 and $^{13}C$, 77.7 ppm, $C_6D_6$: $^1H$, 7.16 and $^{13}C$, 128.06 ppm, $CD_2Cl_2$: $^1H$, 5.32 and $^{13}C$, 53.8 ppm].[1] For NMR data, "s" refers to singlet, "d" refers to doublet, "*p*t" refers to *pseudo*-triplet, "t" refers to triplet, "sept" refers to septet, "m" refers to multiplet, and "br" refers to broad signals. Nano-ESI mass spectra were recorded using an Esquire 3000 ion trap mass spectrometer (Bruker Daltonik GmbH, Bremen, Germany) equipped with a nano-ESI source. Samples were dissolved in a suitable solvent and introduced by static nano-ESI using *in-house* pulled glass emitters. Nitrogen served both as nebulizer as well as dry gas and was generated by a Bruker nitrogen generator NGM 11. Helium served as cooling gas for the ion trap. The mass axis was externally calibrated with ESI-L Tuning Mix (Agilent Technologies, Santa Clara, CA, USA) as a calibration standard. Infrared spectra were recorded using a BrukerAlpha-T FT-IR spectrometer. Melting points were measured with a Büchi B-545 Melting Point apparatus. Elemental analyses were carried out with a EURO EA Element Analyzer. Unless stated otherwise, chemicals from commercial suppliers (Merck, Sigma-Aldrich, or TCI) were used as received. $(IPr^{Ph})Br$ (**1**) ($IPr^{Ph}$ = $PhC\{N(2,6\text{-}i\text{Pr}_2C_6H_3)CH\}_2$)[2] and $(Me_2S)AuCl$[3] were prepared according to the reported methods.

### Synthesis of $[(ADC)GaH_2]_2$ (3)

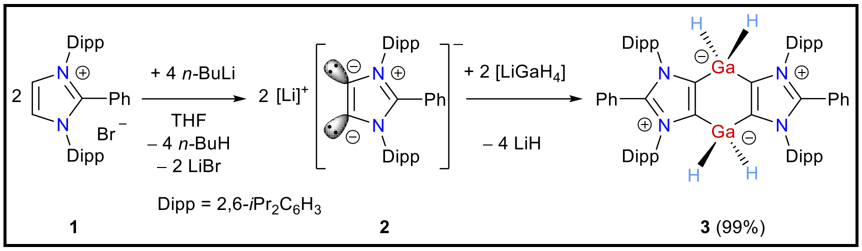


To a 30 mL THF suspension of ($IPr^{Ph}$)Br (**1**) (5.0 g, 9.24 mmol) was added *n*BuLi (8.5 mL, 21.3 mmol, 2.5 M in *n*-hexane) at –30 °C. The reaction mixture was brought to room temperature (rt), stirred for 2h, and then added to a THF slurry of a freshly prepared $LiGaH_4$ (9.70 mmol). [$LiGaH_4$ was prepared by a slight modification of the literature method: $GaCl_3$ (1.71 g, 9.70 mmol) was dissolved in 10 mL THF and added to 10 mL THF suspension of LiH (0.34 g, 42.7 mmol) at –50°C. The reaction mixture was slowly brought to rt and stirred for additional 2h].[4] The reaction mixture was stirred overnight. The volatiles were removed, and the resulting solid was dried *in vacuo* at 150 °C for 3h. The residue was extracted with 75 mL toluene and the solvent was removed from the filtrate to obtain **3** as off-white solid in 99% (4.9 g) yield. Single crystals suitable for X-ray diffraction studies were obtained by a slow diffusion of *n*-hexane into a saturated benzene solution of **3**. M.p.: 370 °C (dec.). Elemental analysis (%) calculated for $C_{66}H_{82}N_4Ga_2$ (1071) **3**: C 74.03, H 7.72, N 5.32; found: C 74.46, H 7.83, N 5.23. **$^1$H NMR** (500 MHz, $C_6D_6$, 298 K): 7.23 (t, $J$ = 7.2 Hz, 4H, *p*-$C_6H_3$), 7.12 (d, $J$ = 7.8 Hz, 8H, *m*-$C_6H_3$), 7.01–6.98 (m, 4H, *o*-$C_6H_5$), 6.59–6.54 (m, 6H, *m*/*p*-$C_6H_5$), 4.16 (s, 4H, Ga$H_2$), 3.22 (sept, $J$ = 6.8 Hz, 8H, C*H*Me$_2$), 1.44 (d, $J$ = 6.8 Hz, 24H, CH*Me*$_2$), 0.85 (d, $J$ = 6.9 Hz, 24H, CH*Me*$_2$) ppm. **$^{13}$C{$^1$H} NMR** (126 MHz, $C_6D_6$, 298 K): 158.3 (*C*Ga), 145.3 (*i*-$C_6H_3$), 144.4 (N*C*N), 136.5, 130.2, 128.9, 128.7, 128.4, 128.0, 125.4, 125.0 ($C_6H_3$/$C_6H_5$), 28.8 (*C*HMe$_2$), 24.4, 23.6 (CH*Me*$_2$) ppm. **ESI-MS** (*m*/*z*.): 1069.4 [**3**–H]$^+$. **FTIR:** 1800, 1830 cm$^{-1}$ (GaH).

### Synthesis of $[(ADC)GaI_2]_2$ (4)

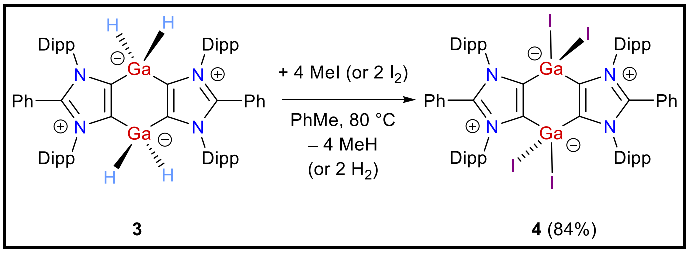


To a 20 mL toluene solution of **3** (0.5 g, 0.47 mmol) was dropwise added MeI (0.14 mL, 2.10 mmol) at rt (*Caution*: Gas evolution). The reaction mixture was stirred at 80 °C overnight. Then, the solvent was

reduced to 5 mL and 20 mL of *n*-hexane was added. The precipitate formed was isolated by filtration, washed with 5 mL *n*-hexane twice, and then dried under reduced pressure to obtain compound **4** as an off-white solid in 84% (0.62 g) yield. Single crystals suitable for X-ray diffraction studies were obtained by a slow diffusion of *n*-hexane into a saturated dichloromethane solution of **4**. M.p.: < 410 °C. Elemental analysis (%) calculated for $C_{66}H_{78}N_4Ga_2I_4$ (1574) **4**: C 50.35, H 4.99, N 3.56; found: C 50.41, H 5.02, N 3.52. $^{1}$**H NMR** (500 MHz, $CDCl_3$, 298 K): 7.44 (t, *J* = 7.8 Hz, 4H, *p*-$C_6H_3$), 7.32 (s, 4H, *m*-$C_6H_3$), 7.19–7.07 (m, 6H, *m*-$C_6H_3$/*p*-$C_6H_5$), 6.97 (t, *J* = 7.8 Hz, 4H, *m*-$C_6H_5$), 6.73 (d, *J* = 8.2 Hz, 4H, *o*-$C_6H_5$), 3.11–2.96 (br, 4H, C*H*Me$_2$), 2.94–2.78 (br, 4H, C*H*Me$_2$), 1.46–1.36 (m, 12H, CH*Me*$_2$), 1.33–1.26 (m, 12H, CH*Me*$_2$), 1.24–1.14 (m, 12H, CH*Me*$_2$), 0.22–0.09 (m, 12H, CH*Me*$_2$) ppm. $^{13}$**C{$^{1}$H} NMR** (126 MHz, $CDCl_3$, 298 K): 164.0 (*C*Ga), 149.0, 145.3, 132.9, 130.9, 130.4, 129.8, 129.2, 128.1, 125.4, 124.6, 123.7 (*C*$_6$H$_3$/*C*$_6$H$_5$), 28.9, 28.4 (*C*HMe$_2$), 26.7, 25.5, 23.9, 23.1 (CH*Me*$_2$) ppm. **ESI-MS** (*m*/*z*.): 1447.0 [**4**–I]$^+$.

**Alternative Synthesis of 6**: $I_2$ (1.0 g, 3.92 mmol) was dissolved in 10 mL $Et_2O$ and added to a 30 mL toluene solution of **3** (2.0 g, 1.86 mmol) at –20 °C. The reaction mixture was slowly brought to rt and stirred overnight. The solution was reduced to 10 mL and then 50 mL *n*-hexane was added. The precipitate formed was recovered by filtration and dried under reduced pressure to obtain **6** as a pale-yellow solid in 80% (2.3 g) yield.

### Synthesis of [(ADC)$_2$Ga(GaI$_2$)] (5)

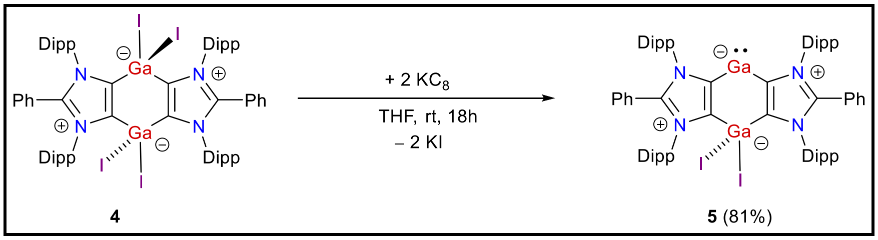


To a mixture of **6** (0.5 g, 0.32 mmol) and $KC_8$ (92 mg, 0.68 mmol) was added 10 ml THF (or $C_6H_6$) and the resulting reaction mixture was stirred overnight at rt. The suspension was filtered through a plug of Celite and the volatiles from the filtrate were removed under vacuum to obtain compound **5** as a brown solid in 81% yield (0.35 g). Elemental analysis (%) calculated for $C_{68}H_{78}N_4Ga_2I_2$ (1321) **5**: C 60.03, H 5.95, N 4.24; found: C 58.37, H 6.12, N 4.11. $^{1}$**H NMR** (500 MHz, $C_6D_6$, 298 K): 7.22 (m, 6H, *p*-$C_6H_3$/*p*-$C_6H_5$), 7.13 (d, *J* = 7.8 Hz, 4H, *m*-$C_6H_3$), 7.08–7.04 (m, 6H, $C_6H_5$/$C_6H_3$), 6.60–6.57 (m, 6H, $C_6H_5$), 3.26 (sept, *J* = 6.8 Hz, 4H, C*H*Me$_2$), 3.09 (sept, *J* = 6.8 Hz, 4H, C*H*Me$_2$), 1.62 (d, *J* = 6.7 Hz, 12H, CH*Me*$_2$), 1.35 (d, *J* = 6.8 Hz, 12H, CH*Me*$_2$), 0.92 (d, *J* = 6.9 Hz, 12H, CH*Me*$_2$), 0.87 (d, *J* = 6.8 Hz, 12H, CH*Me*$_2$) ppm. $^{13}$**C NMR** (126 MHz, $C_6D_6$) 176.4 (*C*Ga), 163.6 (*C*GaI$_2$), 145.8, 145.5, 143.7, 136.0, 134.6, 130.7, 130.3, 129.6, 125.2, 125.0, 124.5 (*C*$_6$H$_3$/*C*$_6$H$_5$), 29.1, 29.0 (*C*HMe$_2$), 26.1, 25.2, 23.5, 23.2 (CH*Me*$_2$) ppm.

**ESI-MS** (*m*/*z*.): 1321.3 [**5**+H]$^+$. **UV-Vis** [THF, 298 K: $\lambda_{max}$ (nm) ($\varepsilon$ ($M^{-1}$ $cm^{-1}$))]: 300 (16200), 370 (2150), 500 (600).

**Synthesis of [(ADC)Ga]$_2$ (6)**

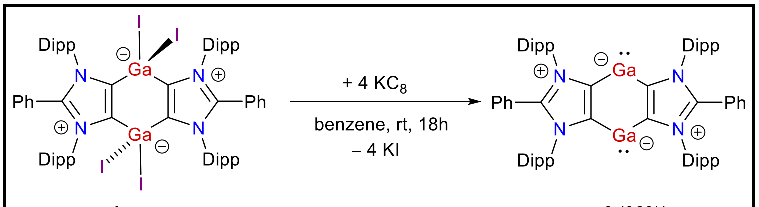


To a solid mixture of **6** (1.50 g, 0.95 mmol) and $KC_8$ (0.71 g, 5.24 mmol) was added 40 mL benzene at rt and then stirred overnight. The resulting suspension was filtered through a plug of Celite and washed with 20 ml of benzene. The volatiles from the filtrate were removed under reduced pressure to obtain compound **6** as a red-brown solid in 92% yield (0.94 g). Single crystals suitable for X-ray diffraction studies were obtained by storing a saturated toluene solution of **6** at –30°C for two days. M.p.: 150°C (dec.). Elemental analysis (%) calculated for $C_{68}H_{78}N_4Ga_2$ (1067) **6**: C 74.31, H 7.37, N 4.85; found: C 73.61, H 6.94, N 4.53. **$^1$H NMR** (500 MHz, $C_6D_6$, 298 K): 7.23 (t, *J* = 7.7 Hz, 4H, *p*-$C_6H_3$), 7.18–7.16 (m, 4H, *o*-$C_6H_5$), 7.09 (d, *J* = 7.8 Hz, 8H, *m*-$C_6H_3$), 6.71–6.59 (m, 6H, *p*/*m*-$C_6H_5$), 3.10 (sept, *J* = 6.8 Hz, 8H, C*H*Me$_2$), 1.30 (d, *J* = 6.7 Hz, 24H, CH*Me*$_2$), 0.97 (d, *J* = 6.9 Hz, 24H, CH*Me*$_2$) ppm. **$^{13}$C{$^1$H} NMR** (126 MHz, $C_6D_6$, 298 K): 173.6 (*C*Ga), 145.5 (*p*-$C_6H_3$), 142.6 (N*C*N), 137.0, 129.6, 129.5, 128.7, 128.0, 126.4, 124.2 (*C*$_6$H$_3$/*C*$_6$H$_5$), 29.1 (*C*HMe$_2$), 25.2, 23.3 (CH*Me*$_2$) ppm. **ESI-MS** (*m*/*z*.): 1067.4 [**6**+H]$^+$. **UV-Vis** [$C_6H_6$, 298 K: $\lambda_{max}$ (nm) ($\varepsilon$ ($M^{-1}$ $cm^{-1}$))]: 278 (3100), 312 (3720), 330 (3040), 380 (580), 560 (120).

**Synthesis of [(ADC)Ga{Fe(CO)$_4$}]$_2$ (7)**

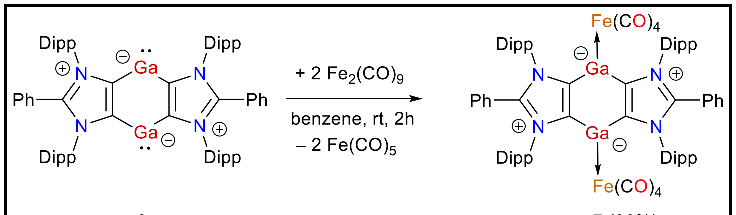


To a mixture of **6** (20 mg, 0.019 mmol) and $Fe_2(CO)_9$ (13.9 mg, 0.038 mmol) was added 1 mL benzene and stirred for 2h at rt. The suspension was filtered and the volatiles from the filtrate were removed to afford **7** as an orange solid in 86% yield (23.0 mg). Single crystals suitable for X-ray diffraction studies were obtained by a slow diffusion of *n*-hexane into a saturated benzene solution of **7**. Elemental analysis (%) calculated for $C_{68}H_{78}N_4Ga_2$ (1403) **7**-Fe: C 63.37, H 5.61, N 3.99; found: C 62.34, H 5.43, N 3.81. **$^1$H**

**NMR** (500 MHz, $C_6D_6$, 298 K): 7.27 (t, $J$ = 7.8 Hz, 4H, $p$-$C_6H_3$), 7.07 (d, $J$ = 7.8 Hz, 8H, $m$-$C_6H_3$), 6.58–6.52 (m, 6H, $m/p$-$C_6H_5$), 6.49 (t, $J$ = 7.7 Hz, 4H, $o$-$C_6H_5$), 2.88–2.75 (br, 8H, C*H*Me$_2$), 1.54 (br, 24H, CH*Me*$_2$), 0.70 (br, 24H, CH*Me*$_2$) ppm. **$^{13}$C{$^1$H} NMR** (126 MHz, $C_6D_6$, 298 K): 216.6 (*C*O), 148.6, 146.0, 132.9, 132.0, 130.6, 130.0, 128.6, 126.0, 124.6 ($C_6H_3$/$C_6H_5$), 29.1 (*C*HMe$_2$), 26.1, 23.5 (CH*Me*$_2$) ppm. **ESI-MS** (*m/z*.): 1409.3 [**7**-Fe+Li]$^+$.

## Synthesis of [(ADC)GaCl$_2$]$_2$ (8)

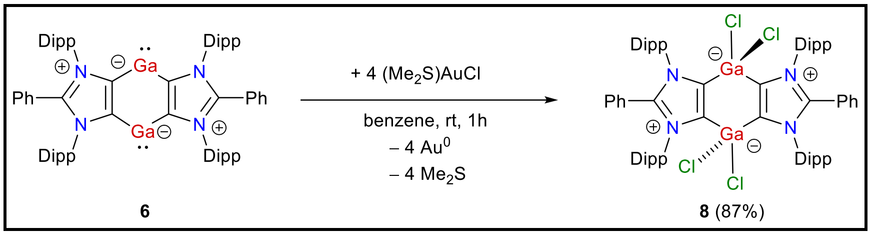


To a mixture of **6** (20 mg, 0.019 mmol) and (Me$_2$S)AuCl (23 mg, 0.076 mmol) was added 1 mL benzene and then stirred overnight at rt. The suspension was filtered, and the volatiles were removed from the filtrate under reduced pressure to obtain compound **8** as an off-white solid in 87% (20 mg) yield. Single crystals suitable for X-ray diffraction were obtained by a slow diffusion of *n*-hexane into a saturated benzene solution of **8**. Elemental analysis (%) calculated for $C_{66}H_{78}N_4Cl_4Ga_2$ (1209) **8**: C 65.59, H 6.51, N 4.64; found: C 65.67, H 6.40, N 4.55. **$^1$H NMR** (500 MHz, $C_6D_6$, 298 K): 7.23–7.18 (m, 4H, $p$-$C_6H_3$), 7.10 (d, $J$ = 7.8 Hz, 8H, $m$-$C_6H_3$), 6.98–6.95 (m, 4H, $o$-$C_6H_5$), 6.58–6.54 (m, 2H, $p$-$C_6H_5$), 6.52–6.48 (m, 4H, $m$-$C_6H_5$), 3.17 (sept, $J$ = 6.7 Hz, 8H, C*H*Me$_2$), 1.58 (d, $J$ = 6.6 Hz, 24H, CH*Me*$_2$), 0.81 (d, $J$ = 6.9 Hz, 24H, CH*Me*$_2$) ppm. **$^{13}$C NMR** (126 MHz, $C_6D_6$) 153.5 (*C*Ga), 145.7, 134.0, 131.0, 130.3, 129.6, 125.1, 124.4 (*C*$_6$H$_3$/*C*$_6$H$_5$), 29.2 (*C*HMe$_2$), 25.2, 23.5 (CH*Me*$_2$) ppm. **ESI-MS** (*m/z*.): 1315.3 [**10**–Cl]$^+$.

## Synthesis of [(ADC)Ga(P$_4$)]$_2$ (9)

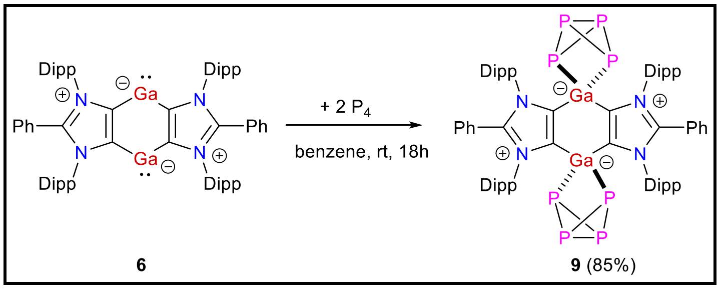


A mixture of **6** (0.10 g, 0.094 mmol) and $P_4$ (30 mg, 0.24 mmol) was suspended in 5 mL benzene and stirred overnight. The resulting suspension was filtered and the volatiles from the filtrate were removed under vacuum to give **9** as an orange-brown solid in 85% yield (105 mg). Elemental analysis (%) calculated for $C_{68}H_{78}N_4Ga_2P_8$ (1315) **9**: C 60.30, H 5.98, N 4.26; found: C 59.22, H 5.63, N 4.01. **$^1$H**

**NMR** (500 MHz, $C_6D_6$, 298 K): 7.50–7.40 (m, 12H, $C_6H_3$), 6.91–6.86 (m, 4H, *o*-$C_6H_5$), 6.67–6.54 (m, 6H, *m*/*p*-$C_6H_5$), 3.23 (sept, *J* = 6.8 Hz, 8H, C*H*Me$_2$), 1.70 (d, *J* = 6.7 Hz, 24H, CH*Me*$_2$), 0.67 (d, *J* = 6.8 Hz, 24H, CH*Me*$_2$) ppm. **$^{13}$C{$^1$H} NMR** (126 MHz, $C_6D_6$, 298 K): 157.8 (*C*Ga), 145.1 (N*C*N), 145.1 (*i*-$C_6H_3$), 136.0, 131.8, 130.1, 130.1, 130.0, 128.6, 127.3, 125.5 (*C*$_6$H$_3$/*C*$_6$H$_5$), 29.8 (*C*HMe$_2$), 24.8, 23.6 (CH*Me*$_2$) ppm. **$^{31}$P NMR** (202 MHz, $C_6D_6$, 298 K) 152.3 (t, *J* = 157 Hz), –298.7 (t, *J* = 157 Hz) ppm. **ESI-MS** (*m*/*z*.): 1315.3 [**8**+H]$^+$.

### Synthesis of [(ADC)GaF(Tol$^F$)]$_2$ (*cis*-/*trans*-10-Tol$^F$)

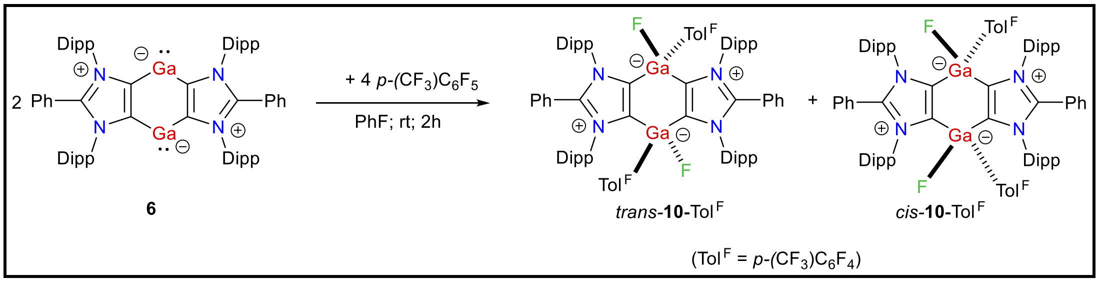

To a 5 mL fluorobenzene solution of **6** (0.20 g, 0.187 mmol) was added *p*-($CF_3$)$C_6F_5$ (60 µL, 0.412 mmol) at rt. The reaction mixture was stirred 2h and the volatiles were removed under reduced pressure to obtain a beige solid. NMR analyses indicated complete consumption of **6** and quantitative formation of *cis*-/*trans*-**10**-Tol$^F$ as a mixture (~1:1 based on $^1$H NMR). The residue containing a mixture of *cis*-/*trans*-**10**-Tol$^F$ was washed with 25 mL *n*-hexane and dried under reduced pressure to obtain a pure sample of *cis*-**10**-Tol$^F$ as an off-white solid. Elemental analysis (%) calculated for $C_{80}H_{78}N_4F_{16}Ga_2$ (1539) *cis*-**10**-Tol$^F$: C 62.44, H 5.11, N 3.64; found: C 62.67, H 5.20, N 3.55. **$^1$H NMR** (500 MHz, $C_6D_6$, 298 K): 7.14 (d, *J* = 4.8 Hz, 8H, *m*-$C_6H_3$), 6.81 (t, *J* = 4.7 Hz, 4H, *p*-$C_6H_3$), 6.73 (d, *J* = 8.1 Hz, 4H, *o*-$C_6H_5$), 6.54 (t, *J* = 7.4 Hz, 2H, *p*-$C_6H_5$), 6.45 (t, *J* = 8.0 Hz, 4H, *m*-$C_6H_5$), 3.00 (sept, *J* = 6.7 Hz, 4H, C*H*Me$_2$), 2.89 (sept, *J* = 6.7 Hz, 4H, C*H*Me$_2$), 1.66 (d, *J* = 6.7 Hz, 12H, CH*Me*$_2$), 0.74 (d, *J* = 6.8 Hz, 12H, CH*Me*$_2$), 0.52 (*p*t, *J* = 6.8 Hz, 24H, CH*Me*$_2$) ppm. **$^{13}$C{$^1$H} NMR** (126 MHz, $C_6D_6$, 298 K): 146.4, 145.9, 143.6, 134.4, 131.2, 130.3, 129.5, 128.3, 128.1, 125.6, 124.6, 124.4 (*C*$_6$H$_3$/*C*$_6$H$_5$/*C*$_7$F$_7$), 29.2, 28.8 (*C*HMe$_2$), 24.3, 24.2, 23.7, 23.3 (CH*Me*$_2$), 21.8 (*C*F$_3$) ppm. **$^{19}$F{$^1$H} NMR** (471 MHz, $C_6D_6$, 298 K): –56.2 (t, *J* = 21.3 Hz, C*F*$_3$), –120.4, –143.3 (C$_6$*F*$_4$), –205.6 (Ga*F*) ppm. **ESI-MS** (*m*/*z*.): 1561.4 [**10**-Tol$^F$+Na]$^+$.

Single crystals suitable of *trans*-**10**-Tol$^F$ for X-ray diffraction were obtained by a slow diffusion of *n*-hexane into a dichloromethane solution of *cis*-/*trans*-**10**-Tol$^F$.

### Synthesis of [(ADC)GaF($C_6F_5$)]$_2$ (*cis*-/*trans*-10-$C_6F_5$)

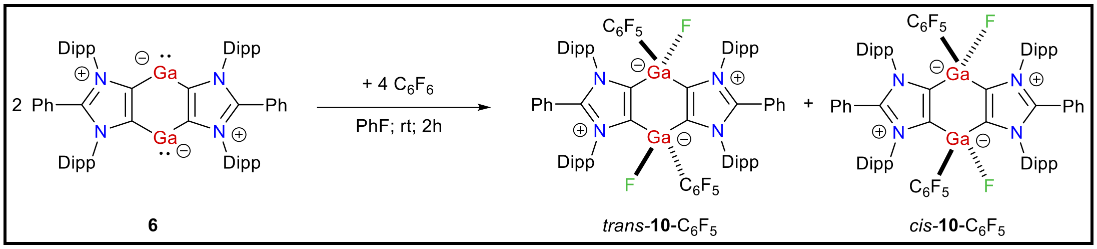


To a 5 mL fluorobenzene solution of **6** (200 mg, 0.187 mmol) was added $C_6F_6$ (46 µL, 0.399 mmol) at rt. The reaction mixture was stirred 2h and then the volatiles were removed under reduced pressure to give a beige solid. NMR analyses showed complete consumption of **6** and quantitative formation of a mixture of *cis*-/*trans*-**10**-$C_6F_5$ isomers (in ~4:1 ratio based on $^1$H NMR). Compounds *trans*-**10**-$C_6F_5$ was obtained in a pure form by recrystallization on storing a toluene solution of a mixture of *cis*- and *trans*-**10**-$C_6F_5$ at –24 °C. Single crystals suitable for X-ray diffraction were obtained by a slow diffusion of *n*-hexane into a saturated THF solution of *cis*-**10**-$C_6F_5$. NMR details are given for *cis*-**10**-$C_6F_5$. **$^1$H NMR** (500 MHz, $C_6D_6$, 298 K): 7.37 (d, $J$ = 7.78 Hz, 4H, *m*-$C_6H_3$), 7.16 (m, 4H, *p*-$C_6H_3$), 6.80 (d, $J$ = 8.07 Hz, 4H, *m*-$C_6H_3$), 6.71 (d, $J$ = 8.07 Hz, 4H, *o*-$C_6H_5$), 6.49 (t, $J$ = 7.17 Hz, 2H, *p*-$C_6H_5$), 6.41 (t, $J$ = 7.65 Hz, 4H, *m*-$C_6H_5$), 3.85 (m, 4H, C*H*Me$_2$), 2.29 (m, 4H, C*H*Me$_2$), 1.88 (d, $J$ = 6.8 Hz, 12H, CH*Me*$_2$), 1.17 (d, $J$ = 6.8 Hz, 12H, CH*Me*$_2$), 0.32 (d, $J$ = 6.8 Hz, 12H, CH*Me*$_2$), 0.06 (d, $J$ = 6.8 Hz, 12H, CH*Me*$_2$) ppm. **$^{13}$C{$^1$H} NMR** (126 MHz, $C_6D_6$, 298 K): 146.1, 142.7, 133.7, 130.8, 130.2, 129.4, 128.0, 125.3, 124.54 (*C*$_6$H$_3$, *C*$_6$H$_5$, *C*$_6$F$_5$), 29.3, 28.9, 27.9 (*C*HMe$_2$), 25.6, 23.3, 22.5, 22.3 (CH*Me*$_2$) ppm. **$^{19}$F{$^1$H} NMR** (471 MHz, $C_6D_6$, 298 K): –139.1, –154.1, –158.2, –162.2 (C$_6$*F*$_5$), –207.79 (Ga*F*) ppm.

### Synthesis of [(ADC)GaF($C_5NF_4$)]$_2$ (*cis*-/*trans*-10-$C_5NF_4$)

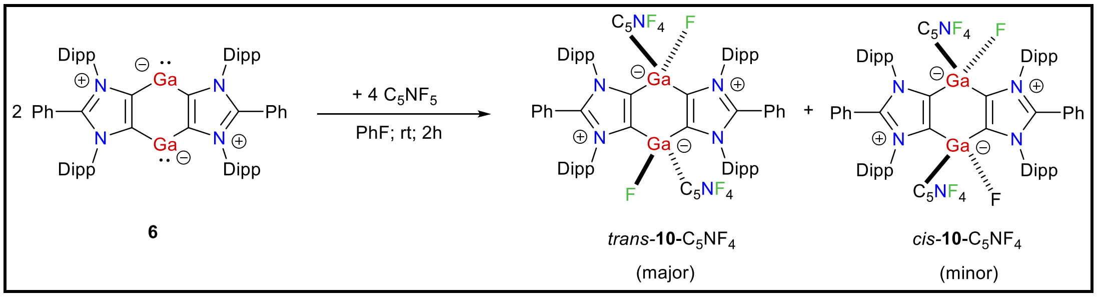


To a 5 mL fluorobenzene solution of **6** (200 mg, 0.187 mmol) was added $C_5NF_5$ (43 µL, 0.395 mmol) at rt. The reaction mixture was stirred for 2h and then the volatiles were removed under reduced pressure to afford a beige solid. NMR analyses showed complete consumption of **6** and quantitative formation of a

mixture of *cis-/trans*-**10**-$C_5NF_4$ isomers (in ~1:1 ratio based on $^1$H NMR). Compounds *trans*-**10**-$C_5NF_4$ was obtained in a pure form by recrystallization on storing a toluene solution of a mixture of *cis*- and *trans*-**10**-$C_5NF_4$ at –24 °C. NMR data are given for *trans*-**10**-$C_5NF_4$. **$^1$H NMR** (500 MHz, $CDCl_3$, 298 K): 7.40 (m, 8H, *m*-$C_6H_3$, *p*-$C_6H_3$), 7.18 (t, *J* = 7.5 Hz, 2H, *p*-$C_6H_5$), 6.91 (m, 8H, *m*-$C_6H_3$, *m*-$C_6H_5$), 6.64 (d, *J* = 7.5 Hz, 4H, *o*-$C_6H_5$), 3.38 (m, 4H, C*H*$Me_2$), 2.06 (m, 4H, C*H*$Me_2$), 1.57 (d, *J* = 6.8 Hz, 12H, CH*Me*$_2$), 1.04 (d, *J* = 6.8 Hz, 12H CH*Me*$_2$), 0.22 (d, *J* = 6.8 Hz, CH*Me*$_2$), –0.08 (d, *J* = 6.8 Hz, CH*Me*$_2$) ppm. **$^{13}$C{$^1$H} NMR** (126 MHz, $CDCl_3$, 298 K): 146.0, 145.7, 142.7, 137.9, 134.0, 131.1, 130.5, 129.5, 129.0, 128.2, 128.1, 125.5, 125.3, 124.7, 123.8 (*C*$_6H_3$, *C*$_6H_5$, $C_5NF_4$), 29.0, 27.9 (*C*H$Me_2$), 23.4, 22.6, 22.4, 21.5 (CH*Me*$_2$) ppm. **$^{19}$F{$^1$H} NMR** (471 MHz, $CDCl_3$, 298 K): –96.6, –126.7 ($C_5N$*F*$_4$), –211.8 (Ga*F*) ppm. Single crystals suitable for X-ray diffraction were obtained by a slow diffusion of *n*-hexane into a saturated THF solution of *trans*-**10**-$C_5NF_4$.

### Synthesis of [(ADC)GaF($C_6HF_4$)]$_2$ (*cis-/trans*-10-$C_6HF_4$)

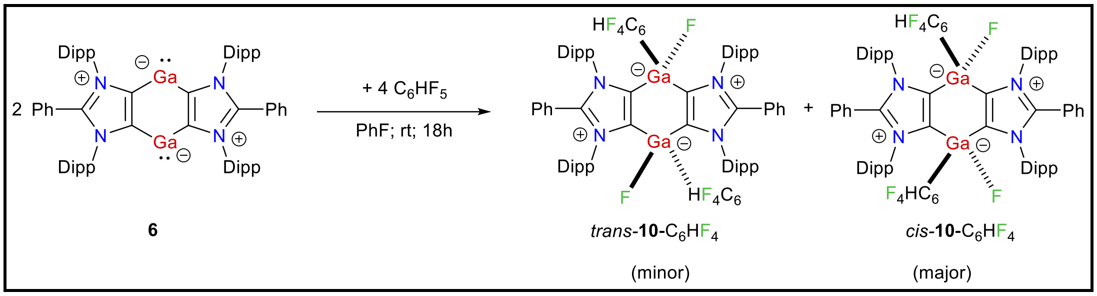


To a 3 mL fluorobenzene solution of **6** (200 mg, 0.187 mmol) was added $C_6HF_5$ (44 µL, 0.399 mmol) at –30 °C. The reaction mixture was stirred for 2h and then the volatiles were removed under reduced pressure to afford a beige solid. NMR analyses showed complete consumption of **6** and quantitative formation of a mixture of *cis-/trans*-**10**-$C_6HF_4$ isomers (in ~1:1 ratio based on $^1$H NMR), which could not be separated. The NMR detail is given for a sample containing a mixture of *cis-/trans*-**10**-$C_6HF_4$. **$^1$H NMR** (500 MHz, $CDCl_3$, 298 K): 7.40, 7.32, 7.00, 6.91, 6.83, 6.42–6.55 ($C_6$*H*$_3$, $C_6$*H*$_5$, $C_6$*H*$F_4$), 3.94 (m, 4H, C*HMe*$_2$), 3.06 (m, 8H, C*HMe*$_2$), 2.35 (m, 4H, C*HMe*$_2$), 1.92 (d, *J* = 6.8 Hz, 12H, CH*Me*$_2$), 1.70 (d, *J* = 6.8 Hz, 12H, CH*Me*$_2$), 1,21 (d, *J* = 6.8 Hz, 12H, CH*Me*$_2$), 0.77 (d, *J* = 6.8 Hz, 12H, CH*Me*$_2$), 0.61 (d, *J* = 6.8 Hz, 24H, CH*Me*$_2$), 0.35 (d, *J* = 6.8 Hz, 12H, CH*Me*$_2$), 0.01 (d, *J* = 6.8 Hz, 12H, CH*Me*$_2$) ppm. **$^{19}$F{$^1$H} NMR** (471 MHz, $C_6D_6$, 298 K): –122.0, –124.4, –141.6 ($C_6H$*F*$_4$), –205.3, –209.0 (Ga*F*) ppm.

**Synthesis of $[(ADC)GaN(Ph)N{=}C_6H_5]_2$ (11)**

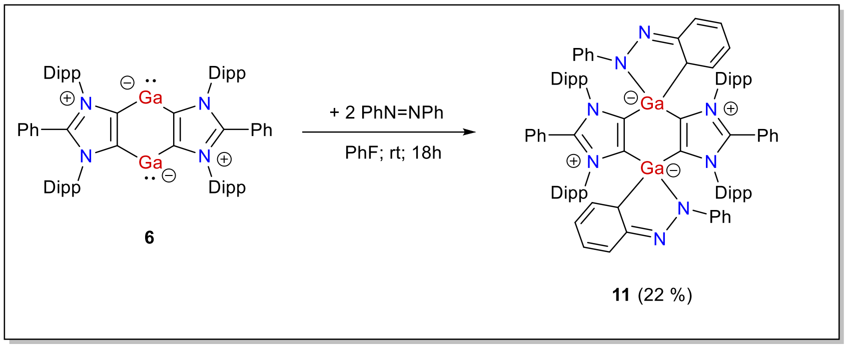


To a 5 mL fluorobenzene solution of **6** (200 mg, 0.187 mmol) was added 3 mL fluorobenzene solution of PhN=NPh (72 mg, 0.394 mmol) at –30 °C. The reaction mixture was stirred overnight at rt. The volatiles were removed under reduced pressure and the resulting residue was washed with 3 mL *n*-hexane and then with 3 mL toluene. The yellow insoluble solid was dried under vacuum to obtain **11** in 22% (69 mg) yield. **$^{1}$H NMR** (500 MHz, $CD_2Cl_2$, 298 K): 7.43, 7.38, 7.31, 7.22, 7.08, 7.01, 6.92, 6.86, 6.78, 6.69, 6.45 ($C_6H_3$, $C_6H_5$), 6.22 (m, 1H, $NC_6H_5Ga$), 6.03, 5.65 (m, 3H, $NC_6H_5Ga$), 5.49 (m, 4H, $NC_6H_5Ga$), 5.43 (m, 2H, $NC_6H_5Ga$), 3.55 (m, 2H, C*H*Me$_2$), 3.45 (m, 2H, C*H*Me$_2$) , 3.27 (m, 2H, C*H*Me$_2$) , 3.17 (m, 2H, C*H*Me$_2$), 1.70 (m, 3H, CH*Me*$_2$), 1.59 (m, 6H, CH*Me*$_2$), 1.46 (m, 3H, CH*Me*$_2$), 1.06 (m, 3H, CH*Me*$_2$), 0.97 (m, 6H, CH*Me*$_2$), 0.73 (m, 6H, CH*Me*$_2$), 0.56 (m, 6H, CH*Me*$_2$, 0.34 (m, 3H, CH*Me*$_2$), −0.04 (m, 3H, CH*Me*$_2$), −0.18 (m, 3H, CH*Me*$_2$), −0.25 (m, 3H, CH*Me*$_2$), −0.38 (m, 3H, CH*Me*$_2$) ppm. **$^{13}$C{$^{1}$H} NMR** (126 MHz, CD2Cl$_2$, 298 K): 135.6, 131.0, 130.1, 129.7, 128.1, 126.2, 125.2, 123.3, 120.7, 114.8, 113.9, 108.7, 108.4 ($C_6H_3$, $C_6H_5$, $NC_6H_5$), 24,6, 24.1, 23.5, 23.3, 23.0, 22.9, 22.7, 22.2 (CH*Me*$_2$) ppm (due to poor solubility, of **11**, the $^{13}$C NMR signals were assigned according to a HMQC experiment).

# Plots of NMR Spectra

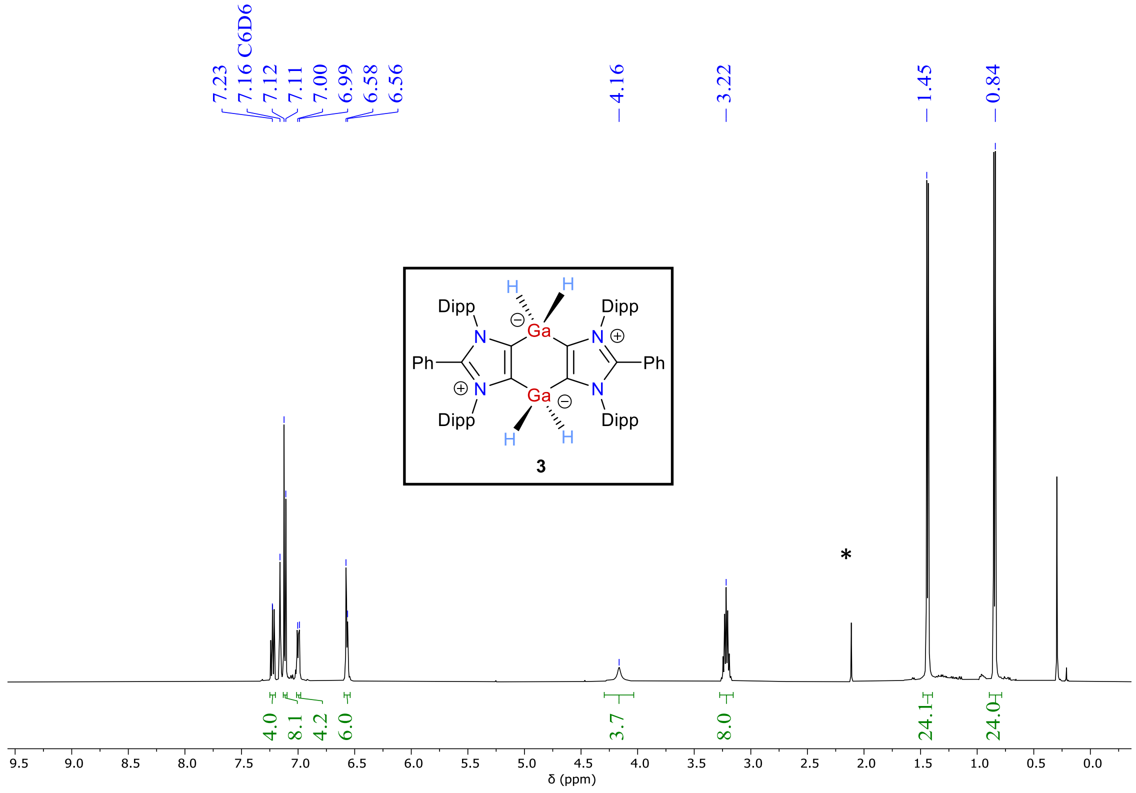

**Figure S1.** $^{1}H$ NMR (500 MHz, $C_6D_6$, 298 K) spectrum of **3** (*toluene).

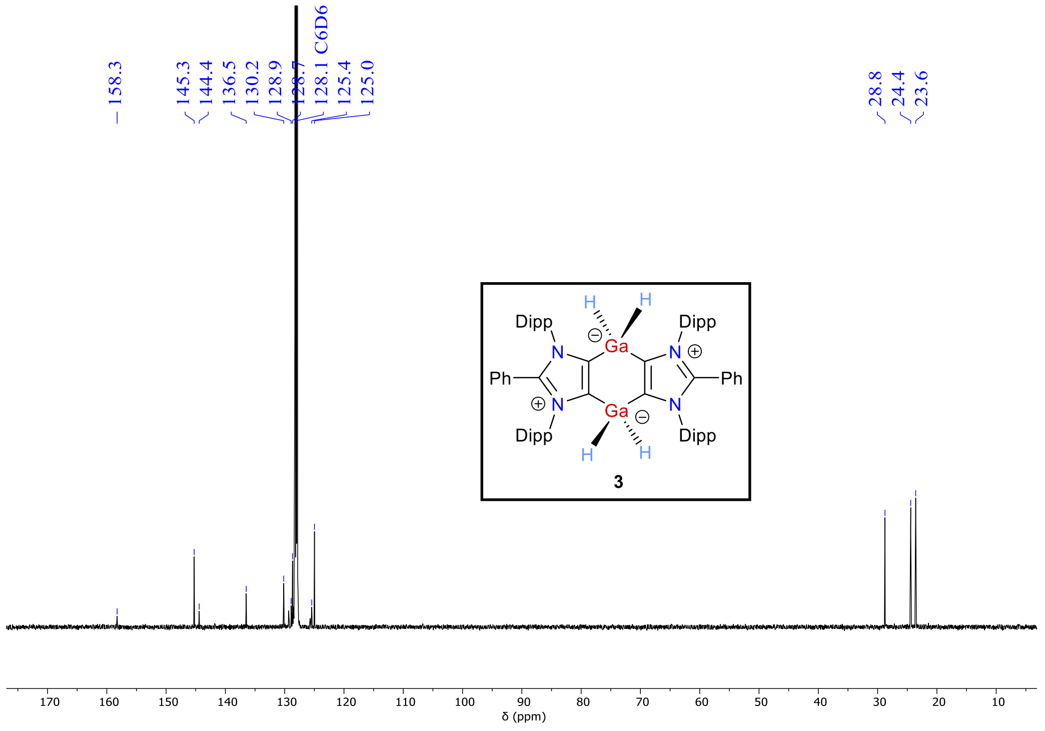

**Figure S2.** $^{13}C\{^{1}H\}$ NMR (126 MHz, $C_6D_6$, 298 K) spectrum of **3**.

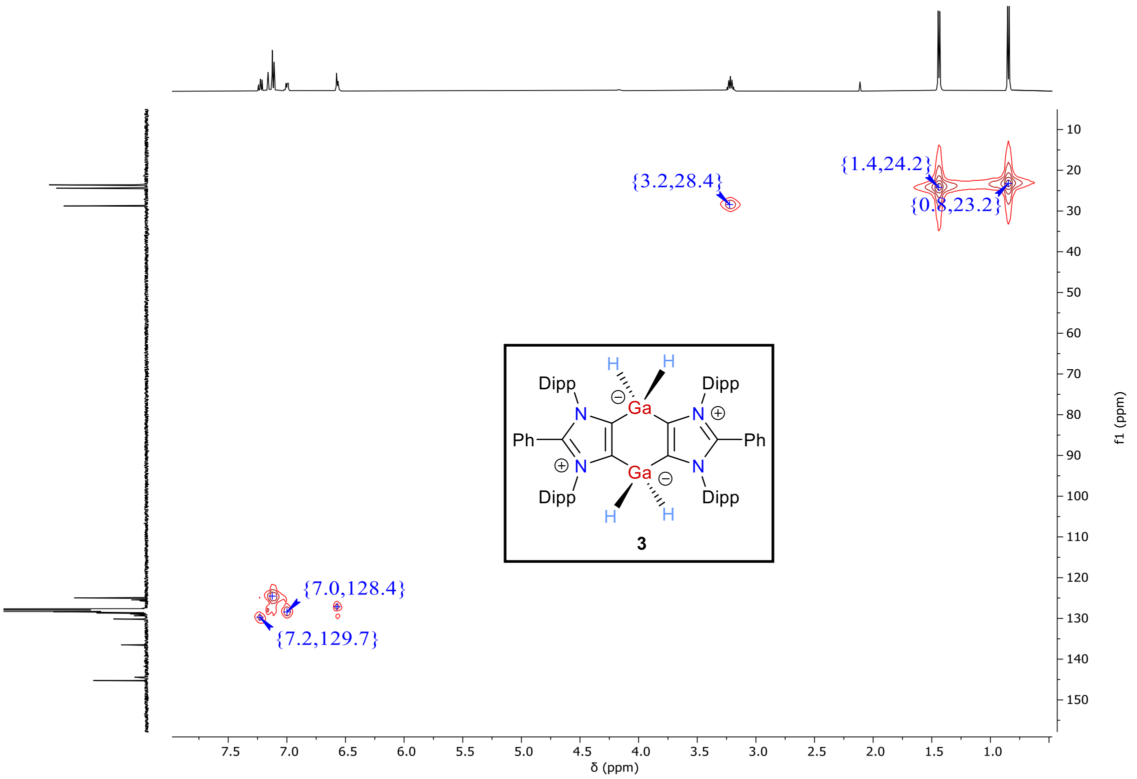


**Figure S3.** $^{1}H$ -$^{13}C\{^{1}H\}$ HMQC (500/126 MHz, $C_6D_6$, 298 K) spectrum of **3**.

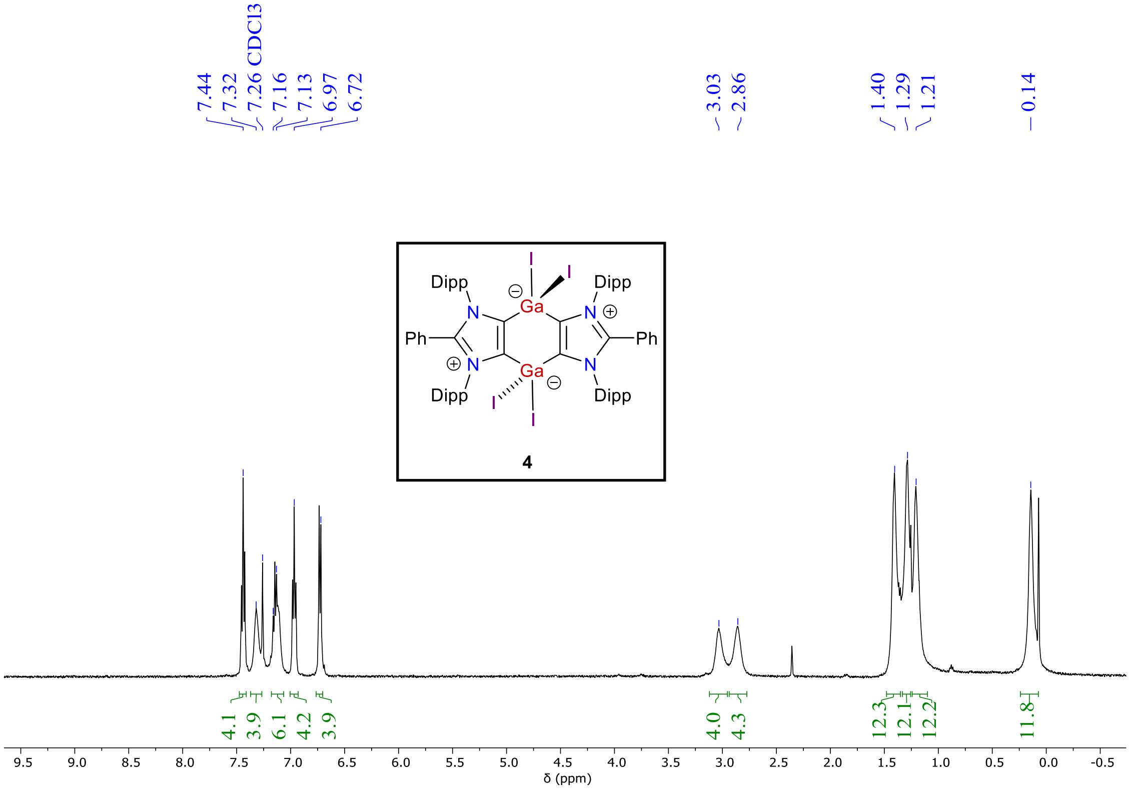


**Figure S4.** $^{1}H$ NMR (500 MHz, $CDCl_3$, 298 K) spectrum of **4**.

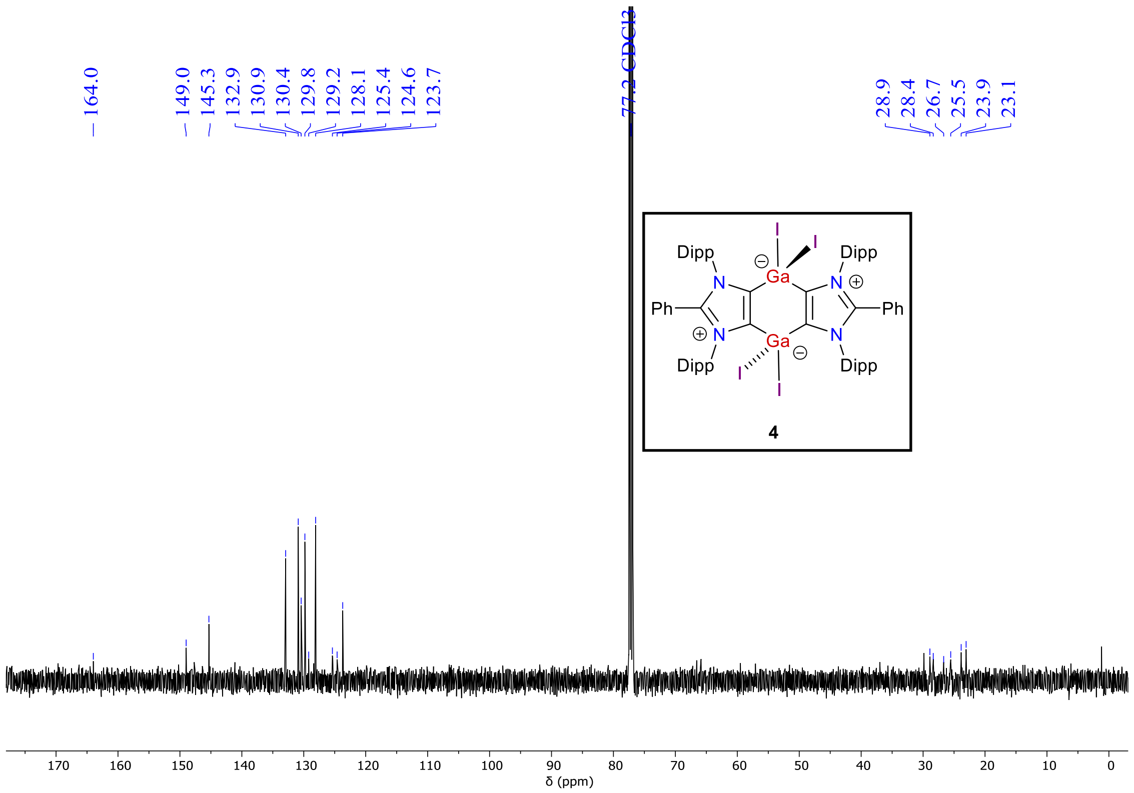


**Figure S5.** $^{13}C\{^{1}H\}$ NMR (126 MHz, $CDCl_3$, 298 K) spectrum of **4**.

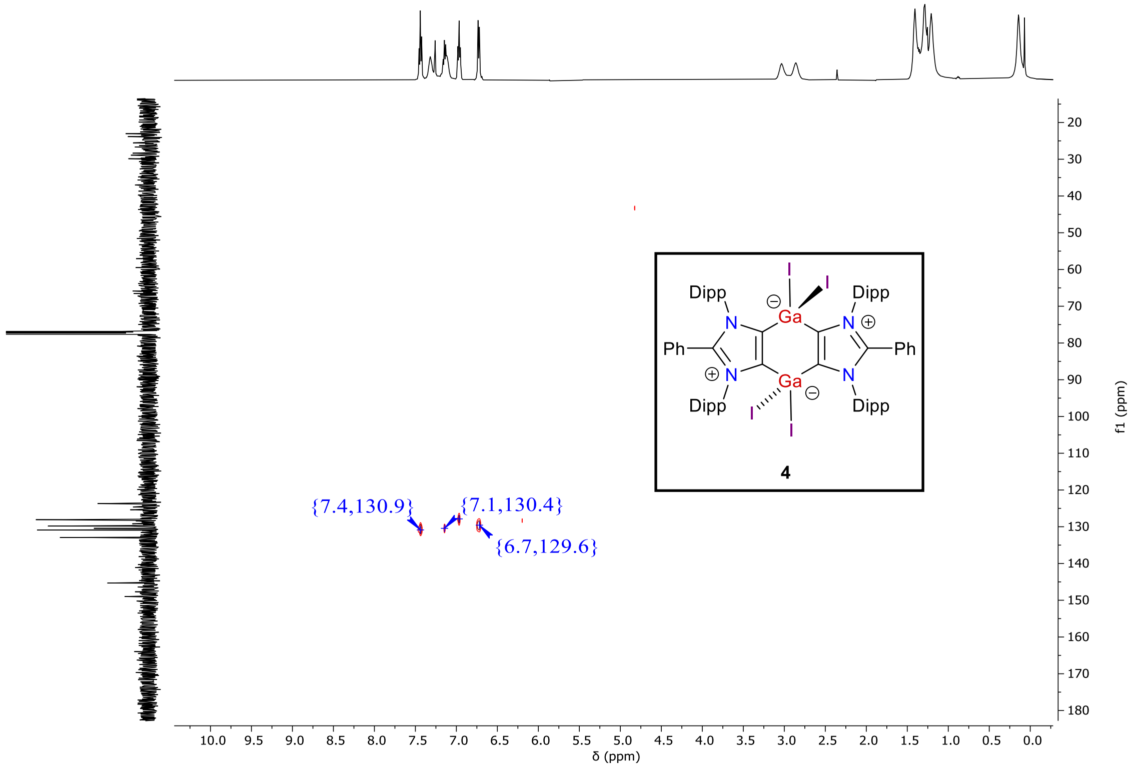


**Figure S6.** $^{1}H$-$^{13}C\{^{1}H\}$ HMQC (500/126 MHz, $CDCl_3$, 298 K) spectrum of **4**.

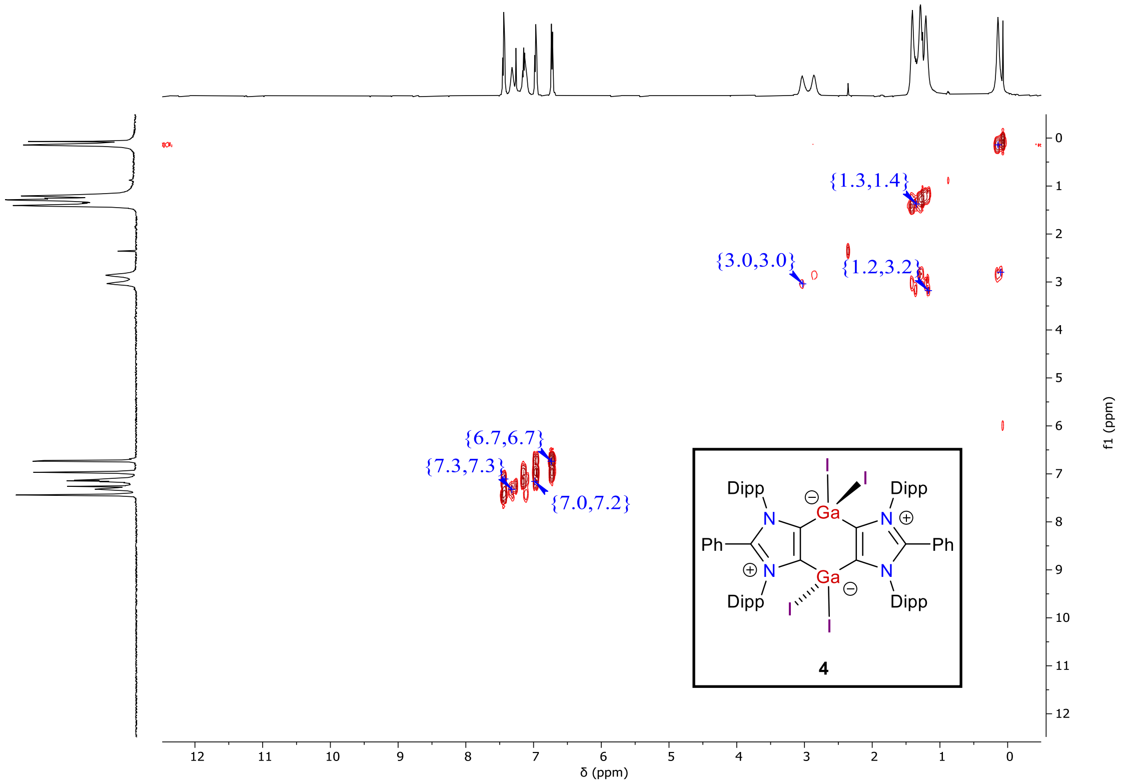


**Figure S7.** $^{1}$H -COSY (500/500 MHz, $CDCl_3$, 298 K) spectrum of **4**.

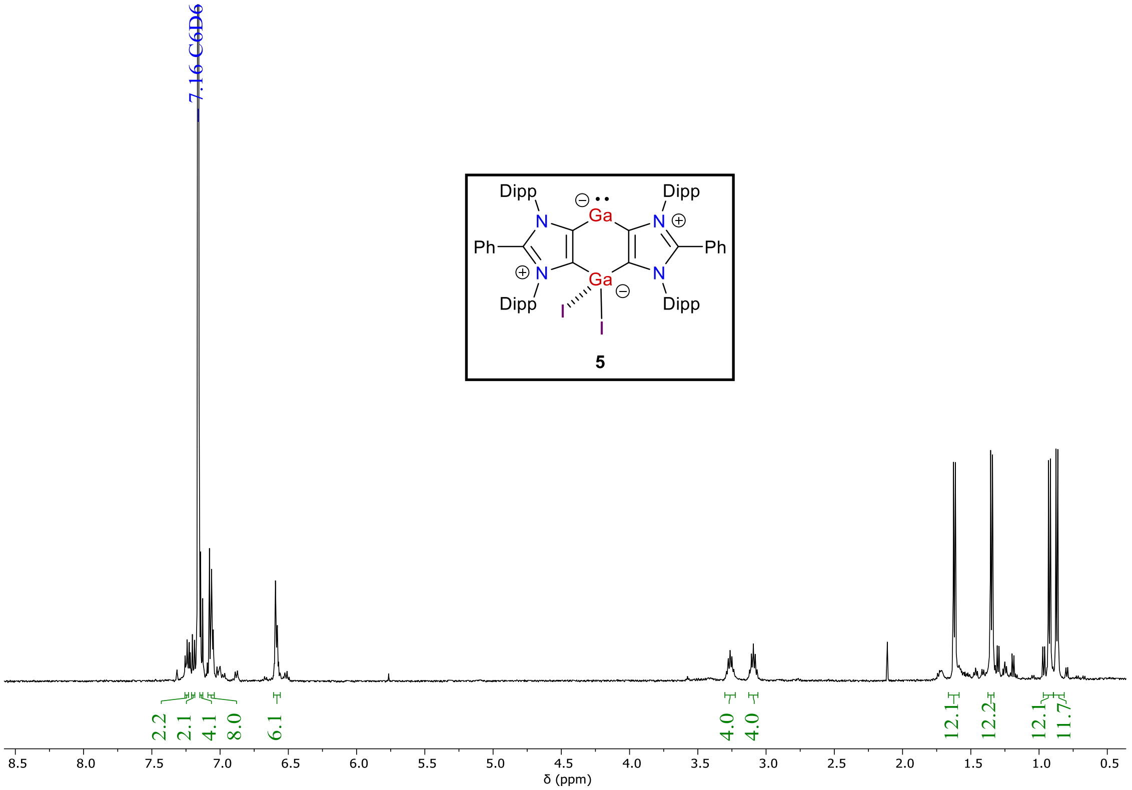


**Figure S8.** $^{1}$H NMR (500 MHz, $C_6D_6$, 298 K) spectrum of **5**.

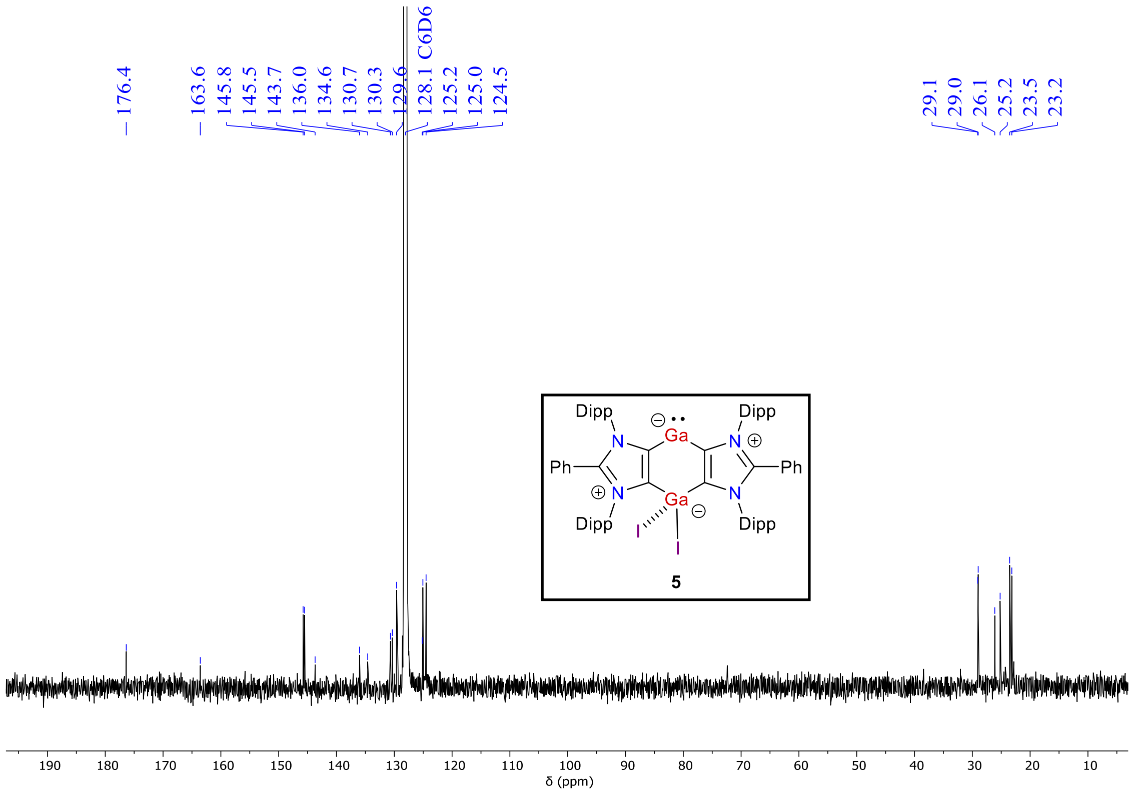


**Figure S9.** $^{13}C\{^{1}H\}$ NMR (126 MHz, $C_6D_6$, 298 K) spectrum of **5**.

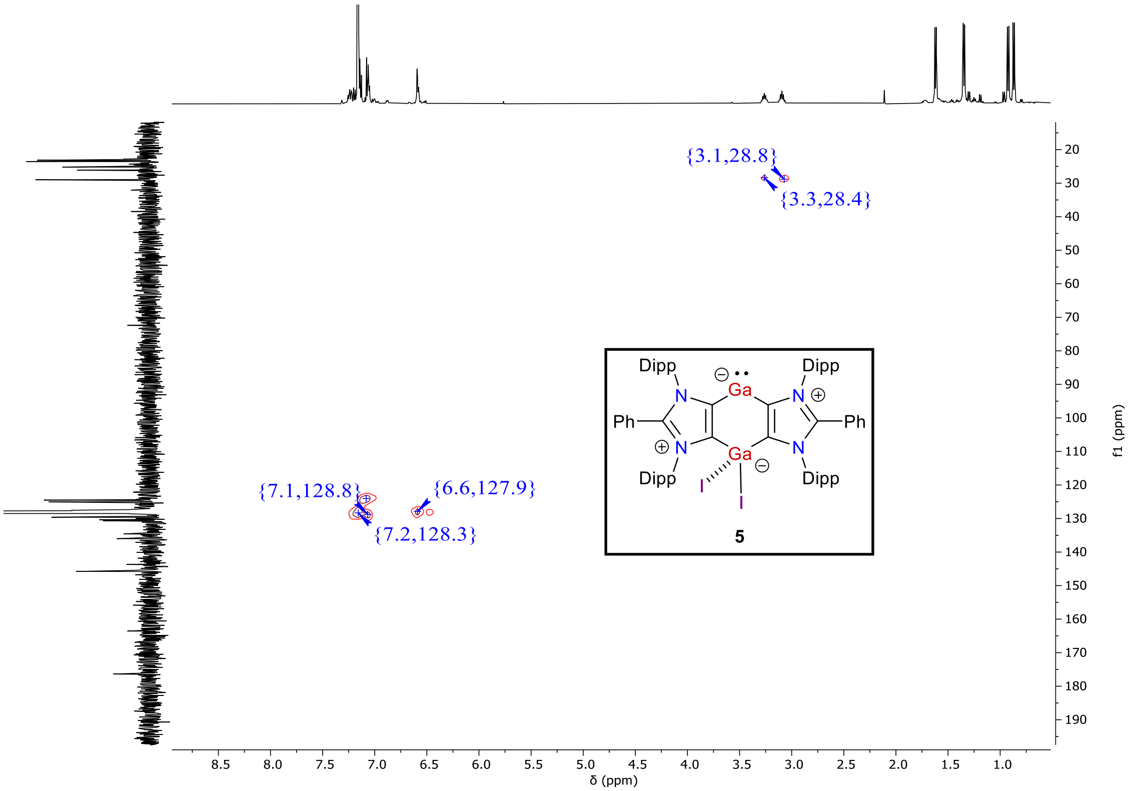


**Figure S10.** $^{1}H$-$^{13}C\{^{1}H\}$ HMQC (500/126 MHz, $C_6D_6$, 298 K) spectrum of **5**.

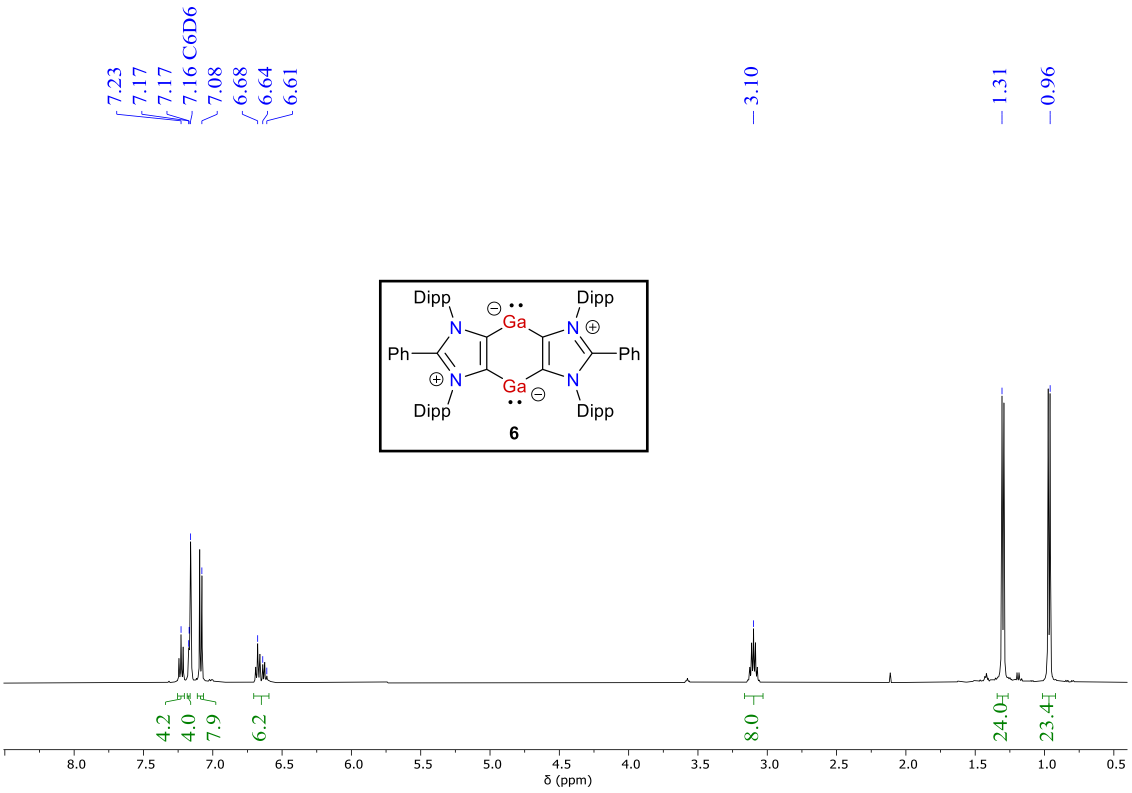


**Figure S11.** $^{1}H$ NMR (500 MHz, $C_6D_6$, 298 K) spectrum of **6**.

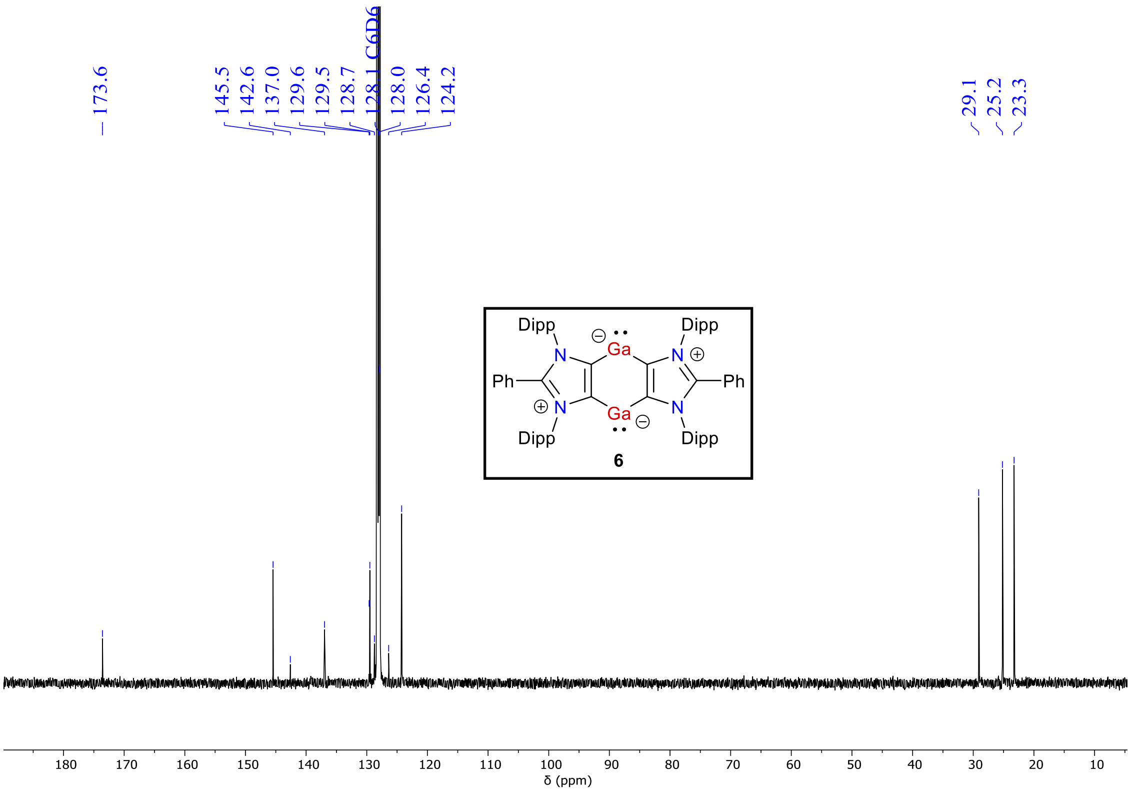


**Figure S12.** $^{13}C\{^{1}H\}$ NMR (126 MHz, $C_6D_6$, 298 K) spectrum of **6**.

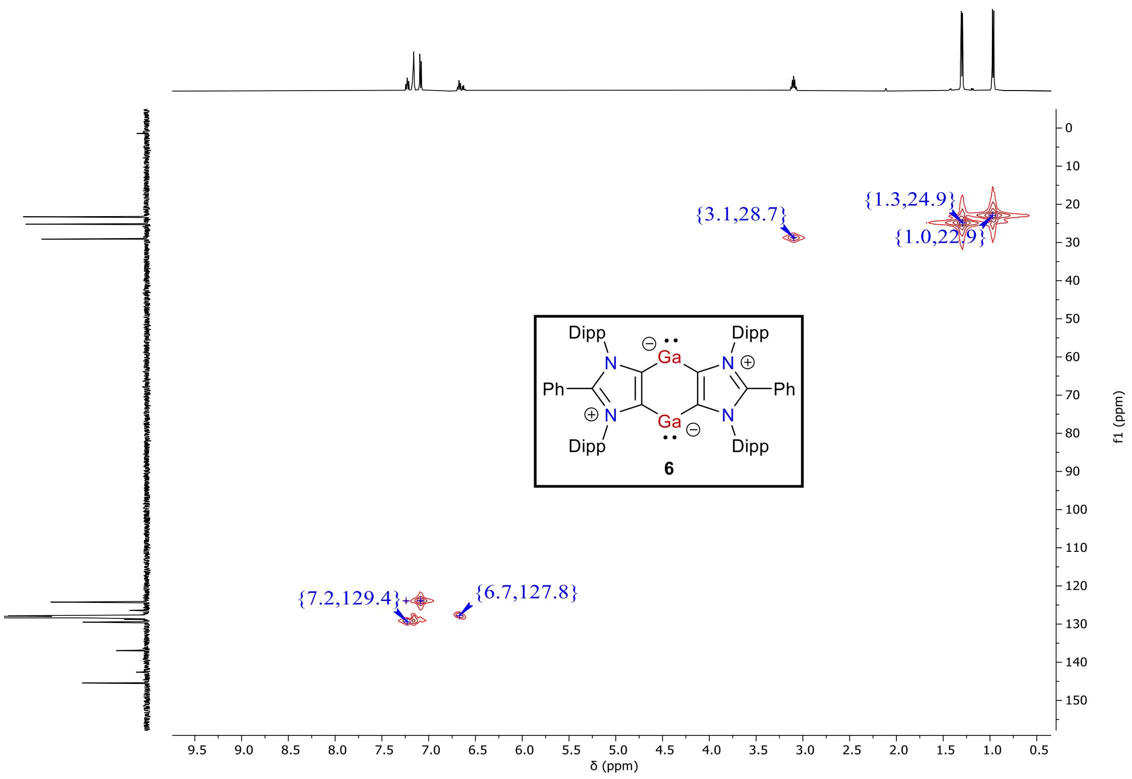


**Figure S13.** $^{1}H$-$^{13}C\{^{1}H\}$ HMQC (500/126 MHz, $C_6D_6$, 298 K) spectrum of **6**.

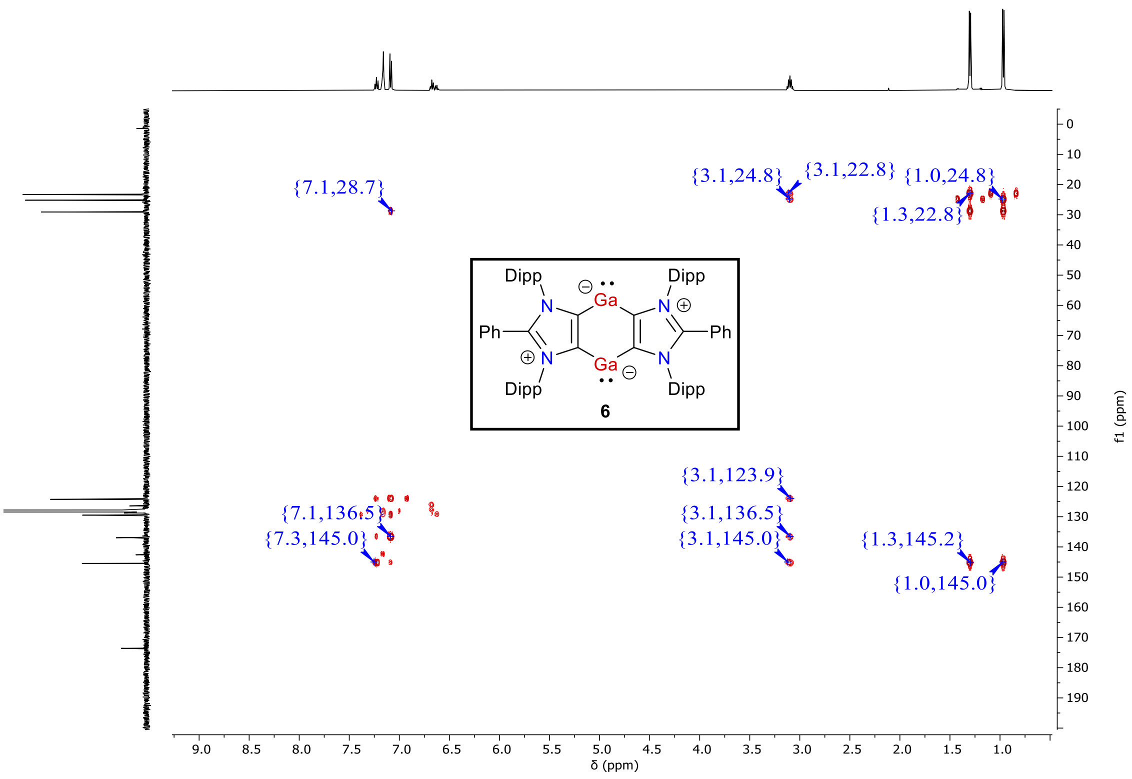


**Figure S14.** $^{1}H$-$^{13}C\{^{1}H\}$ HMBC (500/126 MHz, $C_6D_6$, 298 K) spectrum of **6**.

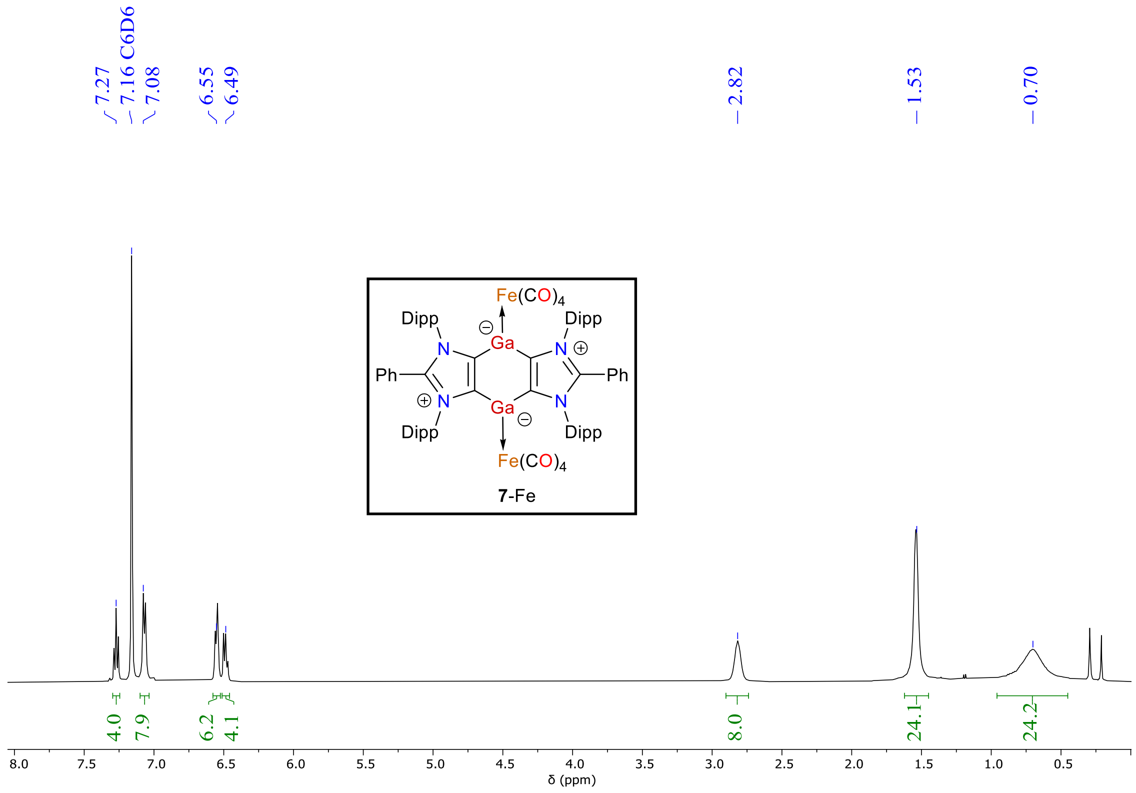


**Figure S15.** $^{1}$H NMR (500 MHz, $C_6D_6$, 298 K) spectrum of **7**.

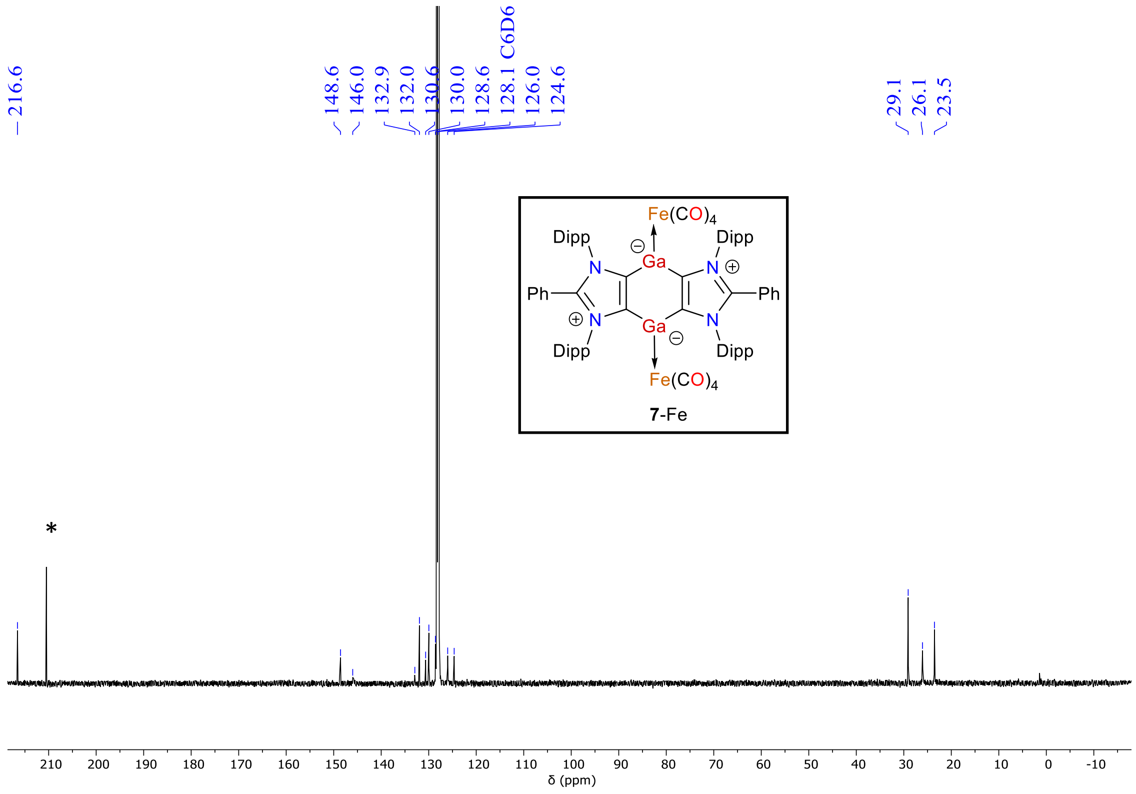


**Figure S16.** $^{13}$C{$^{1}$H} NMR (126 MHz, $C_6D_6$, 298 K) spectrum of **7** (*$Fe(CO)_5$).

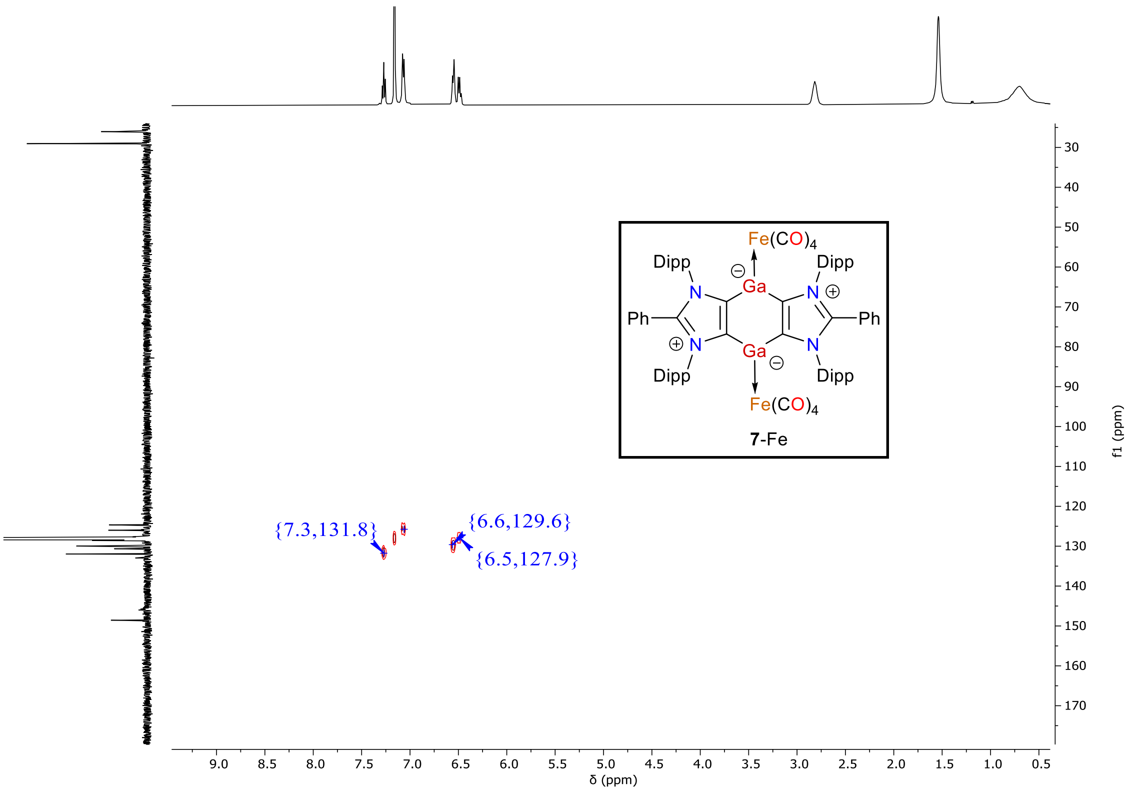


**Figure S17.** $^{1}H$-$^{13}C\{^{1}H\}$ HMQC (500/126 MHz, $C_6D_6$, 298 K) spectrum of **7**.

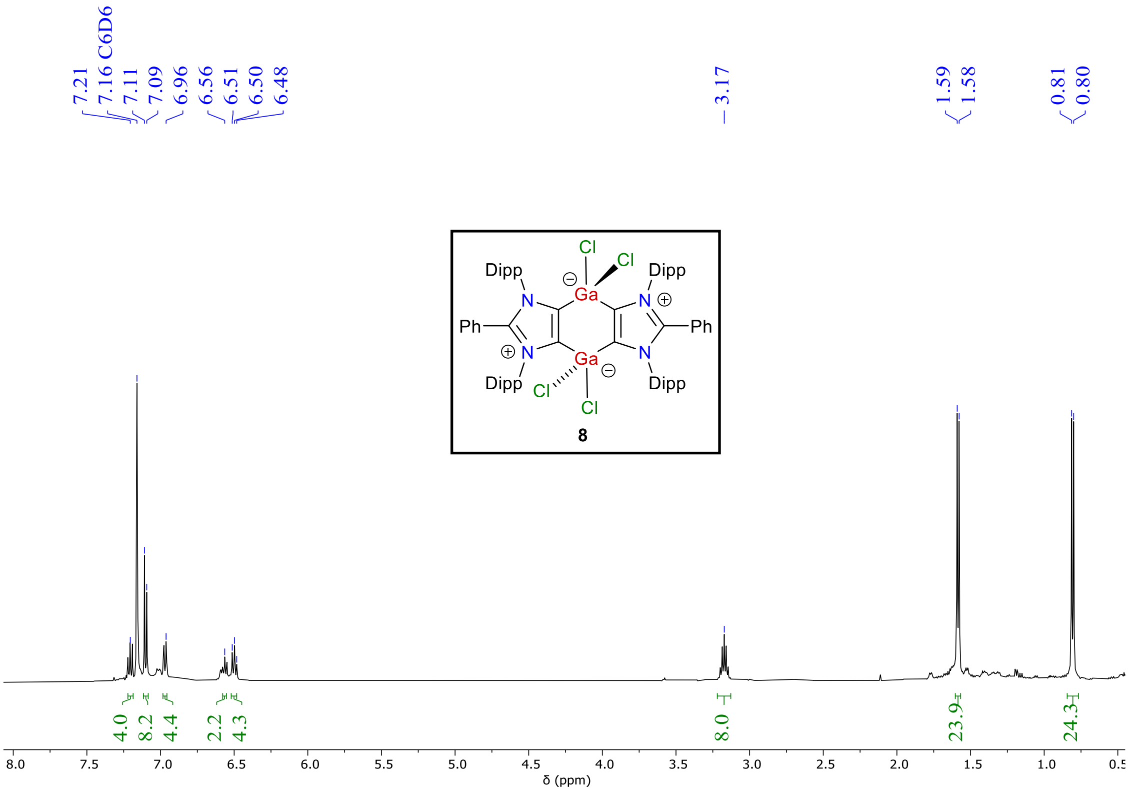


**Figure S18.** $^{1}H$ NMR (500 MHz, $C_6D_6$, 298 K) spectrum of **8**.

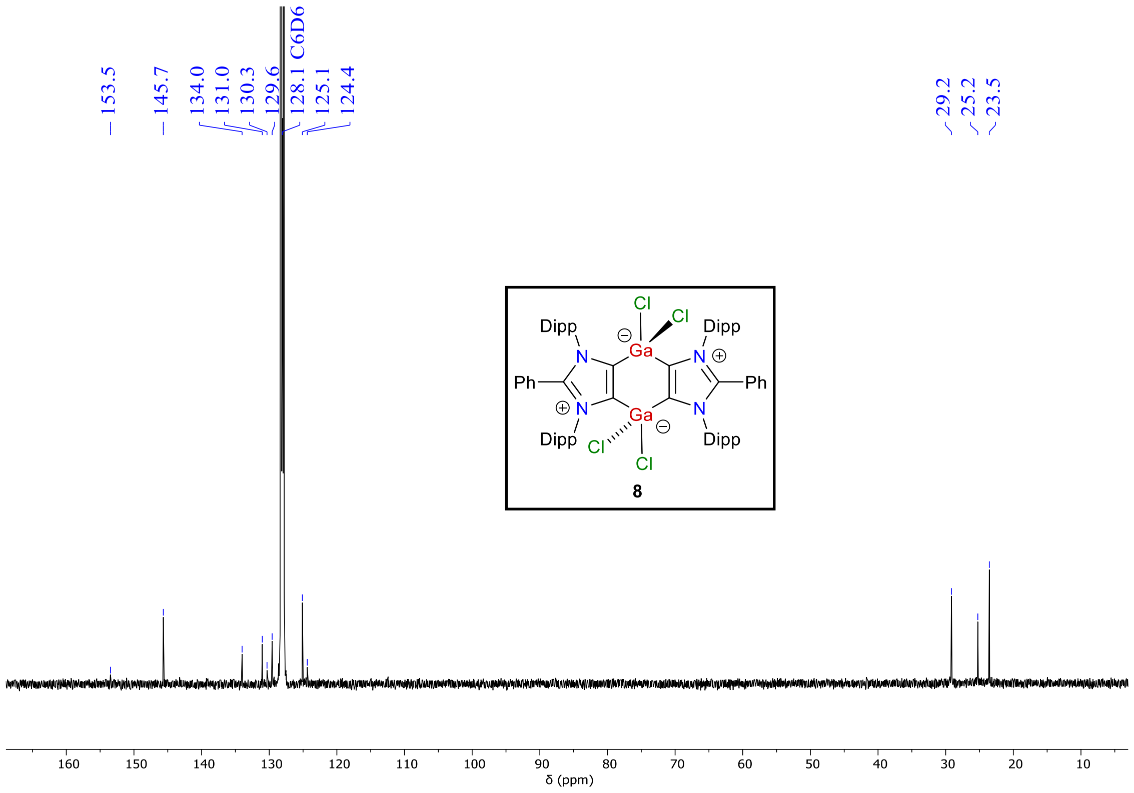


**Figure S19.** $^{13}C\{^{1}H\}$ NMR (126 MHz, $C_6D_6$, 298 K) spectrum of **8**.

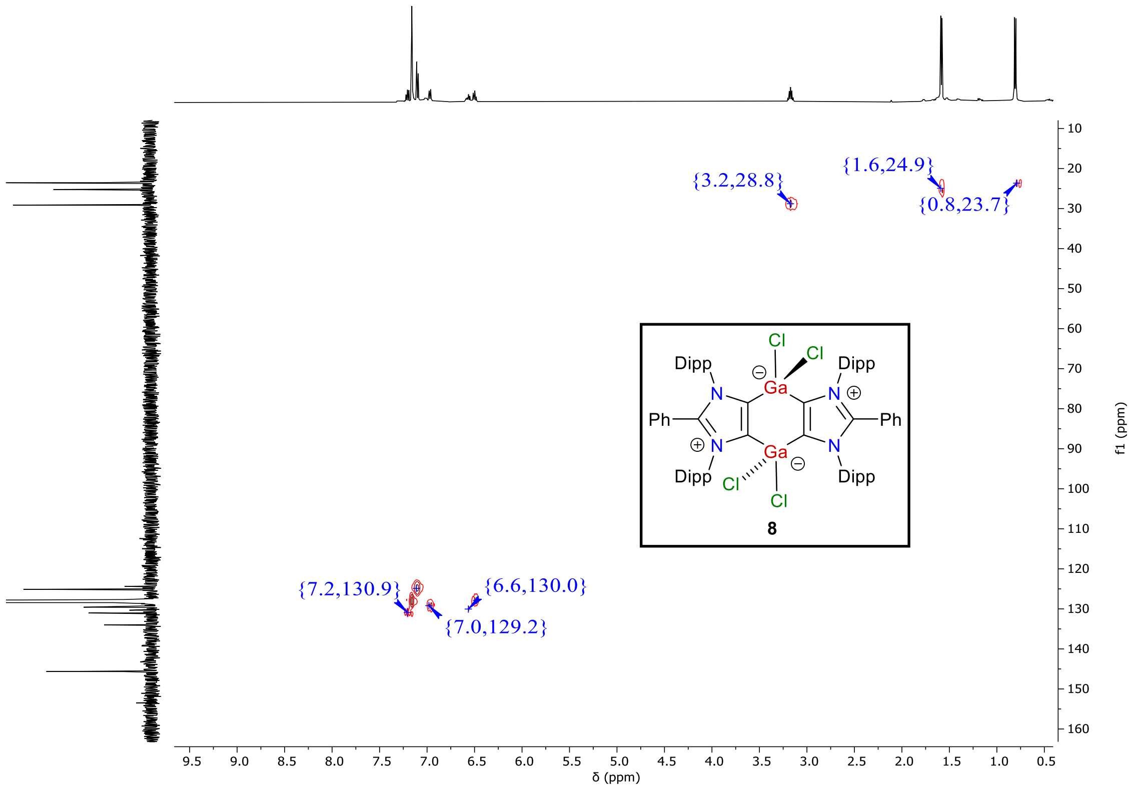


**Figure S20.** $^{1}H$-$^{13}C\{^{1}H\}$ HMQC (500/126 MHz, $C_6D_6$, 298 K) spectrum of **8**.

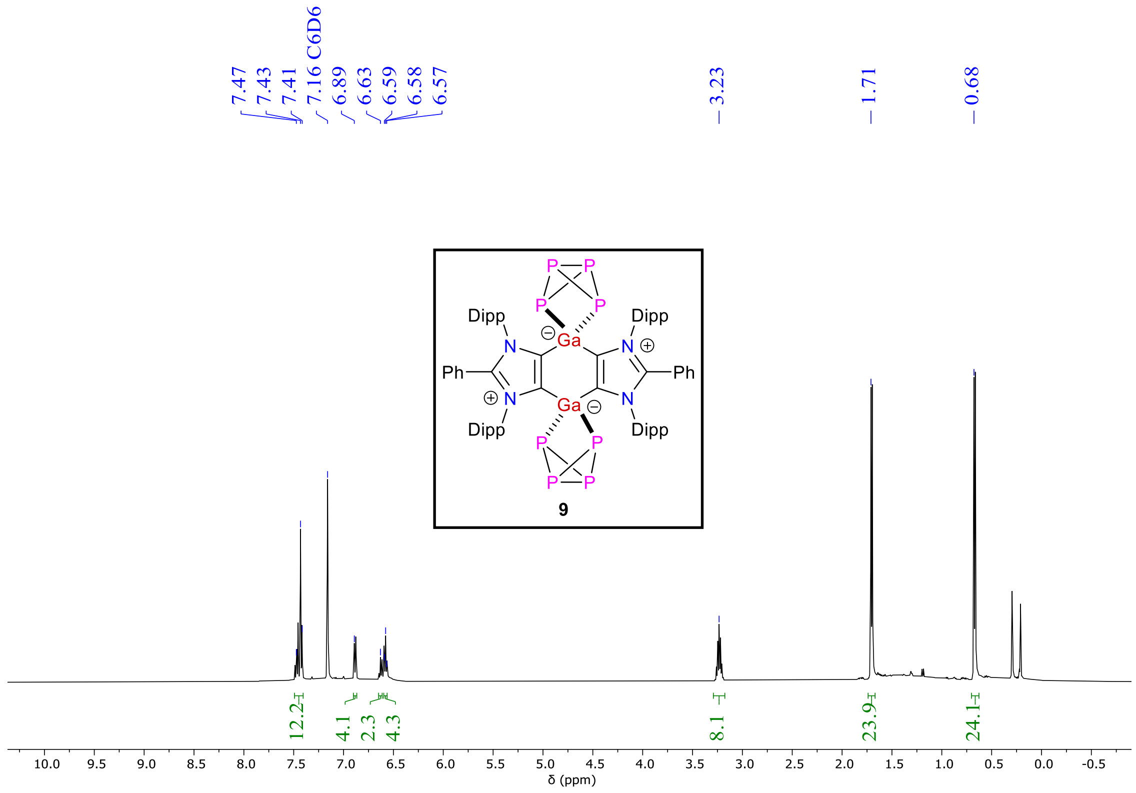


**Figure S21.** $^{1}H$ NMR (500 MHz, $C_6D_6$, 298 K) spectrum of **9**.

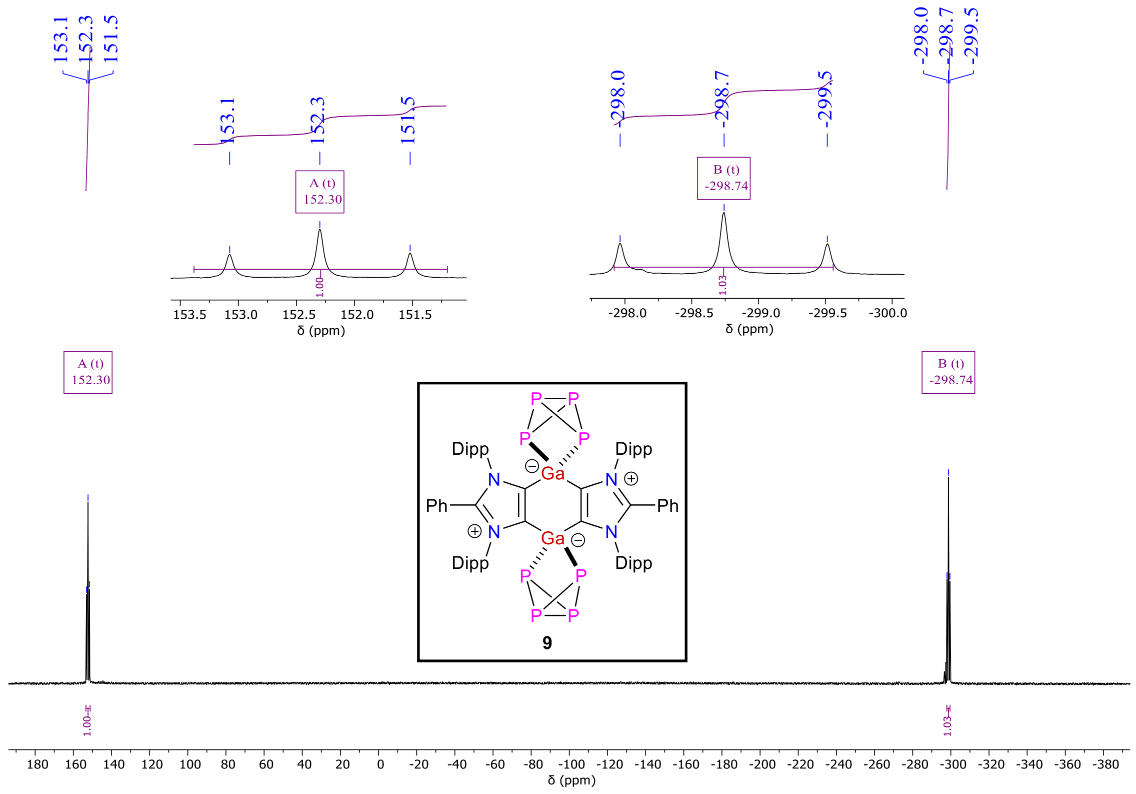


**Figure S22.** $^{1}H$ NMR (500 MHz, $C_6D_6$, 298 K) spectrum of **9**.

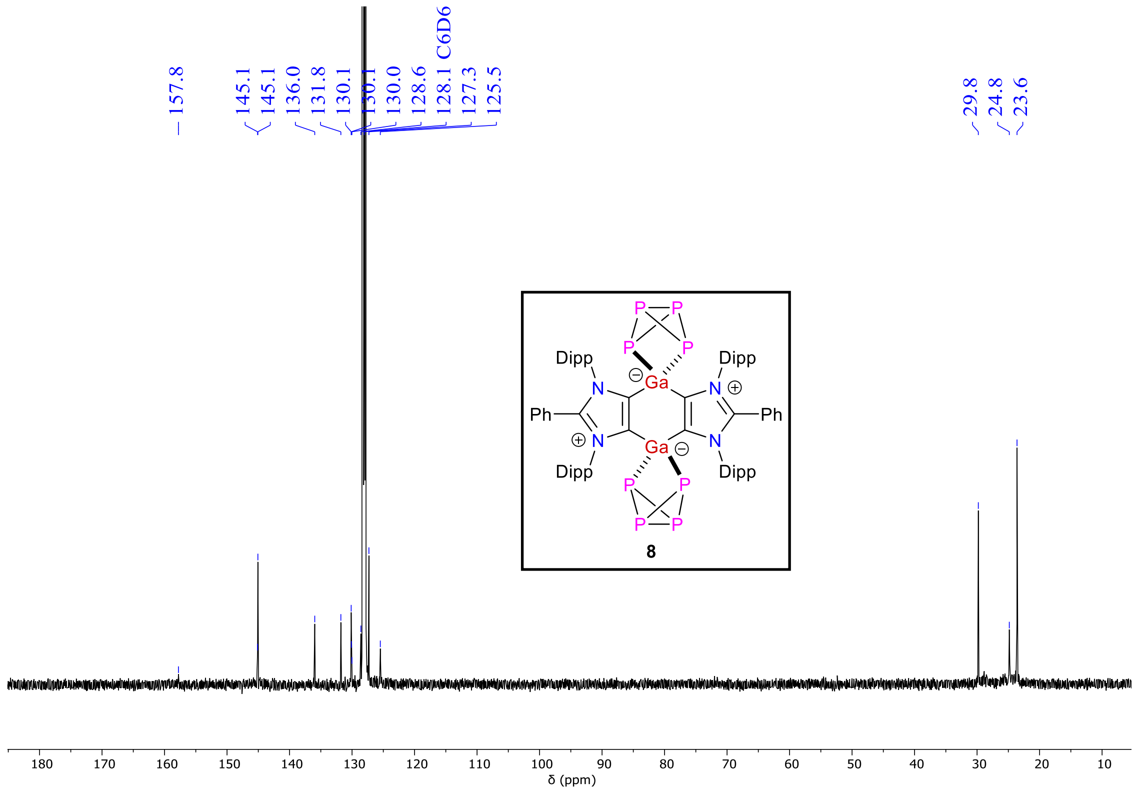


**Figure S23.** $^{13}C\{^1H\}$ NMR (126 MHz, $C_6D_6$, 298 K) spectrum of **9**.

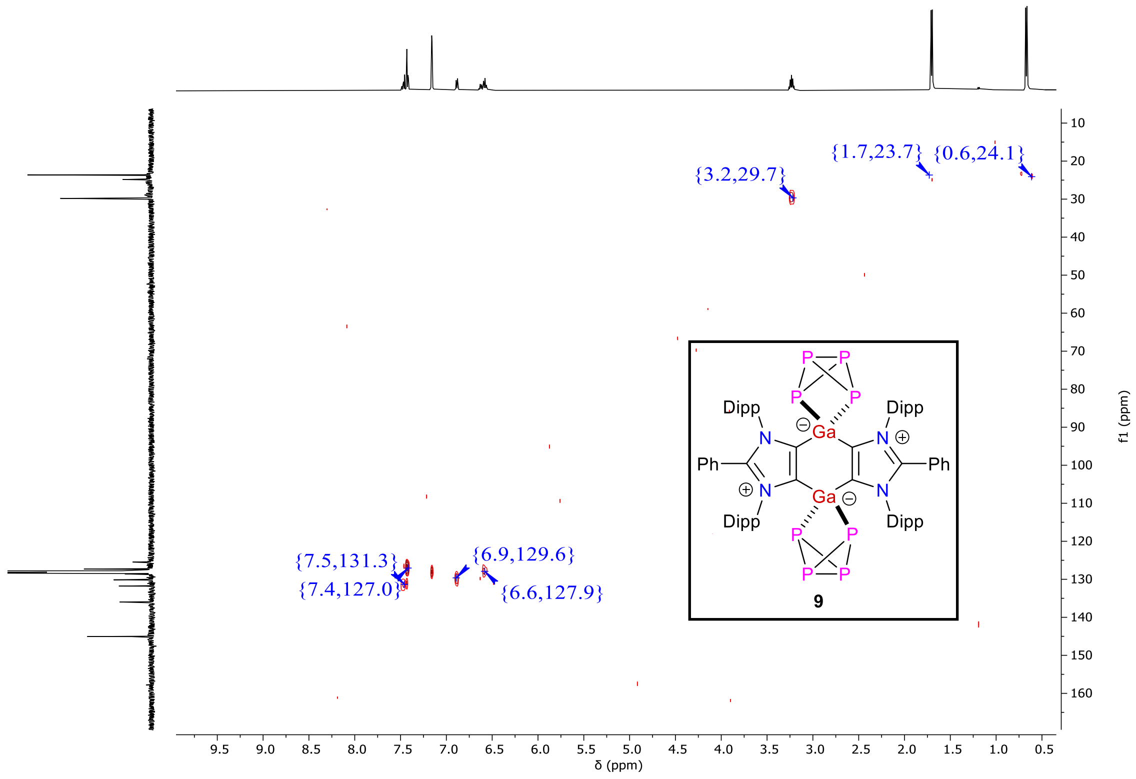


**Figure S24.** $^1H$-$^{13}C\{^1H\}$ HMQC (500/126 MHz, $C_6D_6$, 298 K) spectrum of **9**.

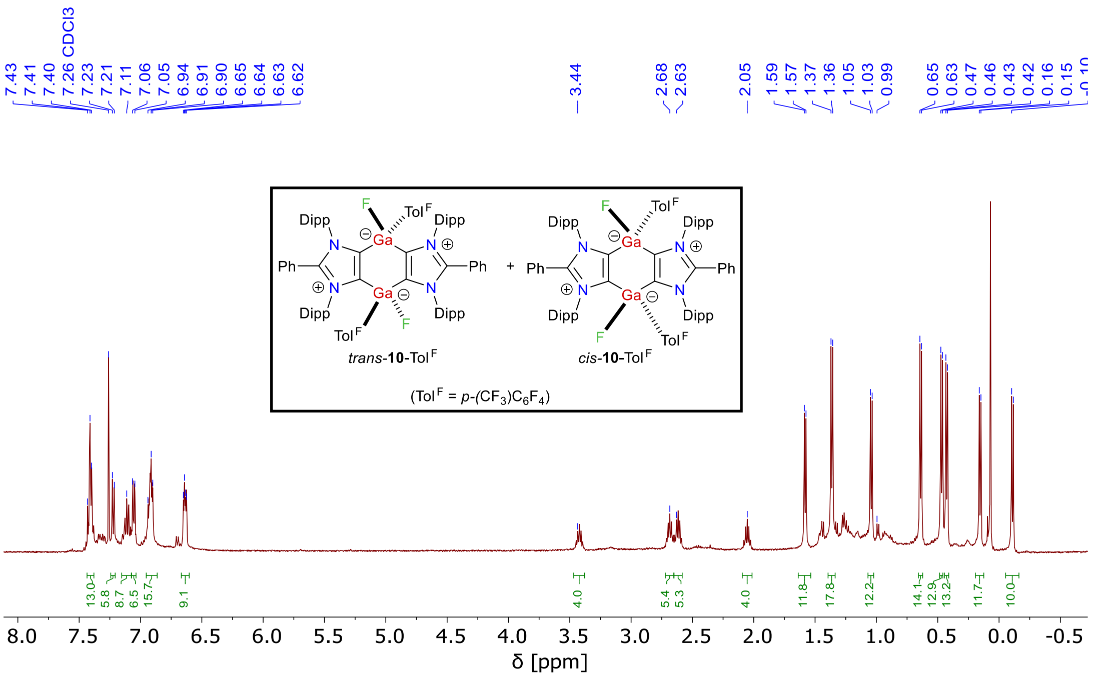

**Figure S25.** $^{1}H$ NMR (500 MHz, $CDCl_3$, 298 K) spectrum of a sample containing a mixture of *cis*-/*trans*-**10**-$Tol^F$.

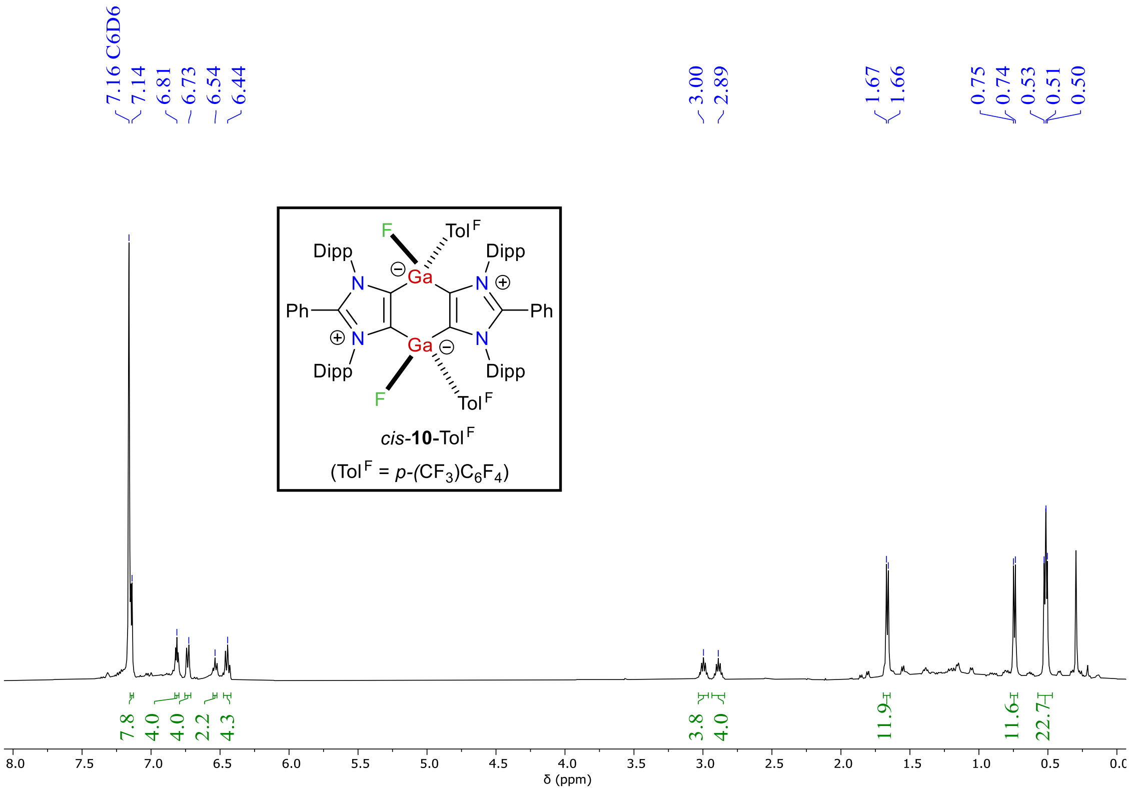

**Figure S26.** $^{1}H$ NMR (500 MHz, $C_6D_6$, 298 K) spectrum of *cis*-**10**-$Tol^F$.

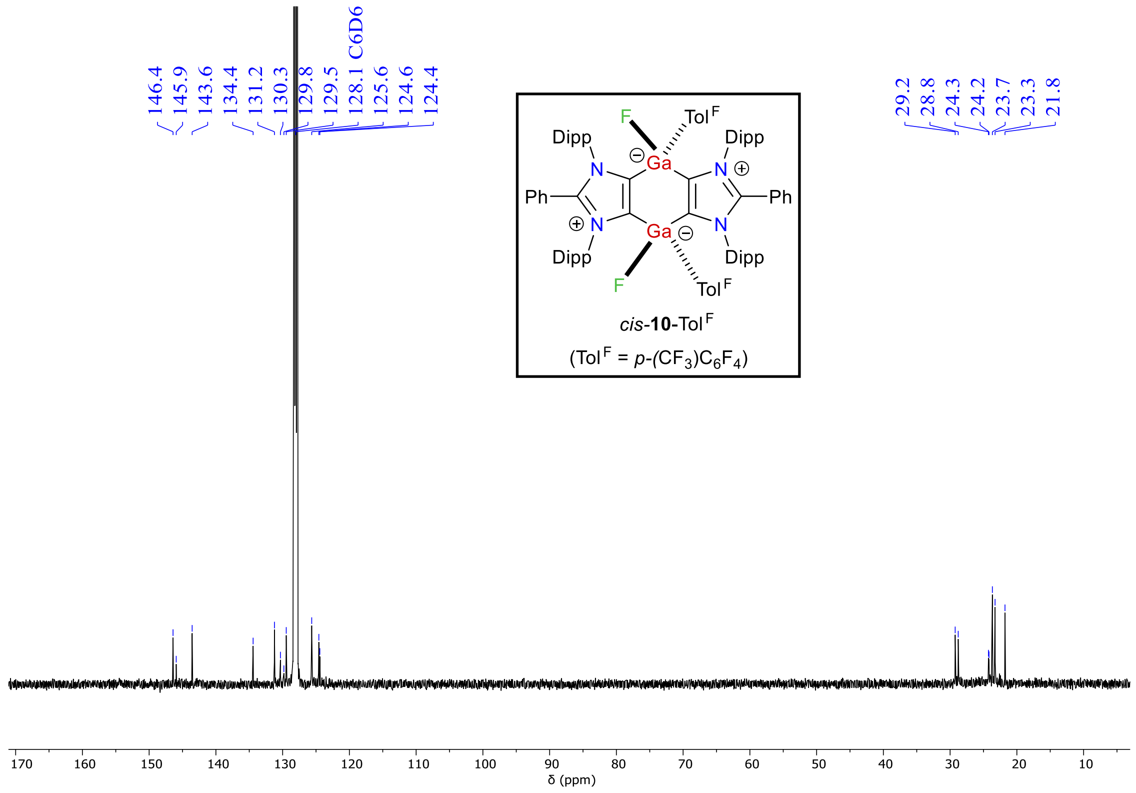


**Figure 27** $^{13}C\{^1H\}$ NMR (471 MHz, $C_6D_6$, 298 K) spectrum of *cis*-**10**-Tol$^F$.

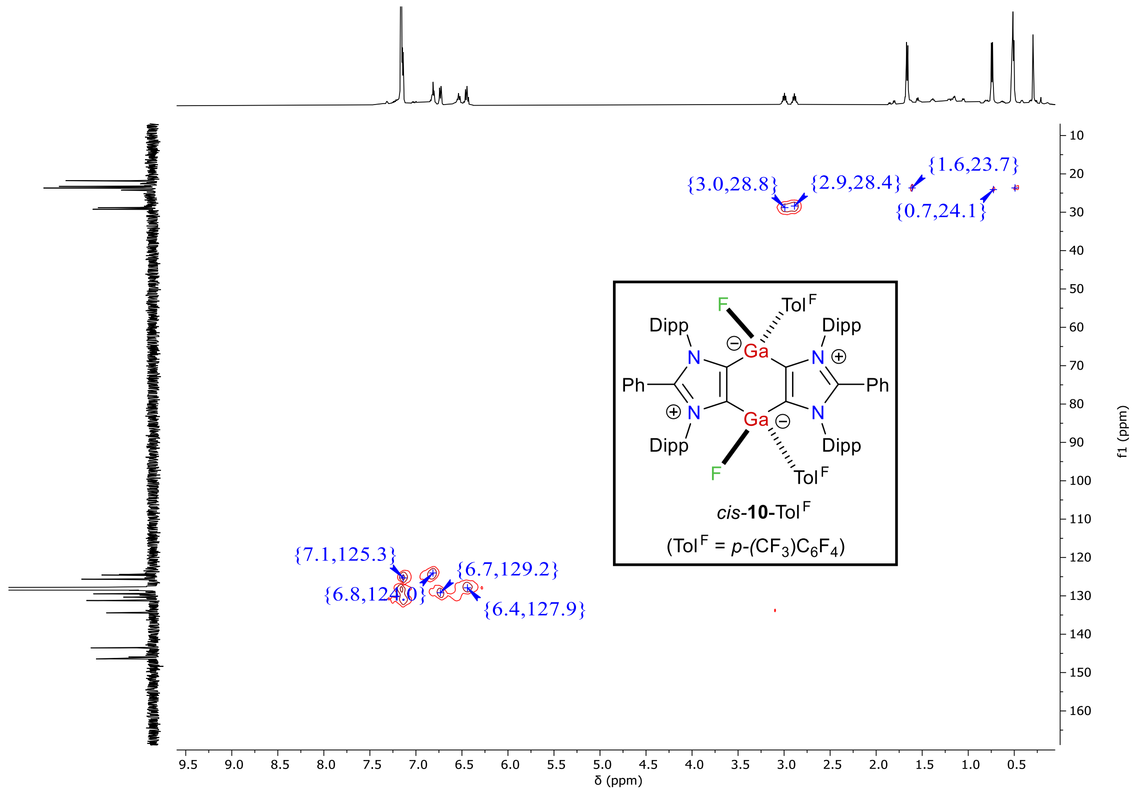


**Figure S28.** $^1H$-$^{13}C\{^1H\}$ HMQC (500/126 MHz, $C_6D_6$, 298 K) spectrum of *cis*-**10**-Tol$^F$.

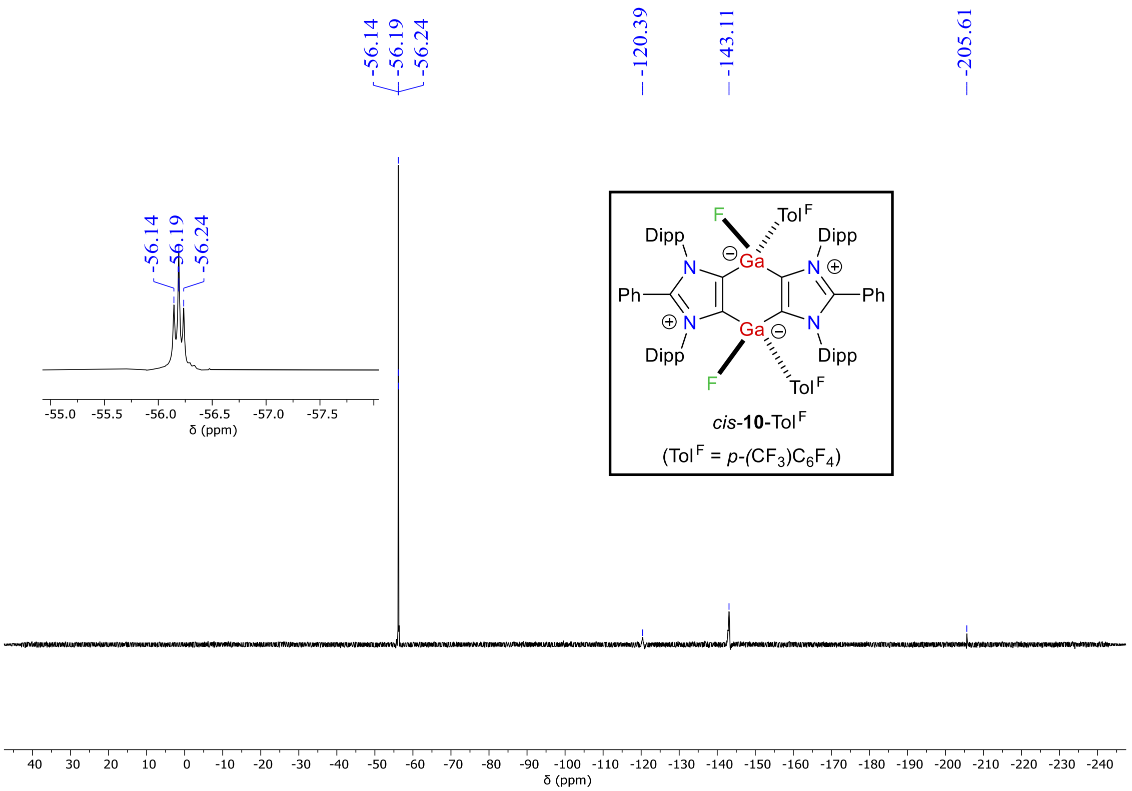


**Figure S29.** $^{19}F\{^{1}H\}$ NMR (126 MHz, $C_6D_6$, 298 K) spectrum of *cis*-**10**-$Tol^F$.

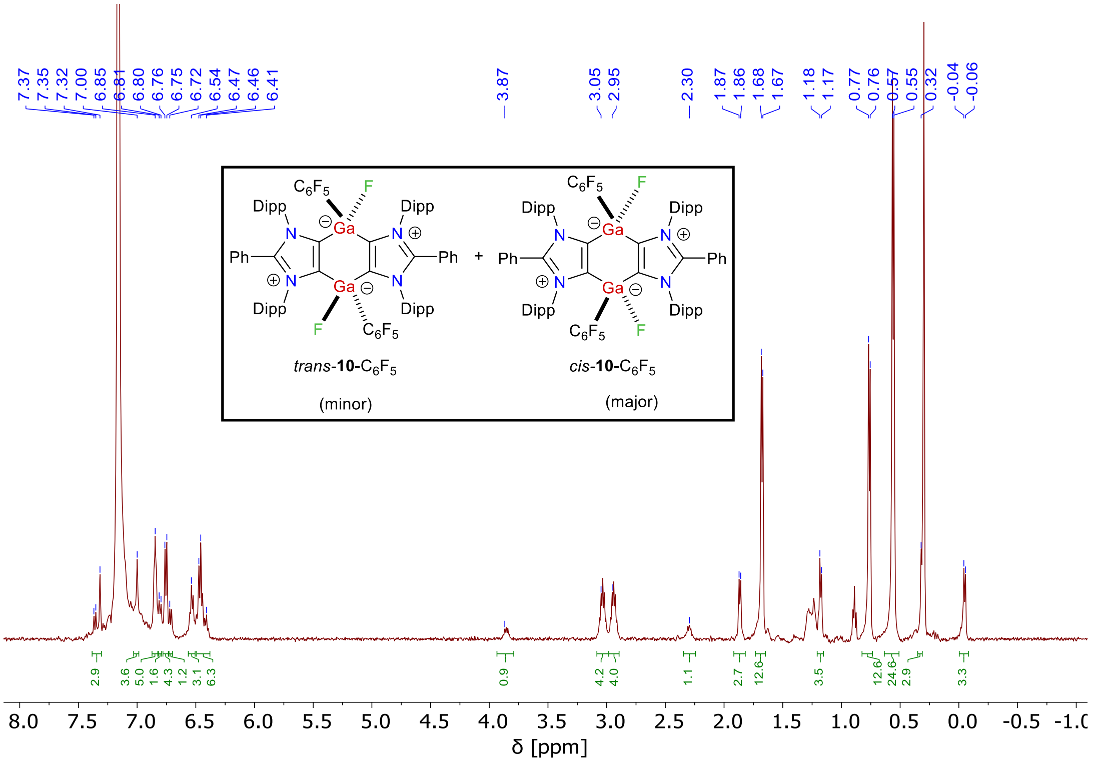


**Figure S30.** $^{1}H$ NMR (500 MHz, $C_6D_6$, 298 K) spectrum of a sample containing a mixture of *cis*-/*trans*-**10**-$C_6F_5$.

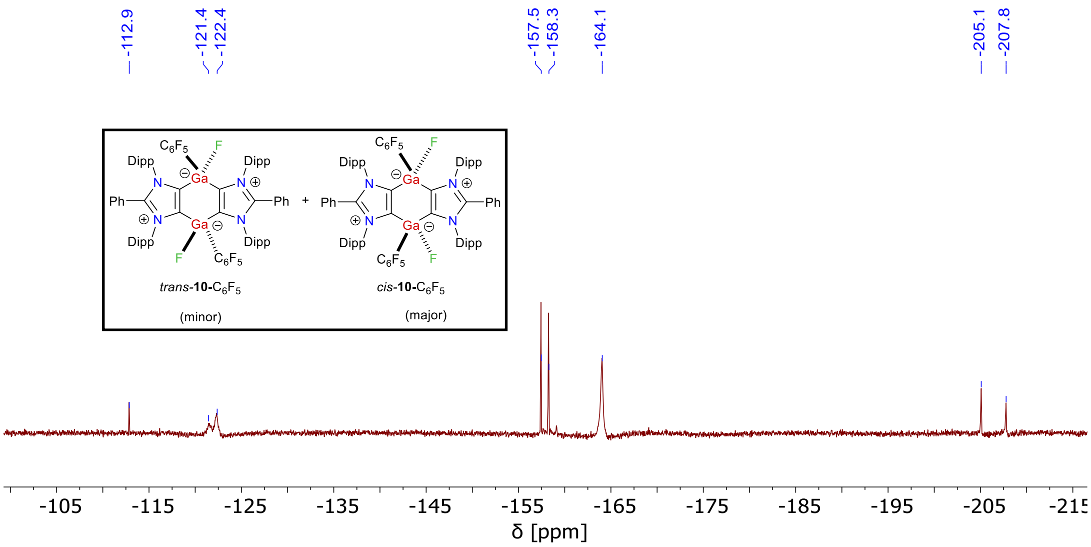


**Figure S31.** $^{19}F\{^{1}H\}$ NMR (126 MHz, $C_6D_6$, 298 K) spectrum of a sample containing a mixture of *cis*-/*trans*-**10**-$C_6F_5$ (signal at -112.9 ppm for PhF).

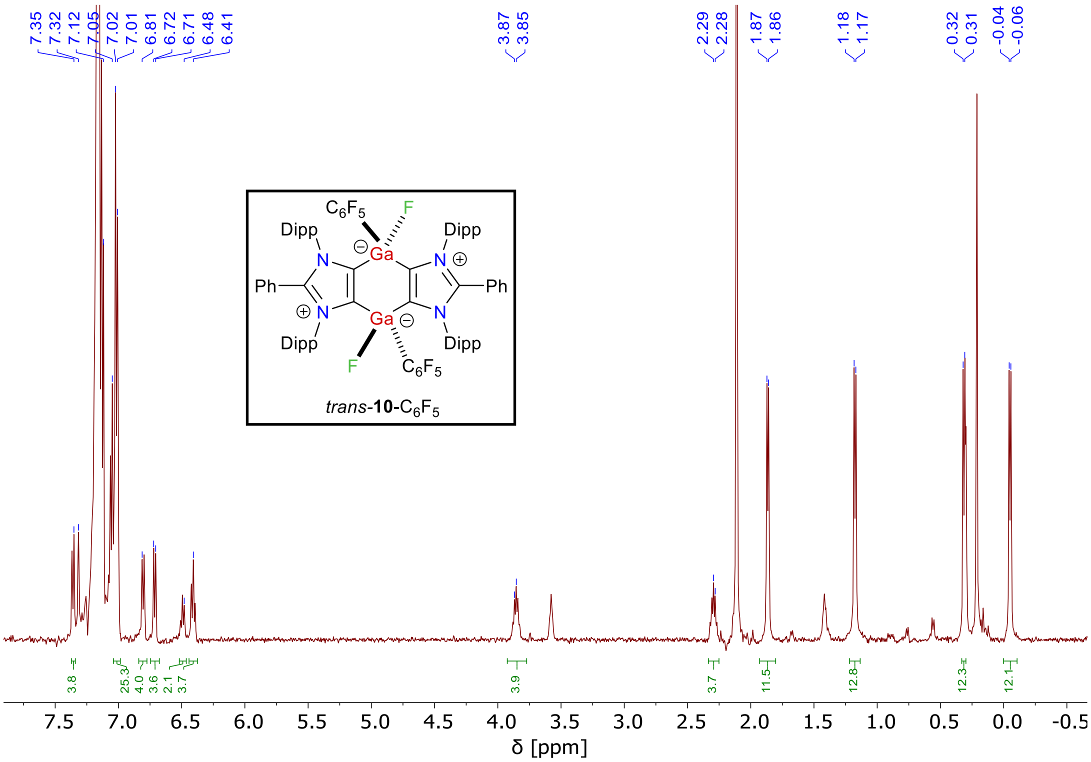


**Figure S32.** $^{1}H$ NMR (500 MHz, $C_6D_6$, 298 K) spectrum of *trans*-**10**-$C_6F_5$.

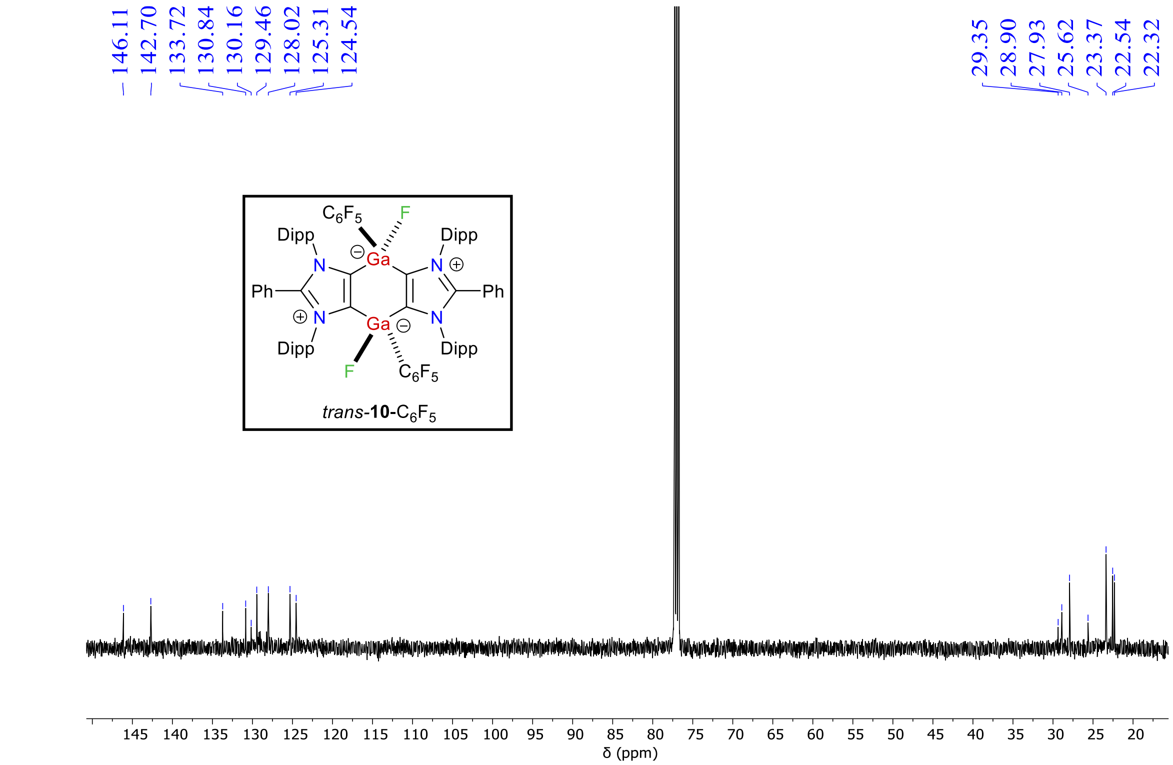


**Figure S33.** $^{13}C\{^1H\}$ NMR (471 MHz, $C_6D_6$, 298 K) spectrum of *trans*-**10**-$C_6F_5$.

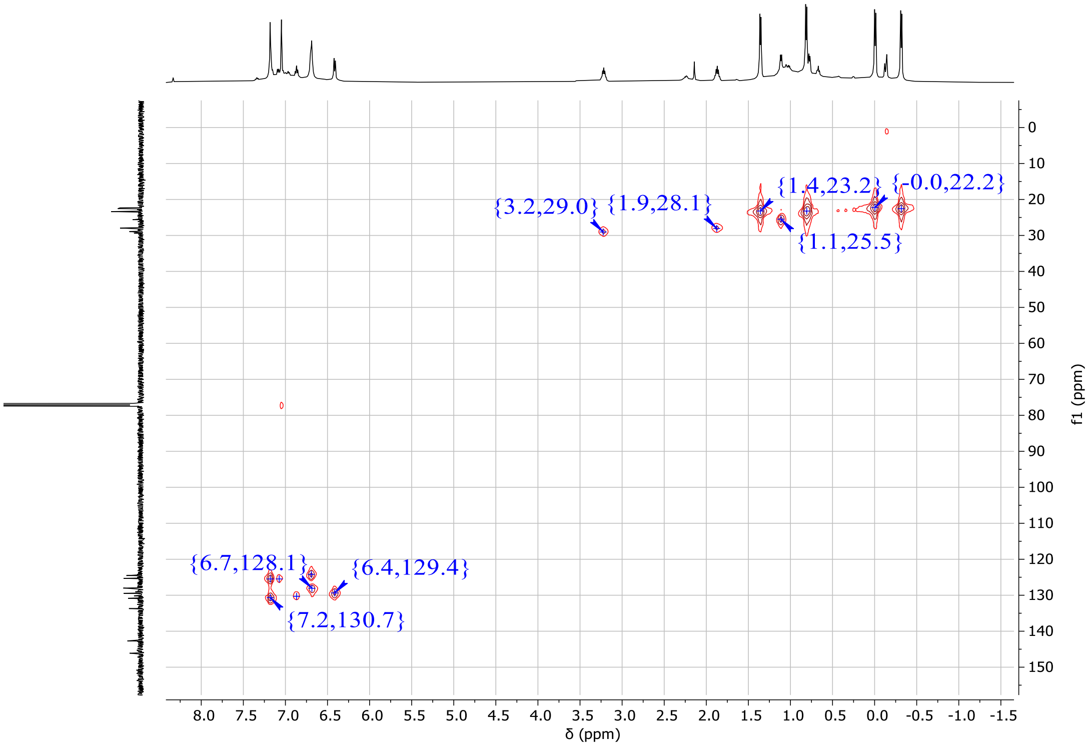


**Figure S34.** $^1H$-$^{13}C\{^1H\}$ HMQC (500/126 MHz, $C_6D_6$, 298 K) spectrum of *trans*-**10**-$C_6F_5$.

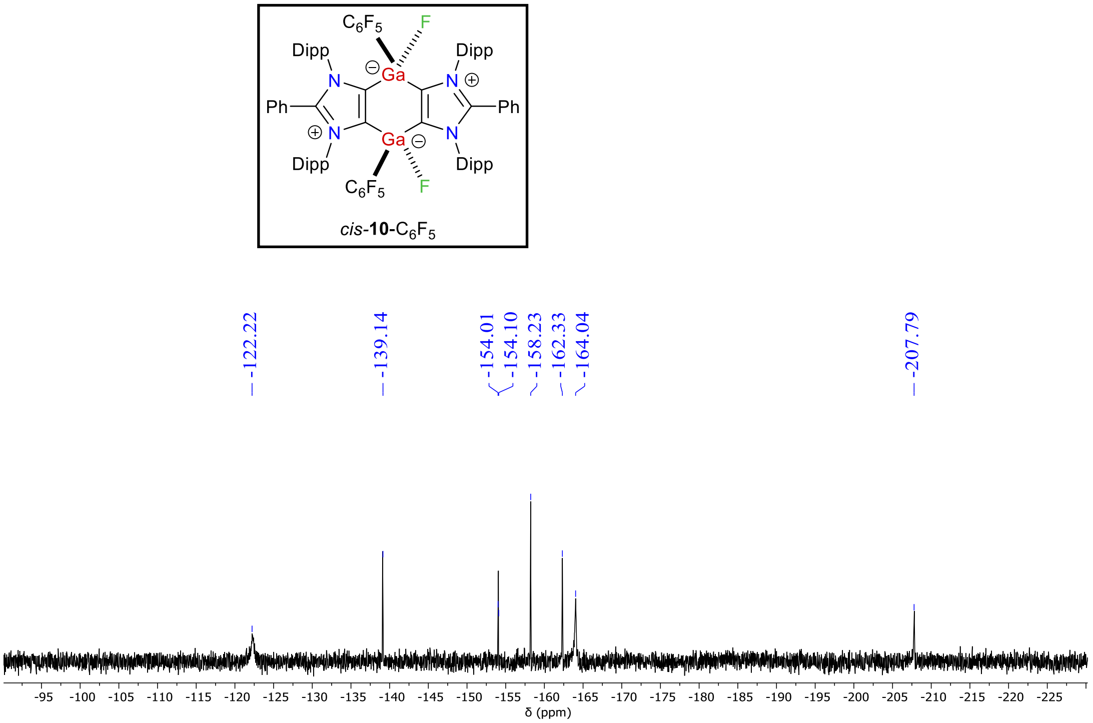


**Figure S35.** $^{19}F\{^{1}H\}$ NMR (126 MHz, $C_6D_6$, 298 K) spectrum of *cis*-**10**-$C_6F_5$.

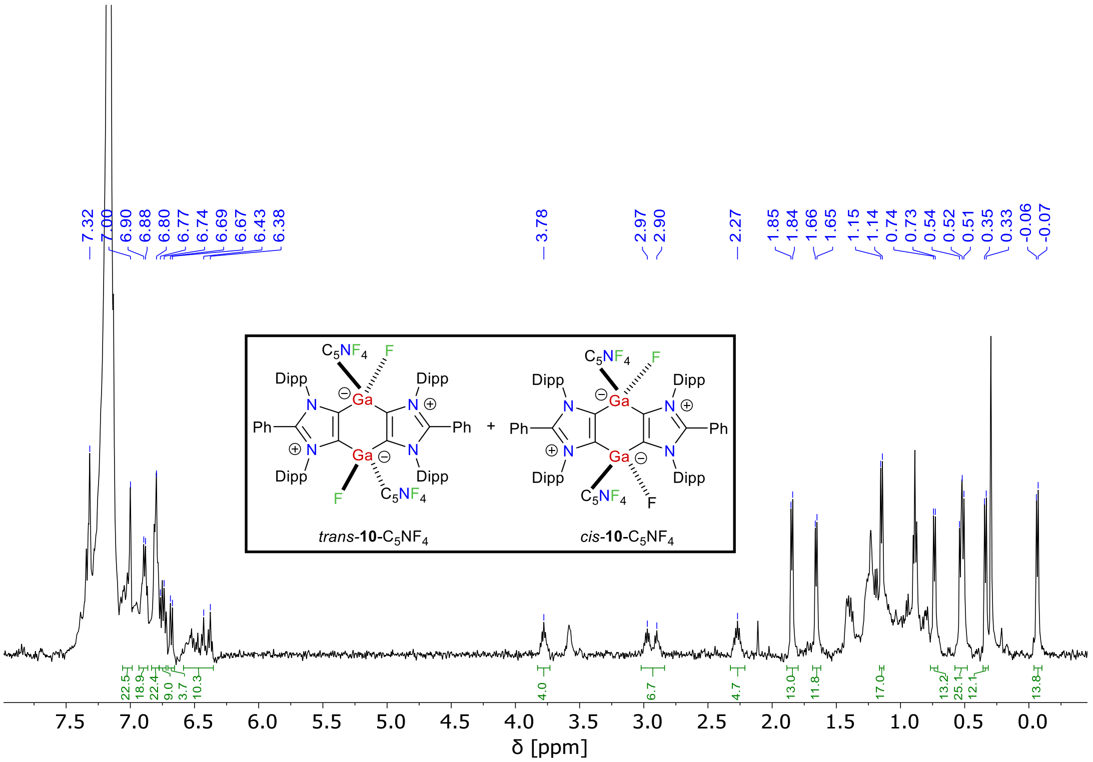


**Figure S36.** $^{1}H$ NMR (500 MHz, $C_6D_6$, 298 K) spectrum of a sample containing a mixture of *cis*-/*trans*-**10**-$C_5NF_4$.

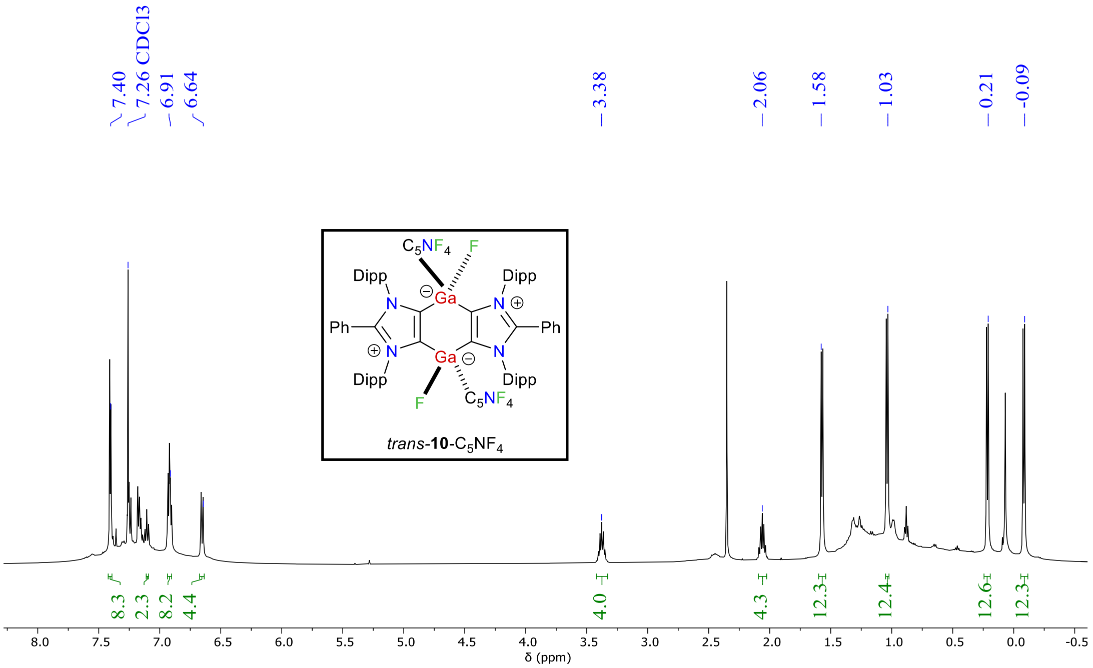

**Figure S37.** $^{1}$H NMR (500 MHz, $CDCl_3$, 298 K) spectrum of *trans*-**10**-$C_5NF_4$.

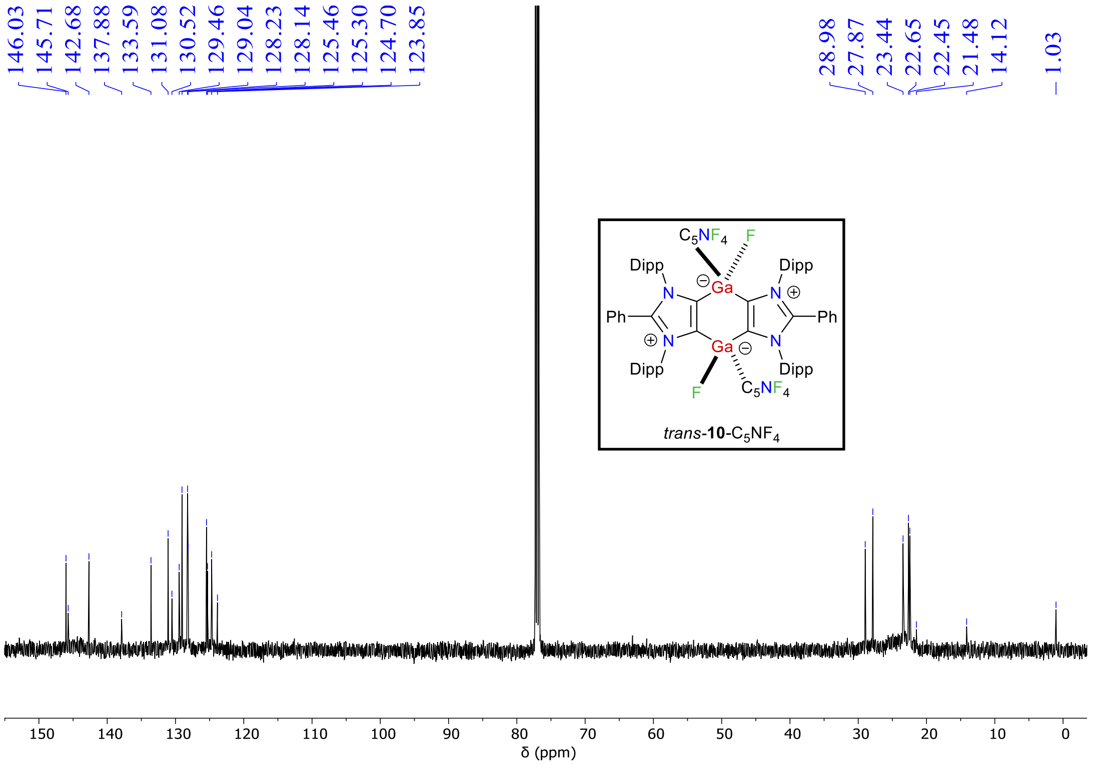

**Figure S38.** $^{13}C\{^{1}H\}$ NMR (471 MHz, $CDCl_3$, 298 K) spectrum of *trans*-**10**-$C_5NF_4$.

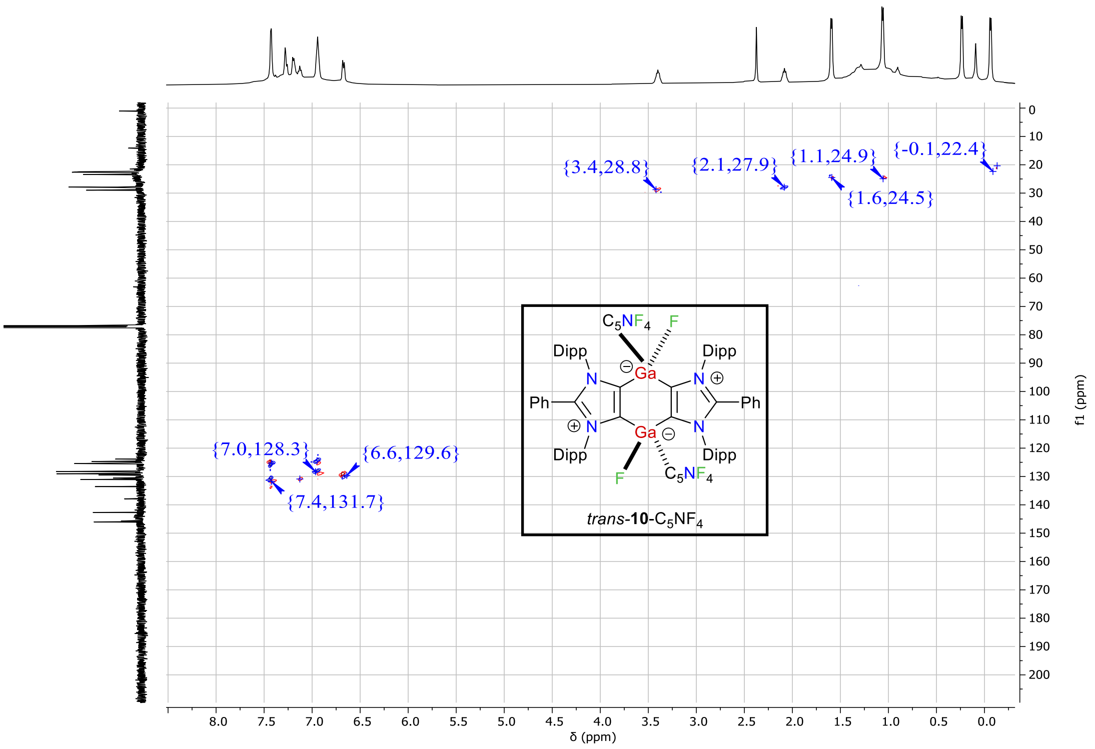


**Figure S39.** $^{1}H$-$^{13}C\{^{1}H\}$ HMQC (500/126 MHz, $CDCl_3$, 298 K) spectrum of *trans*-**10**-$C_5NF_4$.

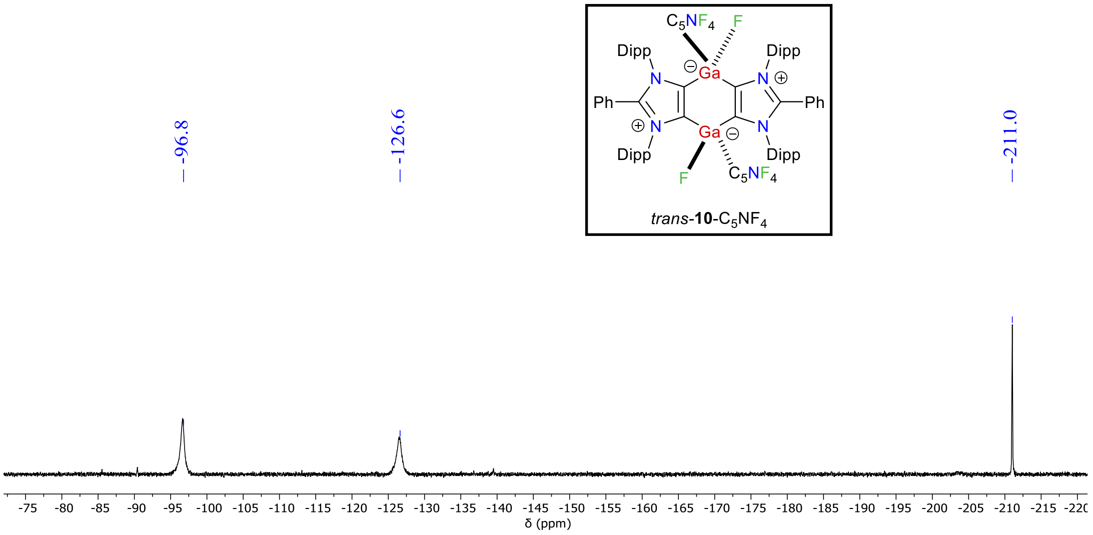


**Figure S40.** $^{19}F\{^{1}H\}$ NMR (126 MHz, $CDCl_3$, 298 K) spectrum of *trans*-**10**-$C_5NF_4$.

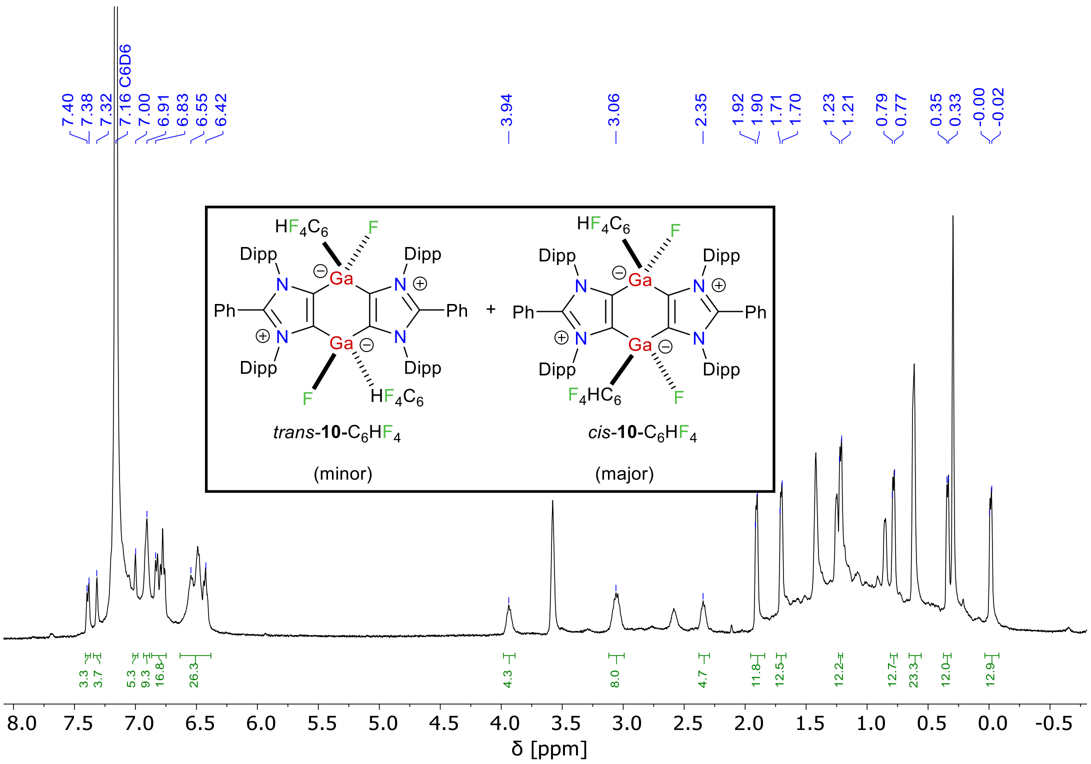


**Figure S41a.** $^{1}H$ NMR (500 MHz, $C_6D_6$, 298 K) spectrum of a sample containing a mixture of *cis*-/*trans*-**10**-$C_6HF_4$.

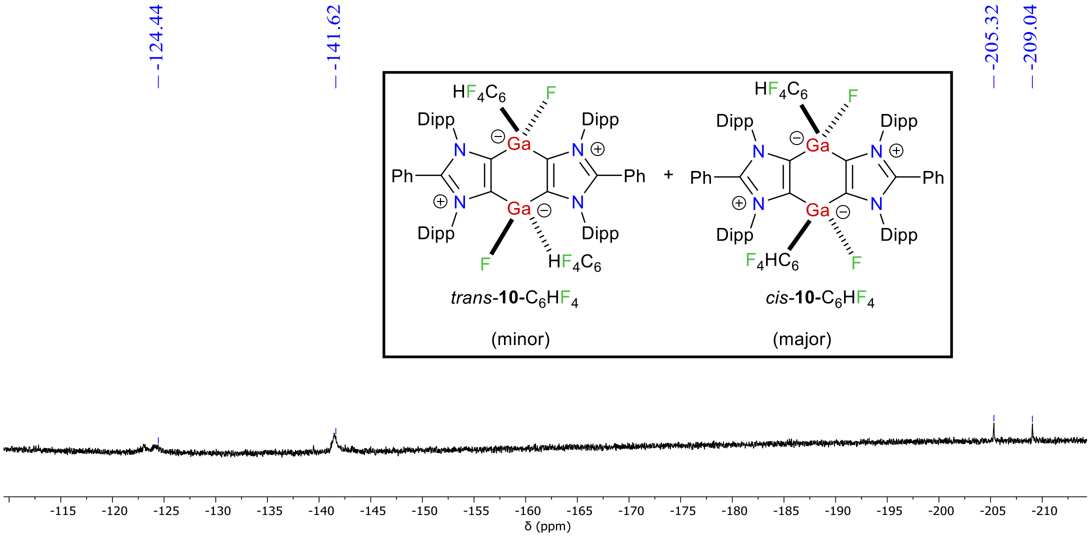


**Figure S41b.** $^{19}F\{^{1}H\}$ NMR (126 MHz, $C_6D_6$, 298 K) spectrum of *cis*-/*trans*-**10**-$C_6HF_4$.

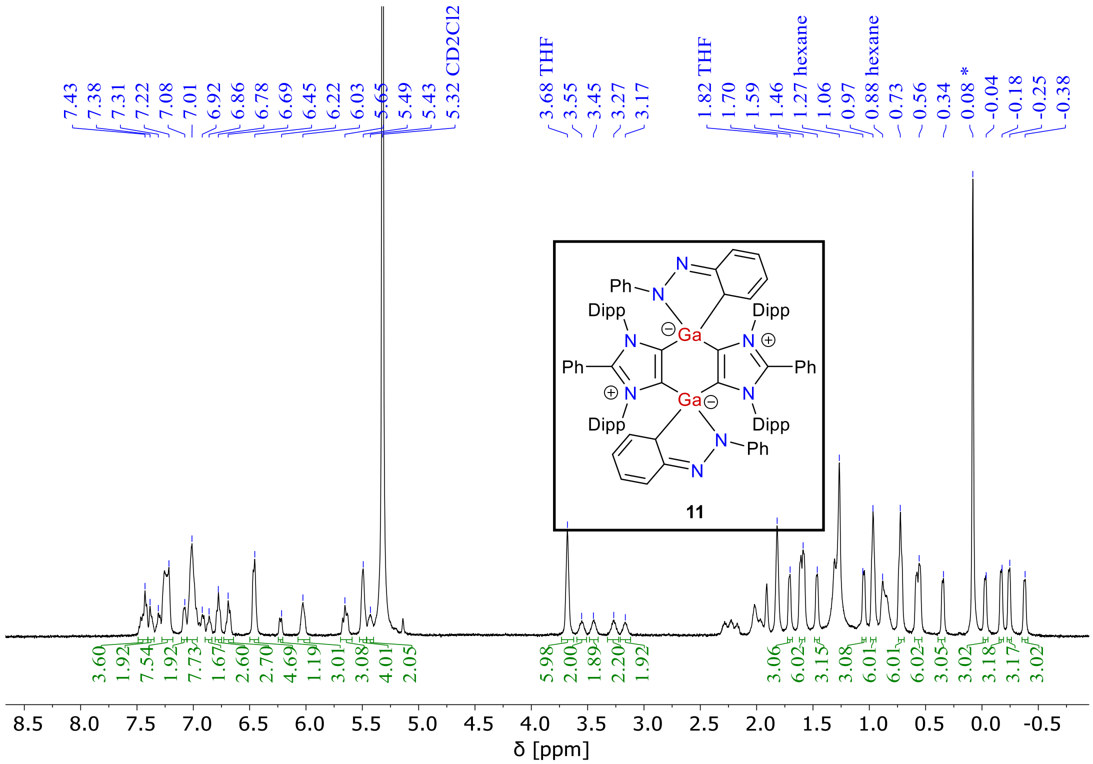

**Figure S42.** $^{1}H$ NMR (500 MHz, $CD_2Cl_2$, 298 K) spectrum of **11**.

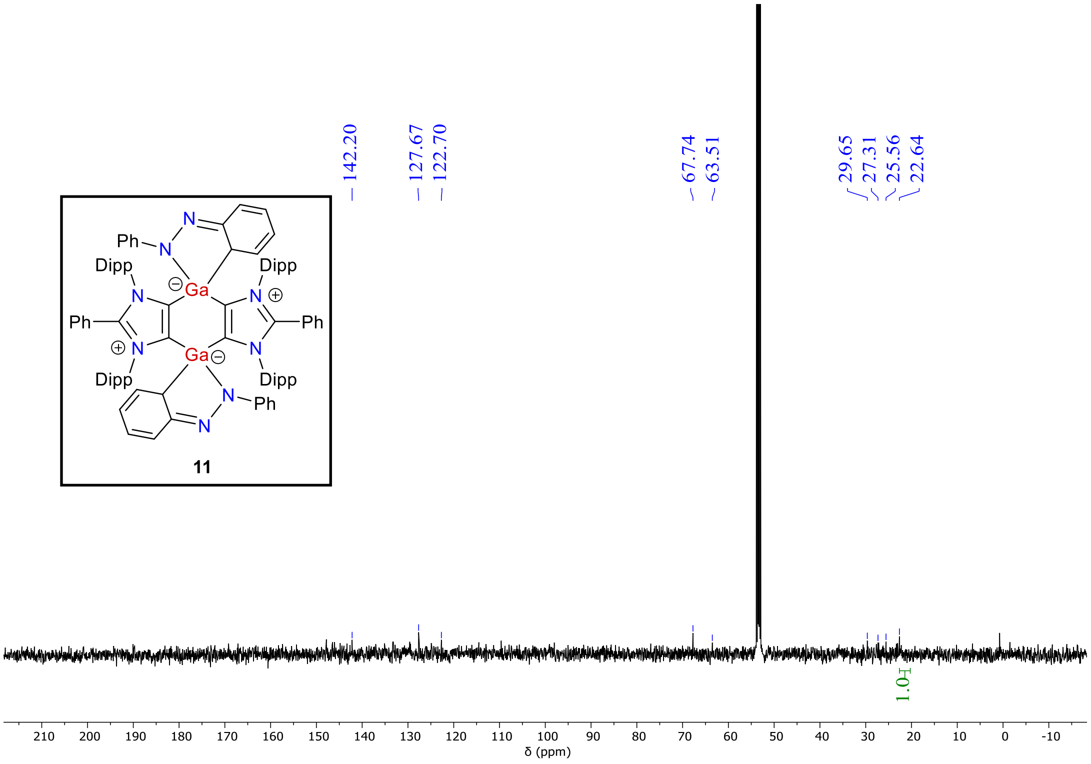

**Figure S43.** $^{13}C\{^{1}H\}$ NMR (471 MHz, $CD_2Cl_2$, 298 K) spectrum of **11** (almost insoluble).

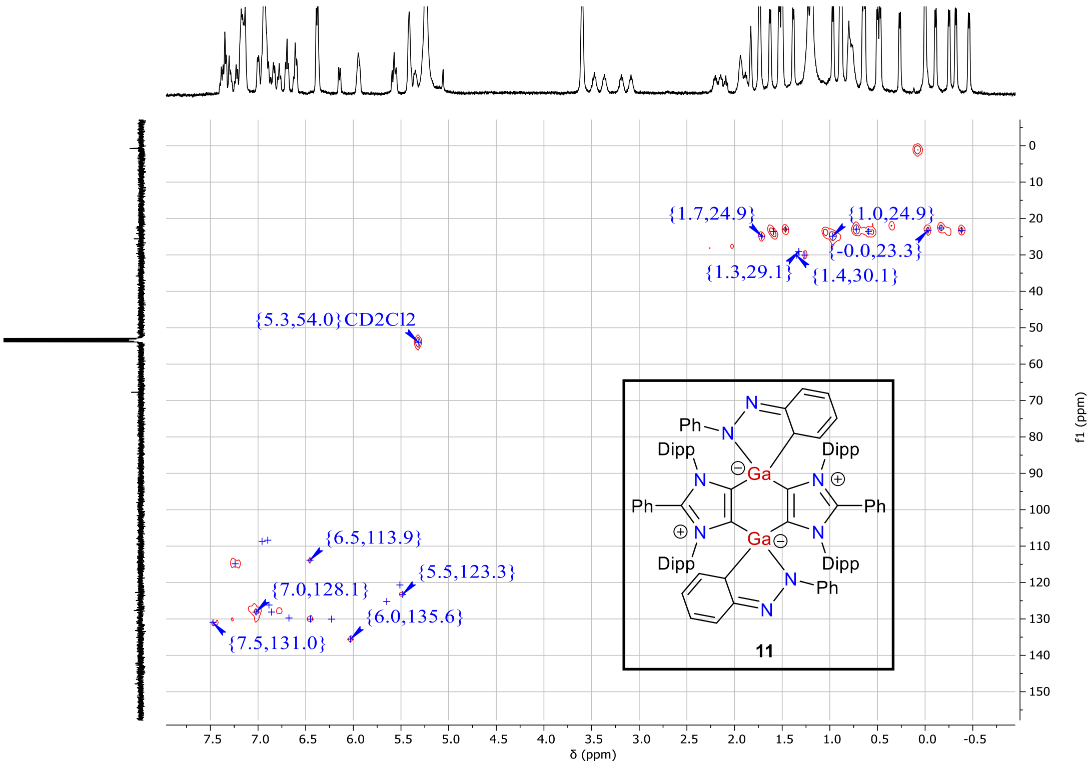


**Figure S44.** $^{1}H$-$^{13}C\{^{1}H\}$ HMQC (500/126 MHz, $CD_2Cl_2$, 298 K) (full) spectrum of **11**.

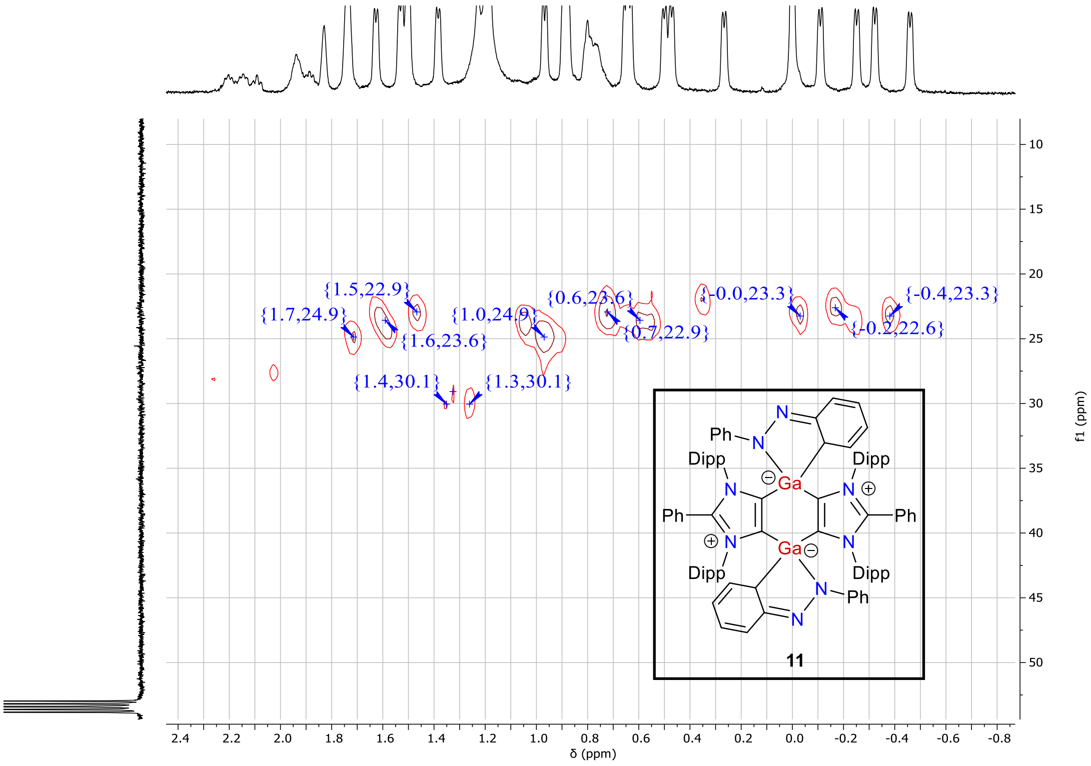


**Figure S45.** $^{1}H$-$^{13}C\{^{1}H\}$ HMQC (500/126 MHz, $CD_2Cl_2$, 298 K) (aliphatic region) spectrum of **11**.

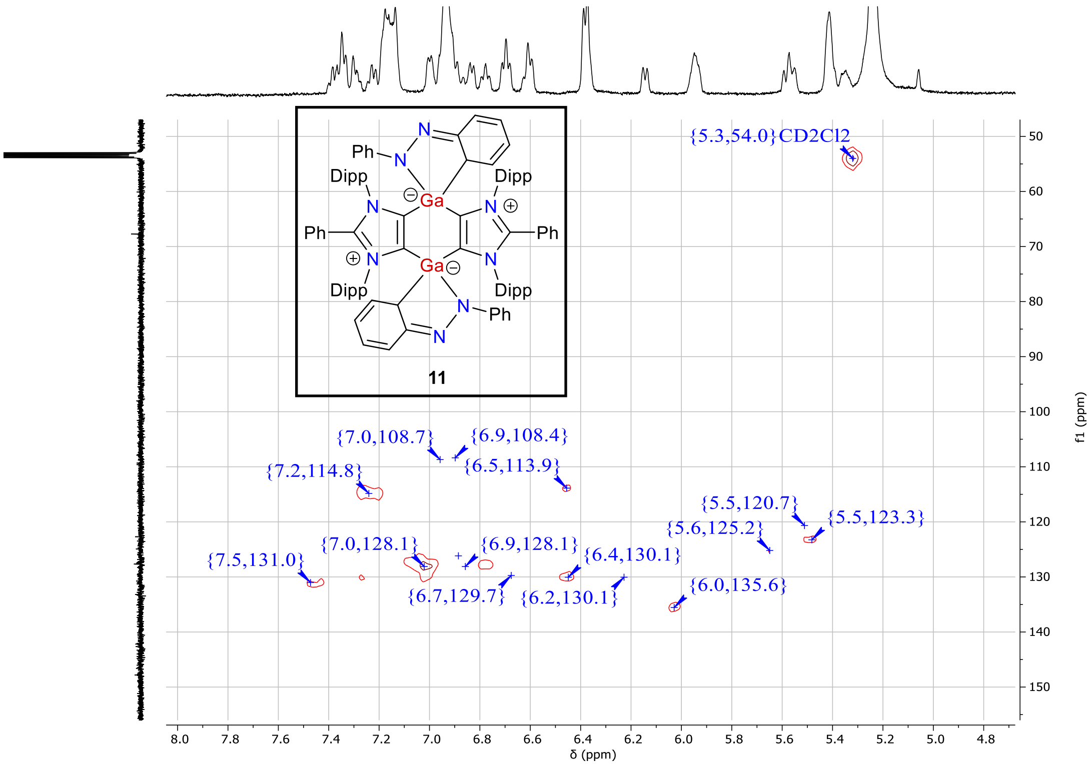


**Figure S46.** $^{1}H$ -$^{13}C\{^{1}H\}$ HMQC (500/126 MHz, $CD_2Cl_2$, 298 K) (aromatic region) spectrum of **11**.

## Plots of FT-IR Spectra

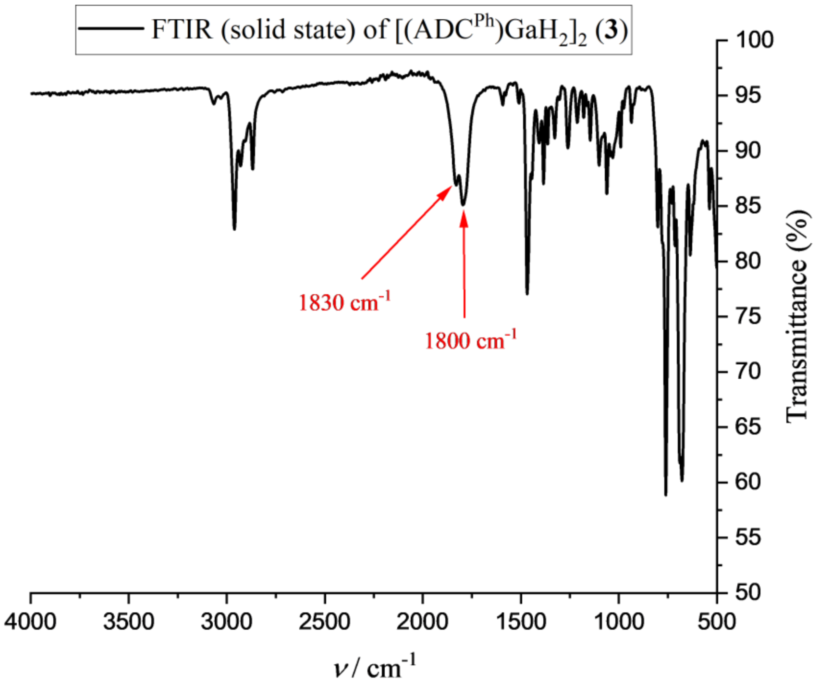


**Figure S47.** FT-IR spectrum of **3** in the solid state at 298 K.

# Plots of UV-Vis Spectra

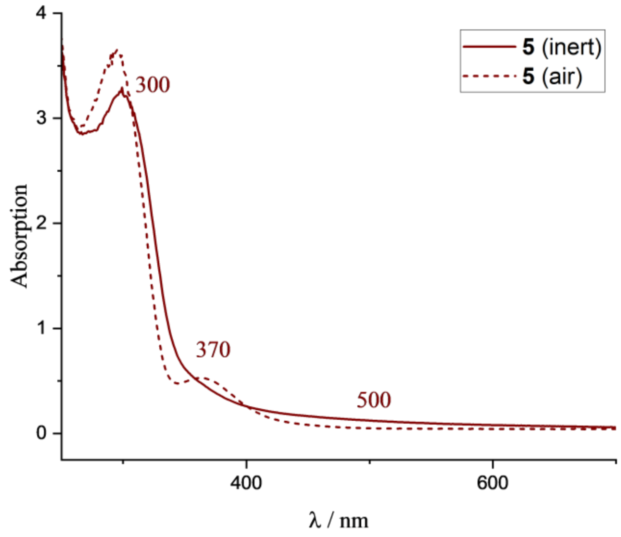


**Figure S48.** UV-Vis (THF, 2.5 x $10^{-4}$ M) spectrum of **5** under inert conditions (solid) and under air (dashed).

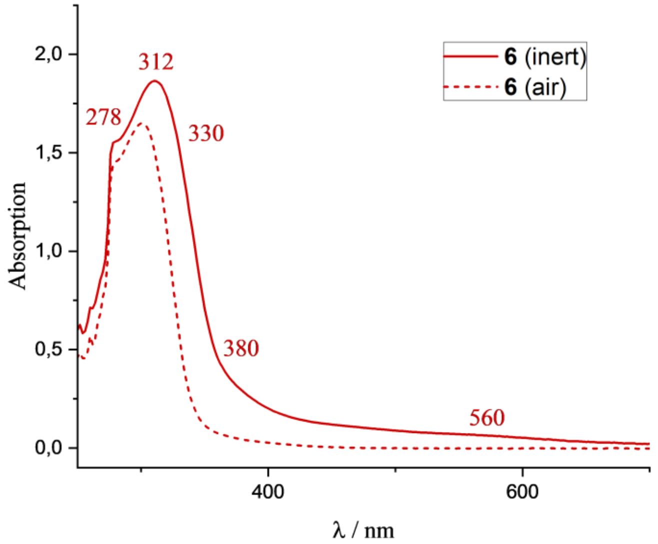


**Figure S49.** UV-Vis ($C_6H_6$, 5 x $10^{-4}$ M) spectrum of **6** under inert conditions (solid) and under air (dashed).

# Crystallographic Details

Single crystals were examined on a Rigaku Supernova diffractometer using Cu Kα (λ = 1.54184 Å) or Mo Kα (λ = 0.71073 Å) radiation. Using Olex2,[5] the structures were solved with the ShelXT[6] (**3**, **4**, **7**, **8**, *trans*-**10**-Tol$^{F}$) structure solution program using Intrinsic Phasing and refined with the ShelXL[7] (**3**, **4**, **7**, **8**, *trans*-**10**-Tol$^{F}$) refinement package using Least Squares minimization. **4** was refined with the olex2.refine refinement package[8] using Gauss-Newton minimization using NoSpherA2, an implementation of NOn-SPHERical Atom-form-factors in Olex2.[9] For **3**, the hydrogen atoms bonded to Ga1 were refined isotropically, the other hydrogen atoms were taken into account using a riding model. For **6**, phase transition was observed below 220 K. Ga1 (91:9) and Ga2 (57:43) are disordered over two sites. C37, C38, C41, C42, C44-C54, and C56-C66 are disordered over two sites (57:43). Anisotropic displacement parameters of disordered atoms were constrained pair wisely to be same. Suitable restraints and constraints were applied for the disordered atoms. A solvent mask was calculated, 90 electrons were found in a volume of 424 A$^{3}$ in 3 voids per unit cell. This is consistent with the presence of one toluene per asymmetric unit, which accounts for 100 electrons per unit cell. For **7**, a mixed crystal was measured with different atoms at the Ga2 atom. The Ga2 bound $Fe(CO)_4$ moiety has an occupancy of 96.9 % and the iodides an occupancy of 3.1%. For **8**, a solvent mask was calculated, 152 electrons were found in a volume of 952 A$^{3}$ in one void per unit cell. This is consistent with the presence of two benzene molecules per asymmetric unit, which accounts for 168 electrons per unit cell. *cis*-**10**-$C_6F_5$ contains highly disordered benzene near an inversion center, a solvent mask was calculated and 84 electrons were found in a volume of 936 A$^{3}$ in 3 voids per unit cell. This is consistent with the presence of 0.5[$C_6H_6$] per Asymmetric Unit, which account for 84 electrons per unit cell. For *trans*-**10**-$C_5NF_4$, highly disordered solvent could not be refined reliably, therefore a solvent mask was calculated and 426 electrons were found in a volume of 1735 A$^{3}$ in 1 void per unit cell. This is consistent with the presence of 2[$C_6H_5F$] per formula unit, which account for 400 electrons per unit cell. Disorder of C26, C27 over two sites (ratio 89:11), and disorder of C35, C36, C37, C38, F2, F3, F4, and F5 over two sites (ratio 54:46).

Details of the X-ray investigation are given in Table S1 and S2. CCDC 2334945, 2334948–2334953, 2379059, and 2379060 contain the supplementary crystallographic data for this paper. These data can be obtained free of charge from The Cambridge Crystallographic Data Centre via www.ccdc.cam.ac.uk/conts/retrieving.html.

**Table S1.** Crystallographic details of **3**, **4**, and **6**.

| | **3** x 2 $C_6H_6$ | **4** | **6** |
|---|---|---|---|
| Empirical formula | $C_{84}H_{100}Ga_2N_4$ | $C_{66}H_{78}Ga_2I_4N_4$ | $C_{73}Ga_2H_{86}N_4$ |
| Formula weight | 1305.11 | 1574.446 | 1158.89 |
| Temperature/K | 100.0(1) | 100.0(1) | 220.0(1) |
| Crystal system | tetragonal | triclinic | triclinic |
| Space group | $I4_1cd$ | P-1 | P-1 |
| a/Å | 17.2516(2) | 10.5694(1) | 12.7988(7) |
| b/Å | 17.2516(2) | 12.1801(1) | 14.6243(7) |
| c/Å | 47.4743(8) | 13.6704(1) | 18.1282(9) |
| α/° | 90 | 101.963(1) | 80.487(4) |
| β/° | 90 | 98.911(1) | 79.632(4) |
| γ/° | 90 | 107.530(1) | 85.811(4) |
| Volume/$Å^3$ | 14129.1(4) | 1596.52(3) | 3288.5(3) |
| Z | 8 | 1 | 2 |
| $\rho_{calc}g/cm^3$ | 1.227 | 1.638 | 1.170 |
| $\mu/mm^{-1}$ | 0.810 | 2.819 | 0.862 |
| F(000) | 5552.0 | 775.164 | 1228.0 |
| Crystal size/$mm^3$ | 0.07 × 0.069 × 0.026 | 0.36 × 0.14 × 0.13 | 0.28 × 0.24 × 0.15 |
| Radiation/Å | Mo Kα (λ = 0.71073) | Mo Kα (λ = 0.71073) | Mo Kα (λ = 0.71073) |
| 2Θ range for data collection/° | 5.35 to 60.058 | 6.4 to 75.58 | 6.434 to 51.364 |
| Index ranges | $-24 \leq h \leq 24, -24 \leq k \leq 24, -66 \leq l \leq 66$ | $-18 \leq h \leq 18, -20 \leq k \leq 20, -23 \leq l \leq 23$ | $-15 \leq h \leq 15, -17 \leq k \leq 17, -22 \leq l \leq 22$ |
| Reflections collected | 222973 | 157579 | 64721 |
| Independent reflections | 10335 [$R_{int}$ = 0.0863, $R_{sigma}$ = 0.0288] | 16604 [$R_{int}$ = 0.0310, $R_{sigma}$ = 0.0165] | 12461 [$R_{int}$ = 0.0486, $R_{sigma}$ = 0.0402] |
| Reflections with $I > 2\sigma(I)$ | 9117 | 14979 | 8211 |
| Data/restraints/parameters | 10335/1/426 | 16604/0/460 | 12461/648/897 |
| Goodness-of-fit on $F^2$ | 1.109 | 1.051 | 1.031 |
| Final R indexes [ $I > 2\sigma(I)$ ] | $R_1$ = 0.0336, $wR_2$ = 0.0780 | $R_1$ = 0.0180, $wR_2$ = 0.0365 | $R_1$ = 0.0645, $wR_2$ = 0.1573 |
| Final R indexes [all data] | $R_1$ = 0.0442, $wR_2$ = 0.0826 | $R_1$ = 0.0228, $wR_2$ = 0.0385 | $R_1$ = 0.1001, $wR_2$ = 0.1789 |
| Largest diff. peak/hole / e $Å^{-3}$ | 0.37/-0.26 | 1.33/-1.39 | 0.95/-0.72 |
| Flack parameter | 0.484(11) | – | – |
| CCDC number | 2334945 | 2334948 | 2334949 |

**Table S2.** Crystallographic details of **7**, **8**, and *trans*-**10**-Tol$^F$.

| | **7** | **8** | *trans*-**10**-Tol$^F$ |
|---|---|---|---|
| Empirical formula | $C_{82.88}H_{87}Fe_{1.97}Ga_2I_{0.07}N_4O_{7.88}$ | $C_{66}H_{78}Cl_4Ga_2N_4$ 2[$C_6H_6$] | $C_{80}H_{78}N_4F_{16}Ga_2$ |
| Formula weight | 1522.77 | 1364.77 | 1538.90 |
| Temperature/K | 100.0(1) | 173.0(1) | 100.0(1) |
| Crystal system | triclinic | monoclinic | monoclinic |
| Space group | P-1 | $P2_1/n$ | $P2_1/c$ |
| a/Å | 10.8571(2) | 10.3023(8) | 19.4377(4) |
| b/Å | 14.3020(3) | 18.4534(13) | 19.6182(4) |
| c/Å | 25.6182(5) | 20.0384(16) | 19.7964(5) |
| α/° | 98.115(2) | 90 | 90 |
| β/° | 96.616(2) | 103.039(7) | 100.679(2) |
| γ/° | 102.807(2) | 90 | 90 |
| Volume/Å$^3$ | 3795.66(13) | 3711.3(5) | 7418.3(3) |
| Z | 2 | 2 | 4 |
| $\rho_{calc}$g/cm$^3$ | 1.332 | 1.221 | 1.378 |
| μ/mm$^{-1}$ | 4.430 | 0.913 | 1.633 |
| F(000) | 1584.0 | 1432 | 3168.0 |
| Crystal size/mm$^3$ | 0.36 × 0.21 × 0.1 | 0.302 × 0.26 × 0.137 | 0.19 × 0.07 × 0.06 |
| Radiation/Å | Cu Kα (λ = 1.54184) | Mo Kα (λ = 0.71073) | Cu Kα (λ = 1.54184) |
| 2Θ range for data collection/° | 6.784 to 152.846 | 6.64 to 52.042 | 6.398 to 143.758 |
| Index ranges | $-13 \le h \le 13$, $-14 \le k \le 17$, $-32 \le l \le 32$ | $-12 \le h \le 12$, $-22 \le k \le 22$, $-21 \le l \le 24$ | $-10 \le h \le 23$, $-23 \le k \le 21$, $-24 \le l \le 23$ |
| Reflections collected | 34577 | 28520 | 29275 |
| Independent reflections | 15591 [$R_{int}$ = 0.0297, $R_{sigma}$ = 0.0346] | 7104 [$R_{int}$ = 0.0431, $R_{sigma}$ = 0.0439] | 14048 [$R_{int}$ = 0.0450, $R_{sigma}$ = 0.0618] |
| Reflections with $I > 2\sigma(I)$ | 14480 | 5704 | 10814 |
| Data/restraints/parameters | 15591/0/927 | 7104/0/351 | 14048/0/935 |
| Goodness-of-fit on $F^2$ | 1.021 | 1.044 | 1.026 |
| Final R indexes [ $I > 2\sigma(I)$ ] | $R_1$ = 0.0373, $wR_2$ = 0.0994 | $R_1$ = 0.0447, $wR_2$ = 0.1139 | $R_1$ = 0.0522, $wR_2$ = 0.1232 |
| Final R indexes [all data] | $R_1$ = 0.0405, $wR_2$ = 0.1027 | $R_1$ = 0.0577, $wR_2$ = 0.1219 | $R_1$ = 0.0728, $wR_2$ = 0.1350 |
| Largest diff. peak/hole / e Å$^{-3}$ | 0.87/-0.73 | 1.09/-0.54 | 1.67/-0.47 |
| CCDC number | 23349450 | 23349452 | 23349451 |

**Table S3.** Crystallographic details of *cis*-**10**-$C_6F_5$, *trans*-**10**-$C_5NF_4$, and **11**.

| | *cis*-**10**-$C_6F_5$ | *trans*-**10**-$C_5NF_4$ | **11**($CHCl_3$) |
|---|---|---|---|
| Empirical formula | $C_{81}H_{81}F_{12}Ga_2N_4$ | $C_{88}H_{88}F_{12}Ga_2N_6$ | C93 H101 Cl9 Ga2 N8 |
| Formula weight | 1478.000 | 1597.08 | 1789.30 |
| Temperature/K | 100.00(1) | 100.0(1) | 100.0(1) |
| Crystal system | monoclinic | monoclinic | triclinic |
| Space group | $P2_1/c$ | C2/c | P-1 |
| a/Å | 26.58465(18) | 21.1589(5) | 12.9660(4) |
| b/Å | 12.30259(7) | 19.2641(4) | 13.2339(5) |
| c/Å | 23.64645(14) | 19.4975(4) | 15.4127(4) |
| α/° | 90 | 90 | 110.468(3) |
| β/° | 106.0431(7) | 91.4975(4) | 90.678(2) |
| γ/° | 90 | 90 | 117.109(3) |
| Volume/Å$^3$ | 7432.61(8) | 7945.0(3) | 2158.55(14) |
| Z | 4 | 4 | 1 |
| $\rho_{calc}$g/cm$^3$ | 1.321 | 1.335 | 1.376 |
| μ/mm$^{-1}$ | 1.527 | 0.755 | 3.722 |
| F(000) | 3060.2 | 3312.0 | 930.0 |
| Crystal size/mm$^3$ | 0.59 × 0.08 × 0.07 | 0.39 × 0.26 × 0.13 | 0.21 × 0.14 × 0.07 |
| Radiation/Å | Cu Kα (λ = 1.54184) | Mo Kα (λ = 0.71073) | Cu Kα (λ = 1.54184) |
| 2Θ range for data collection/° | 7.6 to 151.96 | 6.544 to 60.16 | 7.826 to 152.008 |
| Index ranges | $-33 \leq h \leq 33$<br>$-15 \leq k \leq 15$<br>$-29 \leq l \leq 29$ | $-29 \leq h \leq 29$<br>$-27 \leq k \leq 27$<br>$-27 \leq l \leq 27$ | $-15 \leq h \leq 16$, $-15 \leq k \leq 16$, $-19 \leq l \leq 15$ |
| Reflections collected | 216854 | 139259 | 17814 |
| Independent reflections | 15470 [$R_{int}$ = 0.0284, $R_{sigma}$ = 0.0099] | 11652 [$R_{int}$ = 0.1053, $R_{sigma}$ = 0.0387] | 8769 [$R_{int}$ = 0.0430, $R_{sigma}$ = 0.0479] |
| Reflections with $I > 2\sigma(I)$ | 14587 | 10743 | 7850 |
| Data/restraints/parameters | 15470/0/881 | 11652/474/524 | 8769/0/495 |
| Goodness-of-fit on F$^2$ | 1.041 | 1.174 | 1.039 |
| Final R indexes [ $I > 2\sigma(I)$ ] | $R_1$ = 0.0297, w$R_2$ = 0.0804 | $R_1$ = 0.0675, w$R_2$ = 0.1644 | $R_1$ = 0.0492, w$R_2$ = 0.1270 |
| Final R indexes [all data] | $R_1$ = 0.0313, w$R_2$ = 0.0818 | $R_1$ = 0.0712, w$R_2$ = 0.1664 | $R_1$ = 0.0541, w$R_2$ = 0.1318 |
| Largest diff. peak/hole / e Å$^{-3}$ | 0.81/–0.46 | 0.54/–0.92 | 0.84/-1.16 |
| CCDC number | 2379059 | 2379060 | 2334953 |

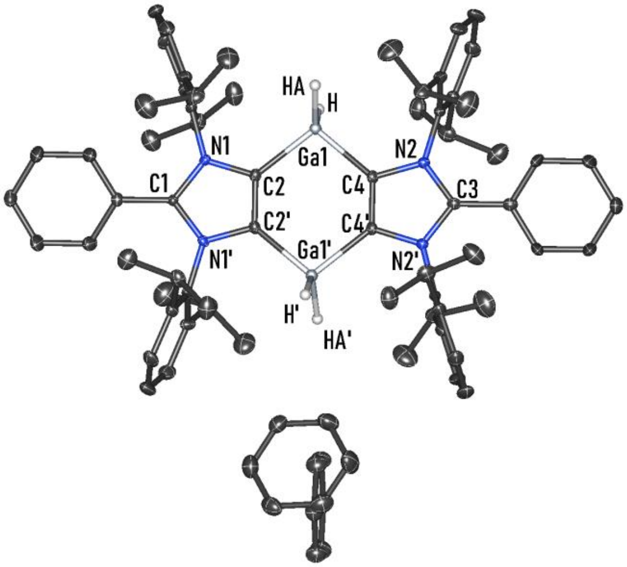


**Figure S50.** Solid state molecular structure of **3**. Hydrogen atoms (except bound to gallium) were omitted for clarity. Selected bond lengths (Å) and angles (°) for **3**: C2–Ga1 2.016(3), C4–Ga1 2.015(3), C2–C2′ 1.377(6), C4–C4′ 1.368(6), C2–Ga1–C4 101.2(1), C2′–C2–Ga1 128.9(1), C2′–C2–N1 105.9(2), C2–Ga1–H 108.1(2), C2–Ga1–Ha 113.7(2).

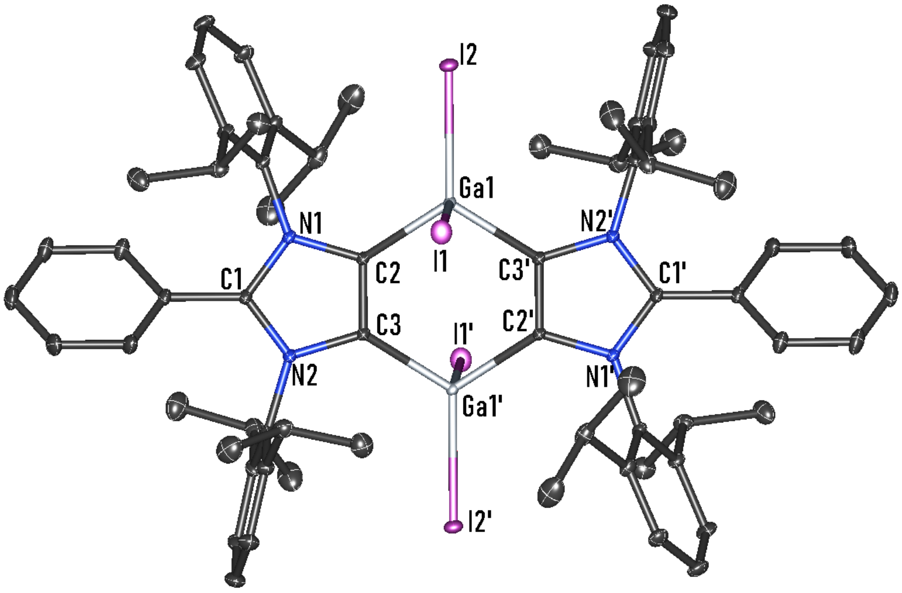


**Figure S51.** Solid state molecular structure of **4**. Hydrogen atoms were omitted for clarity. Selected bond lengths (Å) and angles (°) for **4**: C2–Ga1 1.993(1), C3′–Ga1 1.993(1), Ga1–I1 2.6117(1), Ga1–I2 2.5230(1), C2–C3 1.378(1), I1–Ga1–I2 107.47(1), C2–Ga1–I1 100.14(2), C2–Ga1–I2 117.85(2), C2–Ga1–C3′ 107.36(3), C3–C2–N1 106.5(1), C3–C2–Ga1 122.2(1).

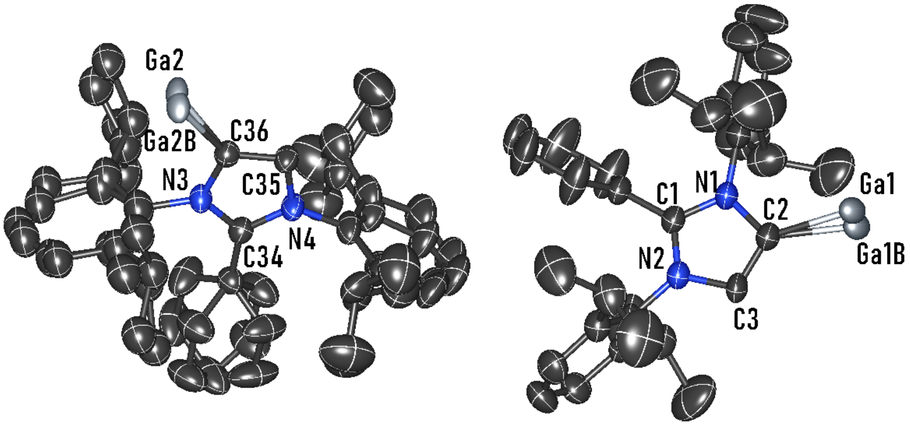


**Figure S52.** Solid state molecular structure of **6**. Hydrogen atoms were omitted for clarity. Selected bond lengths (Å) and angles (°) for **6**: C2–Ga1 2.092(4), C2–Ga1b 2.043(9), C3′–Ga1 2.107(4), C3′–Ga1b 1.994(9), C36′–Ga2 2.089(6), C36′–Ga2b 2.127(7), C35–Ga2 2.090(5), C35–Ga2b 2.092(7), C2–C3 1.388(5), C35–C36 1.382(5), C2–Ga1–C3′ 92.6(1), C2–Ga1b–C3′ 97.5(4), C35–Ga2–C36′ 93.2(2), C35–Ga2b–C36′ 92.0(3) C3–C2–Ga1 132.6(3), C3–C2–Ga1b 127.5(4), C3–C2–N1 105.2(3), C2–C3–N2 105.8(3).

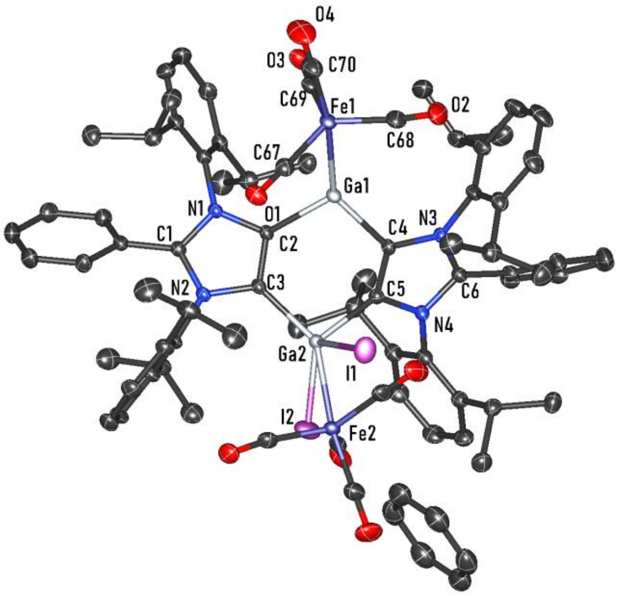


**Figure S53.** Solid state molecular structure of **7**. Hydrogen atoms were omitted for clarity. Selected bond lengths (Å) and angles (°) for **7**: C2–Ga1 2.021(2), C4–Ga1 2.010(2), C3–Ga2 2.009(2), C5–Ga2 2.028(2), Ga1–Fe1 2.327(1), Ga2–Fe2 2.320(1), Ga2–I1 2.772(4), Ga2–I2 2.313(5), C2–C3 1.383(2), C4–C5 1.376(2), C3–C2–N1 106.0(1), C2–C3–N2 106.2(2), C5–C4–N3 106.6(2), C4–C5–N4 106.1(2), C2–Ga1–C4 99.9(1), C2–Ga1–Fe1 119.6(1), C4–Ga1–Fe1 139.7(1), C3–Ga2–C5 99.8(1), C3–Ga2–Fe2 139.2(1), C5–Ga2–Fe2 120.3(1).

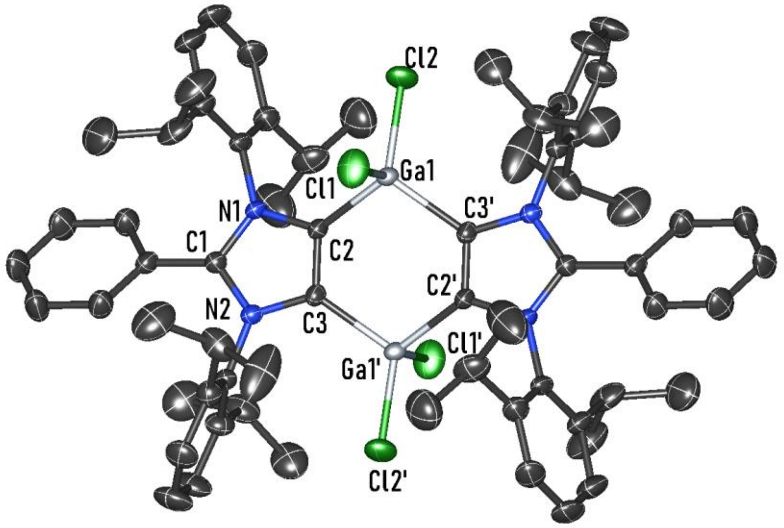


**Figure S54.** Solid state molecular structure of **8**. Hydrogen atoms were omitted for clarity. Selected bond lengths (Å) and angles (°) for **8**: C2–Ga1 1.981(3), C3′–Ga1 1.992(2), Ga1–Cl1 2.225(1), Ga1–Cl2 2.159(1), C2–C3 1.359(4), C3–C2–N1 106.2(2), C2–C3–N2 106.9(2), C2–Ga1–C3′ 107.5(1), Cl1–Ga1–Cl2 106.4(1), C2–Ga1–Cl1 103.5(1), C2–Ga1–Cl2 117.5(1).

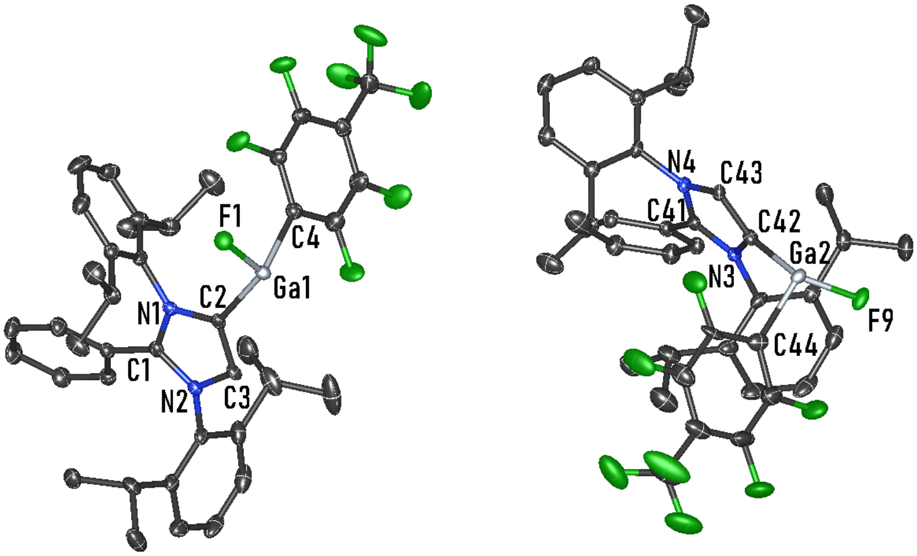


**Figure S55.** Solid state molecular structure of *trans*-**10**-Tol$^{F}$. Hydrogen atoms were omitted for clarity. Selected bond lengths (Å) and angles (°) for **10**-Tol$^{F}$: C2–Ga1 1.997(3), C3′–Ga1 1.997(3), C4–Ga1 2.017(3), Ga1–F1 1.795(2), C2–C3 1.375(4), C42–Ga2 1.992(3), C43′–Ga2 1.991(3), C44–Ga2 2.030(3), Ga2–F9 1.787(2), C42–C43 1.375(4), C3–C2–N1 106.5(3), C2–C3–N2 106.2(3), C2–Ga1–C3′ 106.5(1), C2–Ga1–C4 109.9(1), C2–Ga1–F1 112.9(1).

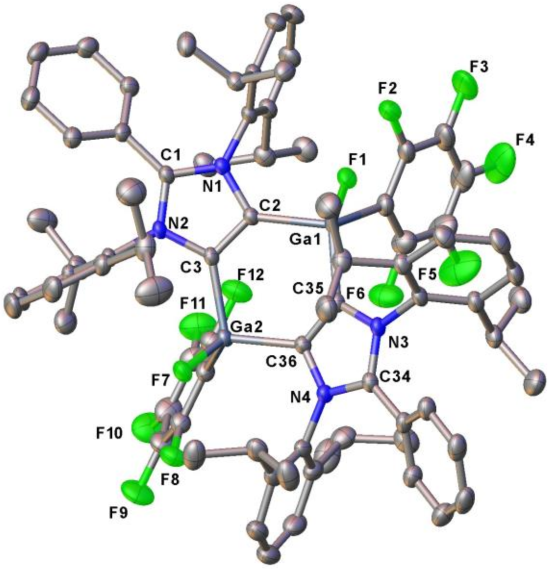


**Figure S56.** Solid state molecular structure of *cis*-**10**-$C_6F_5$. Hydrogen atoms were omitted for clarity. Selected bond lengths (Å) and angles (°) for *cis*-**10**-$C_6F_5$: C2–Ga1 1.999(1), C35–Ga1 2.003(1), C67–Ga1 2.010(1), Ga1–F1 1.800(1), C2–C3 1.369(2), C3–Ga2 1.982(1), C36–Ga2 1.984(1), C73–Ga2 2.005(1), Ga2–F7 1.798(1), C35–C36 1.370(2), C3–C2–N1 105.9(1), C2–C3–N2 106.9(1), C2–Ga1–C35 103.8(1), C2–Ga1–C67 115.6(1), C2–Ga1–F1 107.4(1).

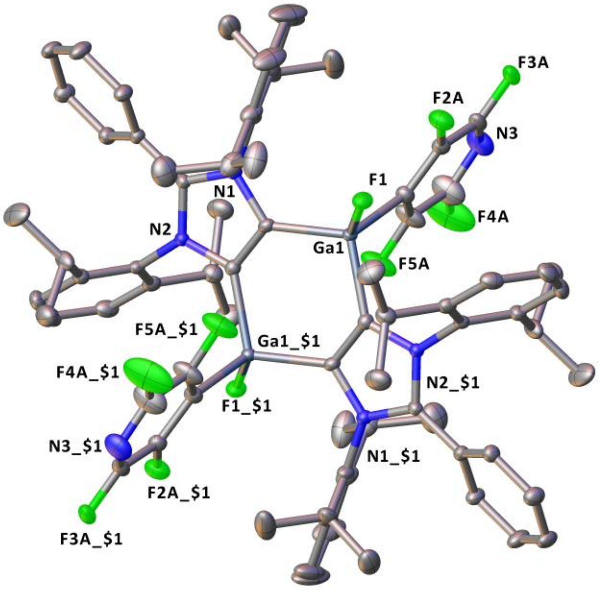


**Figure S57.** Solid state molecular structure of *trans*-**10**-$C_5NF_4$. Hydrogen atoms were omitted for clarity. Selected bond lengths (Å) and angles (°) for *trans*-**10**-$C_5NF_4$: C2–Ga1 1.990(3), C3′–Ga1 1.997(3), C34–Ga1 2.023(3), Ga1–F1 1.792(2), C2–C3 1.374(4), C3–C2–N1 106.1(2), C2–C3–N2 106.8(2), C2–Ga1–C3′ 106.4(1), C2–Ga1–C34 109.7(1), C2–Ga1–F1 112.3(1).

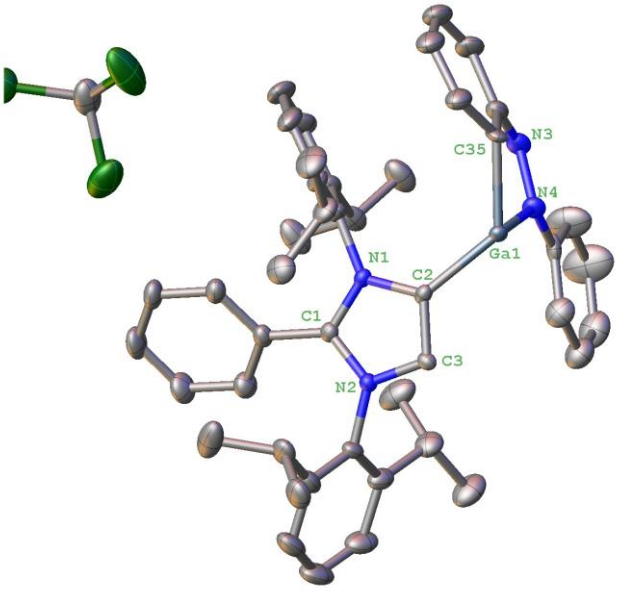


**Figure S58.** Solid state molecular structure of **11**. Hydrogen atoms were omitted for clarity. Selected bond lengths (Å) and angles (°) for **11**: C2–Ga1 2.009(2), C3'–Ga1 2.008(2), Ga1–C35 2.052(2), Ga1–N4 1.969(2), N3–N4 1.394(3), N3–C34 1.294(4), C34–C35 1.472(4), C2–Ga1–C3' 103.8(1), N4–Ga1–C35 82.4(1).

# Computational Details

The molecular structure of compound **6** was optimized at the $r^2$SCAN-3c level of theory.[10] Additionally, energies were calculated using the PBE0-D3BJ/def2-TZVPP approximation.[11] For this, the Orca 5.0.4 software package was utilized.[12] As the starting approximation, the solid-state molecular structure from the sc-XRD was taken. The convergence for optimization was set to TightOpt. In all Orca calculations, we used the settings TightSCF and DefGrid3, as well as the RIJCOSX accelerating approximation.[13] The electronic structure solutions, namely closed shell singlet (**CS**, using RKS), open shell singlet diradical (**OS**, UKS), and open shell triplet (**T**, UKS) were tested. To obtain a singlet diradical solution, the triplet solution was taken and the electron spin at one of the Ga atoms was flipped. All these calculations converged effectively to RKS singlet (**CS**) solutions. The results are summarized in Table S4. Thus, compound **6** has a **CS** ground state. The optimized molecular structure is shown in Figure S62.

To investigate the electron correlation, we performed fractional occupation weighted density (FOD) analyses[14] at the PBE0/def2-TZVPP ($T_{el}$ = 10000 K) level of theory. The calculation produced $N_{FOD}$ = 2.85 *e* for **6**, suggesting a moderate level of electron correlation. The FOD plot (Figure S66) shows that the respective (HOT) electron density is mainly located at the Ga and the adjacent C atoms.

The UV-Vis spectrum for **6** was modeled using TD-DFT calculations with TDA approximation at the PBE0/def2-TZVPP level of theory, and applying the CPCM scheme for taking into account THF solvent. Selected transitions are summarized in Table S6. The TD-DFT plot of the modeled spectrum is shown in Figure S68. Frontier molecular orbitals (FMOs) are depicted in Figure S64. Natural bond orbital (NBO) analyses[15] were performed using NBO 6.0.18a program[16] within Gaussian 16 package.[17] The wavefunction in this analysis was calculated at the RKS-PBE0/def2-TZVPP level for the optimized molecular geometry. The NBO charges and Wiberg Bond Indices (WBIs) for selected atoms and atom pairs, respectively, are collected in Table S7.

In addition to compound **6** (Figure S62), we also computationally investigated compounds **4** (Figure S59) and **5** (Figure S60) as well as the isomer **5′** (Figure S61) using the similar approximations (see above). In the isomer **5′**, each three-coordinated Ga(II) atom has an iodide substituent and an unpaired electron. Compound **5′** has the lowest energy for the **T** electronic state, while the **CS** is energetically only 0.26 kcal/mol high-lying. The other two molecules **4** and **5** have **CS** ground states. The mixed-valent $Ga^{I}/Ga^{III}$ compound **5** was calculated to be energetically more stable by 17.6 kcal/mol in comparison to **5′** with formally $Ga^{II}$ atoms. FOD calculations for **5** produced $N_{FOD}$ = 2.50 *e* with large localization at the two-coordinated Ga(I) atom (Figure S65). On the basis of TD-DFT calculations, the UV-Vis spectrum for **5** was simulated (Figure S67). Selected transitions are listed in Table S5. Selected frontier molecular orbitals (FMOs) of **5** are shown in Figure S63. Moreover, the NBO analyses were performed for **4** and **5**. The results are summarized in Table S7.

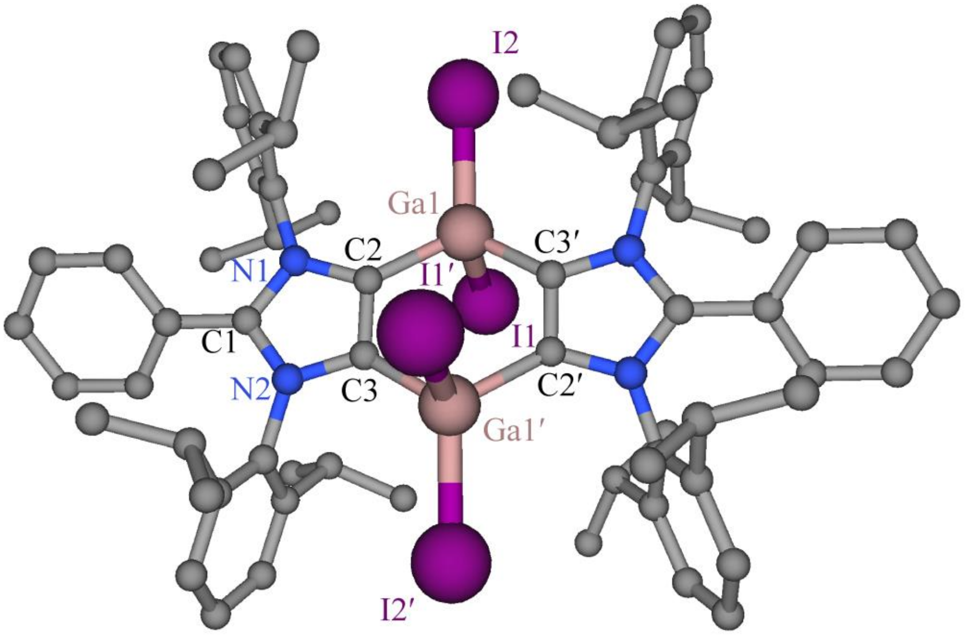


**Figure S59.** Optimized molecular structure of **4** ($C_i$ symmetry) at the r$^2$SCAN-3c level. Hydrogen atoms are omitted for clarity. Selected equilibrium parameters (Å, °) are: Ga1–I2 2.553, Ga1–I1 2.639, C2–Ga1 2.000, C1–N1 1.357, C1–N2 1.355, C2–N1 1.392, C2–C3 1.378, I1–Ga1–I2 108.8, C2–Ga1–I2 101.0, C2–Ga1–I1 117.6, C3–C2–Ga1 121.7, Ga1–C2–C3–N2 160.7.

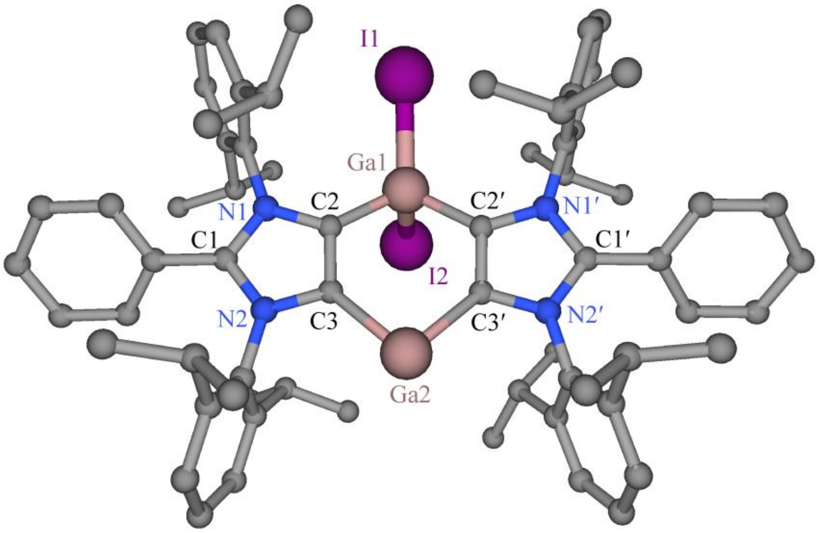


**Figure S60.** Optimized molecular structure of **5** ($C_1$ symmetry) at the r$^2$SCAN-3c level. Hydrogen atoms are omitted for clarity. Ga1–I2 bond is approximately in the plane of the image; Ga1–I1 bond is oriented approximately in the direction away from the reader. Selected equilibrium parameters (Å, °) are: C3–Ga2 2.133, C2–Ga1 1.990, C2–C3 1.384, Ga1–I2 2.566, Ga1–I1 2.646, C2–C3–Ga2 129.8, C3–C2–Ga1 119.3, I1–Ga1–I2 109.1, N2–C2–C3–Ga1 160.2, N1–C2–C3–Ga2 153.4.

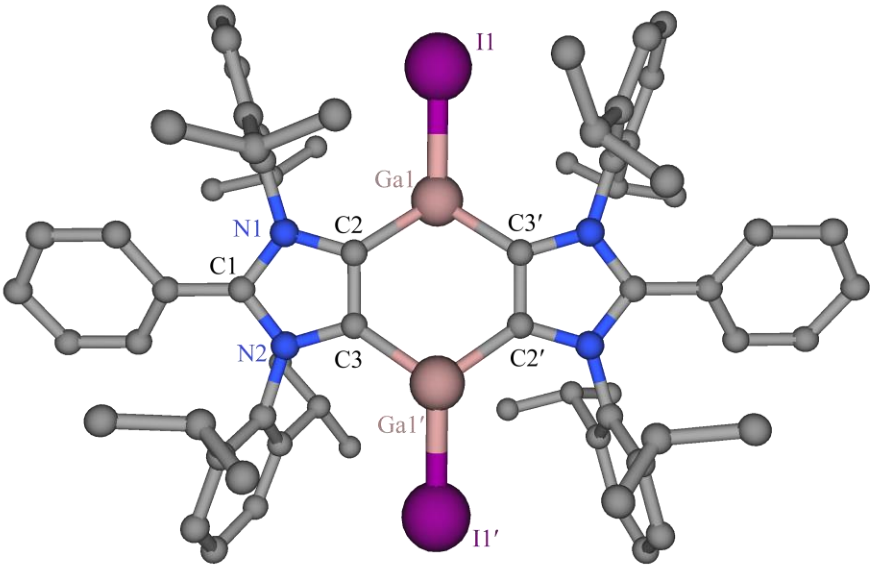


**Figure S61.** Optimized molecular structure of **5′** ($C_i$ symmetry) at the r²SCAN-3c level. Hydrogen atoms are omitted for clarity. Selected equilibrium parameters (Å, °) are: C2–Ga1 1.968, C3′–Ga1 1.987, Ga1–I1 2.575, C2–C3 1.395, C2–Ga1–I1 120.3, C3–C2–Ga1 121.8, N2–C3–C2–Ga1 160.9.

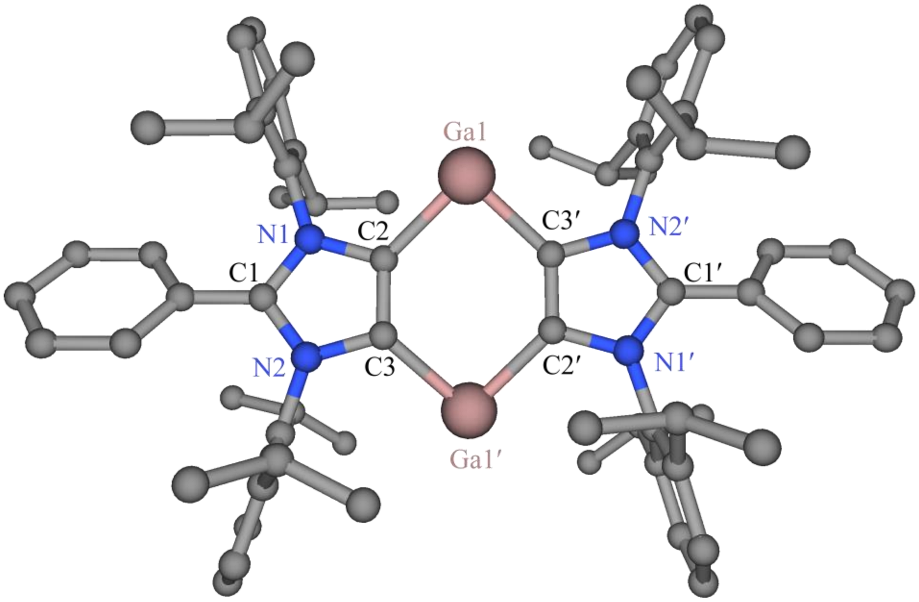


**Figure S62.** Optimized molecular structure of **6** ($C_i$ symmetry) at the r²SCAN-3c level. Hydrogen atoms are omitted for clarity. Selected equilibrium parameters (Å, °) are: Ga1–C2 = 2.128, Ga1–C3′ = 2.113, C2–C3 = 1.393, N1–C2 = 1.408, C1–N1 = 1.352, C2–Ga1–C3′ = 91.8, C3–C2–Ga1 = 131.3, C2–C3–Ga1′ = 127.9, Ga1′–C3–C2–Ga1 = –42.5, C3–C2–Ga1–C3′ = 32.2.

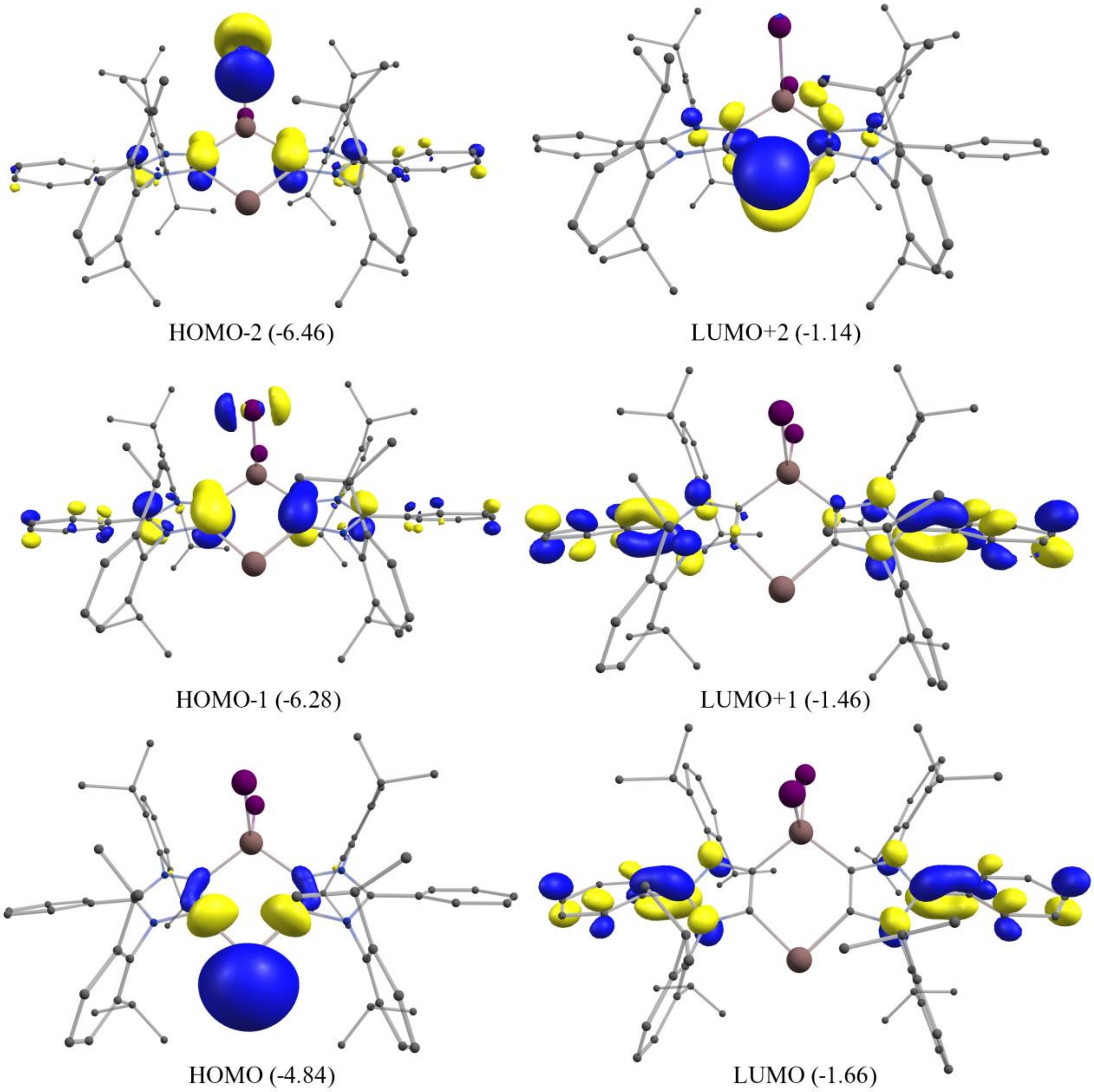


**Figure S63.** Frontier molecular orbitals (0.05 a.u. isosurfaces) with respective energies (in eV) of **5** in RKS-PBE0/def2-TZVPP calculation.

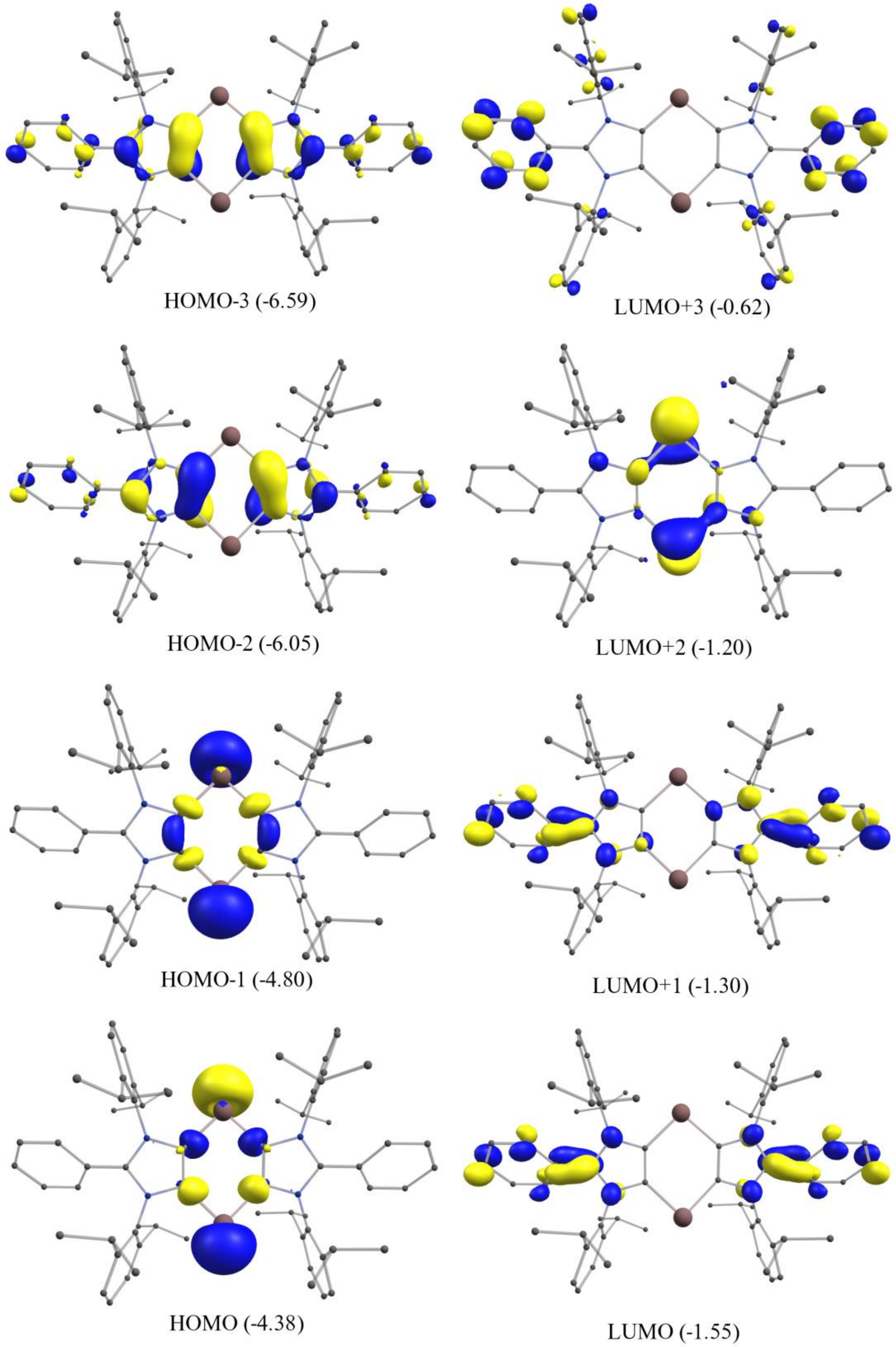


**Figure S64.** Frontier molecular orbitals (0.05 a.u. isosurfaces) with respective energies (in eV) of **6** in RKS-PBE0/def2-TZVPP calculation.

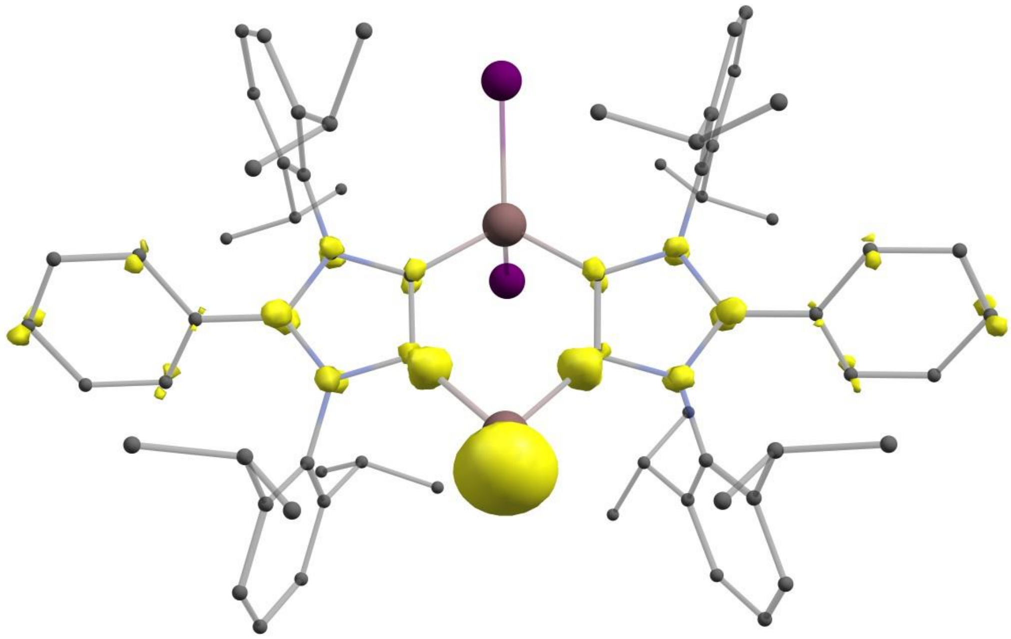

**Figure S65.** FOD plot (isosurfaces 0.005 a.e. in yellow) of **5**. Hydrogen atoms are omitted for clarity.

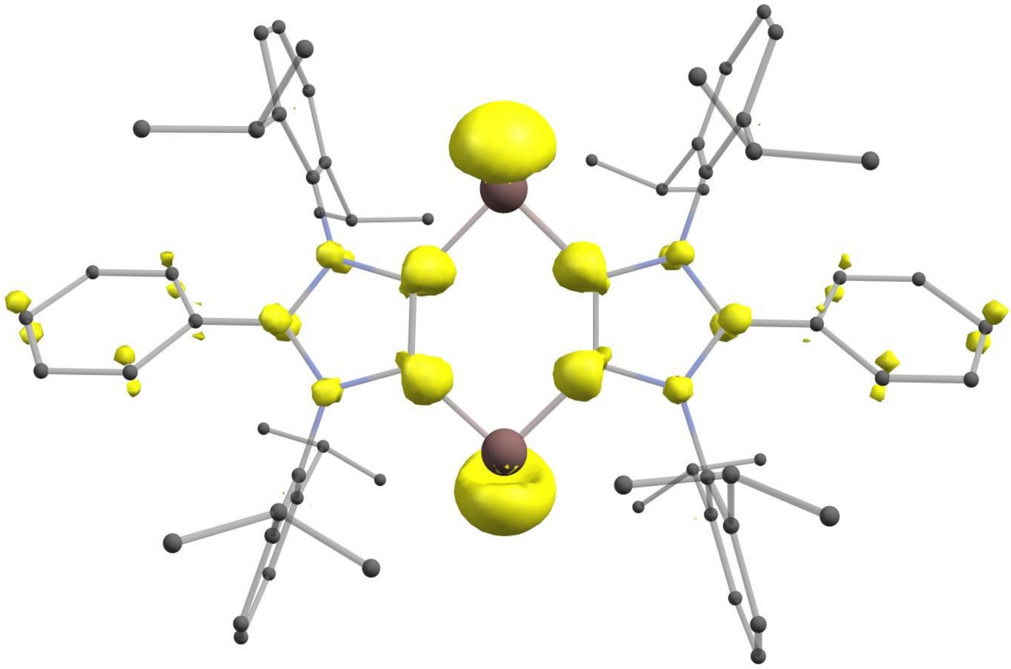

**Figure S66.** FOD plot (isosurfaces 0.005 a.e. in yellow) of **6**. Hydrogen atoms are omitted for clarity.

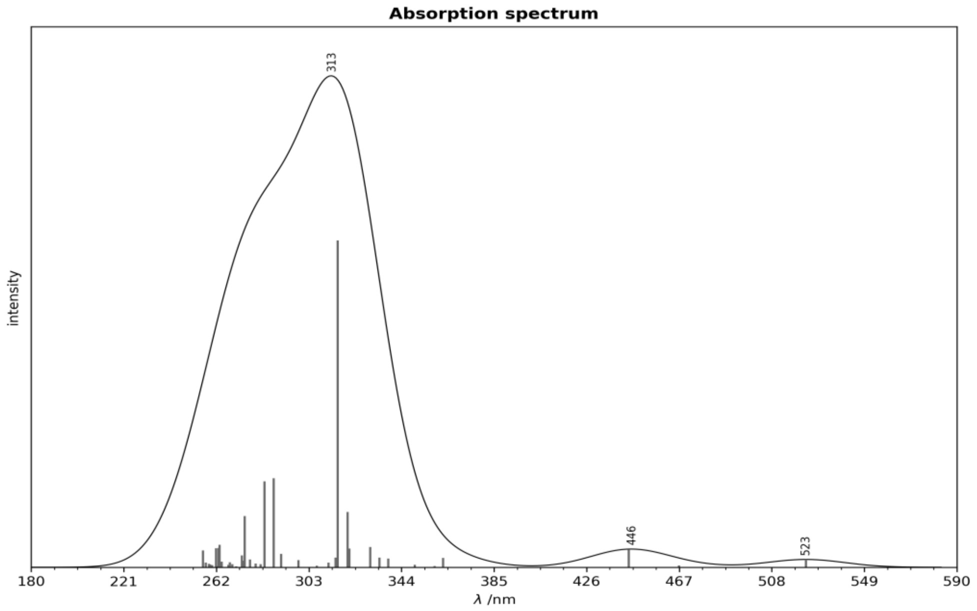


**Figure S67.** Plot of UV-Vis spectrum of **5** obtained from TD-DFT calculations.

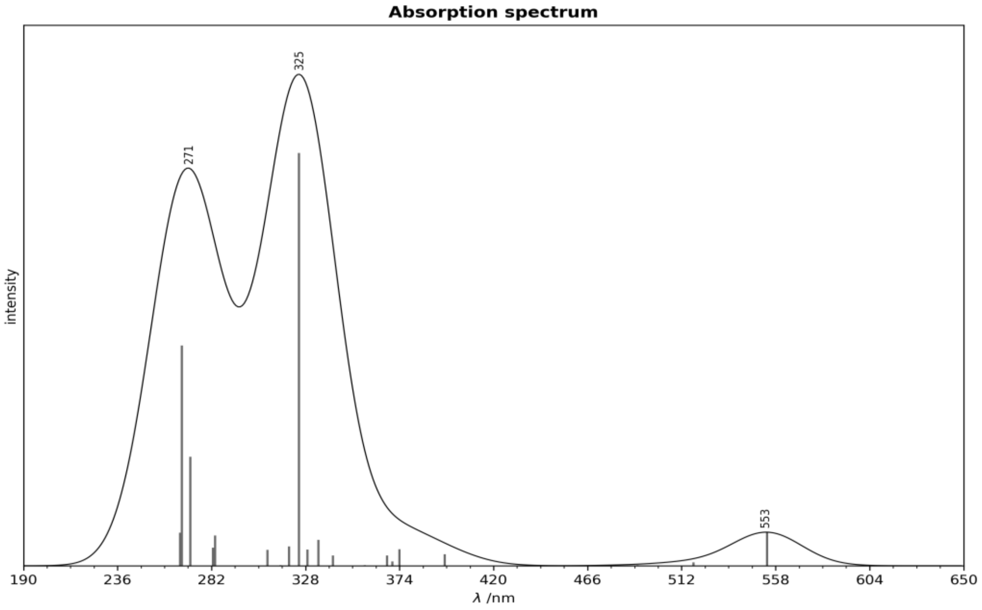


**Figure S68.** Plot of UV-Vis spectrum of **6** obtained from TD-DFT calculations.

**Table S4.** Relative energies [in kcal/mol] of **6** for different electronic solutions.

| Electronic solution | **6** | |
|---|---|---|
| | PBE0-D3BJ/def2-TZVPP[a] | $r^2$SCAN-3c[b] |
| CS | 0.0 | 0.0 |
| OS | –[c] | –[c] |
| T | 37.4 | 12.8 |

[a] Vertical energies calculated for structures optimized at the $r^2$SCAN-3c level.
[b] Adiabatic energies.
[c] SCF has converged to the CS solution.

**Table S5.** Most important transitions in the TD-DFT absorption spectrum of **5**. Wavelengths $\lambda$ in nm, oscillator strengths *f* via transition electric dipole moments and assignments are listed.

| $\lambda$, nm | *f* | Assignment |
|---|---|---|
| 523 | 0.02 | 91 % HOMO → LUMO |
| 445 | 0.04 | 90 % HOMO → LUMO+2 |
| 316 | 0.81 | 82 % HOMO-1 → LUMO |

**Table S6.** Most important transitions in the TD-DFT absorption spectrum of **6**. Wavelengths $\lambda$ in nm, oscillator strengths *f* via transition electric dipole moments and assignments are listed.

| $\lambda$, nm | *f* | Assignment |
|---|---|---|
| 553 | 0.09 | 76 % HOMO → LUMO+2; 17 % HOMO → LUMO+1 |
| 325 | 1.11 | 94 % HOMO-2 → LUMO |
| 271 | 0.29 | 72 % HOMO-3 → LUMO+2; 17 % HOMO-3 → LUMO+1 |
| 267 | 0.59 | 42 % HOMO-3 → LUMO+1; 21 % HOMO-1 → LUMO+17 |

**Table S7.** NBO charges and WBIs for compounds **4**, **5**, and **6** (all in $C_i$ symmetry).

| | | | | | |
|---|---|---|---|---|---|
| 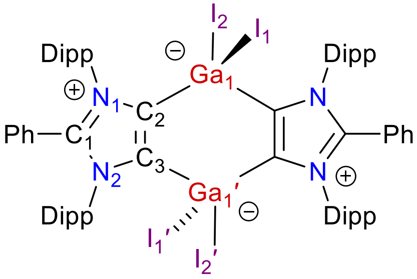 **4** | | 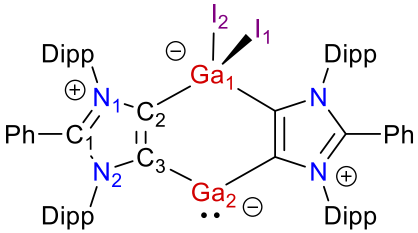 **5** | | 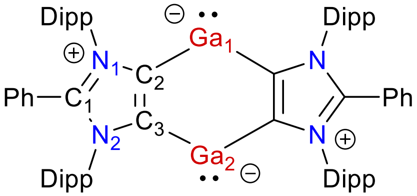 **6** | |
| **NBO (PBE0/def2-TZVPP) Charges** | | | | | |
| Atom | *q* | Atom | *q* | Atom | *q* |
| Ga1 | 1.02 | Ga1 | 1.02 | Ga1 | 0.48 |
| | | Ga2 | 0.52 | | |
| I1 | –0.41 | I1 | –0.43 | C2 | –0.33 |
| I2 | –0.34 | I2 | –0.36 | | |
| C1 | 0.42 | C1 | 0.40 | C1 | 0.38 |
| C3 | –0.31 | C3 | –0.30 | C3 | –0.34 |
| - | - | C2 | –0.35 | | |
| - | - | N1 | –0.35 | N1 | –0.37 |
| N2 | –0.34 | N2 | –0.36 | N2 | –0.36 |
| **WBIs (PBE0/def2-TZVPP) for Selected Atom Pairs** | | | | | |
| Atom pair | WBI | Atom pair | WBI | Atom pair | WBI |
| Ga1–I1 | 0.75 | Ga1–I1 | 0.74 | - | - |
| Ga1–I2 | 0.89 | Ga1–I2 | 0.86 | - | - |
| Ga1–C2 | 0.54 | Ga1–C2 | 0.56 | Ga1–C2 | 0.47 |
| - | - | Ga2–C3 | 0.45 | Ga1–C3′ | 0.47 |
| N1–C1 | 1.25 | N1–C1 | 1.25 | N1–C1 | 1.25 |
| C2–N1 | 1.12 | C2–N1 | 1.10 | N1–C2 | 1.09 |
| C1–N2 | 1.26 | C1–N2 | 1.24 | N2–C1 | 1.25 |
| N2–C3 | 1.11 | N2–C3 | 1.10 | N2–C3 | 1.09 |
| C2–C3 | 1.58 | C2–C3 | 1.57 | C2–C3 | 1.57 |

# Cartesian Coordinates

Optimized Cartesian coordinates of **4** (RKS singlet) at the r$^2$SCAN-3c level.

--------------------------------------------------------------------------------

| | | | |
|---|---|---|---|
| I | 1.204319337 | 0.024829215 | -3.202244917 |
| I | 1.319907462 | -3.795839496 | -1.413265618 |
| Ga | 0.674440612 | -1.357599099 | -1.018101593 |
| N | -2.376197912 | -1.574973520 | -1.313882754 |
| N | -3.036478118 | 0.393757198 | -0.686331345 |
| C | -3.454555716 | -0.751471760 | -1.277915896 |
| C | -1.265482913 | -0.942581879 | -0.762960176 |
| C | -1.691393939 | 0.297448718 | -0.338755080 |
| C | -4.799897211 | -1.046778422 | -1.777244587 |
| C | -5.353713857 | -2.330148487 | -1.639007011 |
| H | -4.796142131 | -3.119546829 | -1.152866224 |
| C | -6.627448558 | -2.603528225 | -2.117325734 |
| H | -7.034466053 | -3.602740517 | -1.995603154 |
| C | -7.377347921 | -1.612097368 | -2.741847195 |
| H | -8.373249520 | -1.831251496 | -3.115298025 |
| C | -6.838799929 | -0.337565622 | -2.882802158 |
| H | -7.408617943 | 0.447452637 | -3.370758333 |
| C | -5.565994622 | -0.053828409 | -2.407720855 |
| H | -5.169448605 | 0.944350572 | -2.533142329 |
| C | -2.407470390 | -2.948492483 | -1.753206236 |
| C | -2.474041144 | -3.223838555 | -3.122187886 |
| C | -2.611535544 | -4.563193420 | -3.496690300 |
| H | -2.670156829 | -4.816933791 | -4.550820305 |
| C | -2.662370049 | -5.567669372 | -2.545469924 |
| H | -2.771640754 | -6.602811106 | -2.856427779 |
| C | -2.541392429 | -5.263705271 | -1.193766561 |
| H | -2.539878056 | -6.069178998 | -0.468003070 |

| | | | |
|---|---|---|---|
| C | -2.401280706 | -3.947581165 | -0.760452385 |
| C | -2.369938548 | -2.155333162 | -4.193425526 |
| H | -2.183480229 | -1.189576895 | -3.707598025 |
| C | -1.173854904 | -2.438522561 | -5.114722535 |
| H | -0.254446903 | -2.578050468 | -4.540048106 |
| H | -1.346349520 | -3.336526887 | -5.718434147 |
| H | -1.020552168 | -1.596049701 | -5.797132313 |
| C | -3.663328110 | -2.041635429 | -5.014152203 |
| H | -3.850951649 | -2.965675787 | -5.572684739 |
| H | -4.533992752 | -1.846987265 | -4.381804061 |
| H | -3.574628176 | -1.225802687 | -5.740106571 |
| C | -2.258833345 | -3.618986740 | 0.719344142 |
| H | -1.551734623 | -2.784505896 | 0.809096577 |
| C | -3.584524246 | -3.153619514 | 1.344669651 |
| H | -3.979138647 | -2.251582838 | 0.868386628 |
| H | -4.344762517 | -3.940961816 | 1.271000429 |
| H | -3.429575892 | -2.918423943 | 2.402662842 |
| C | -1.685896462 | -4.779990430 | 1.534956483 |
| H | -2.404595677 | -5.601526910 | 1.635358828 |
| H | -0.766213079 | -5.168640027 | 1.086148782 |
| H | -1.452172374 | -4.427574741 | 2.544208744 |
| C | -3.776867207 | 1.630633777 | -0.635464811 |
| C | -3.540326645 | 2.553550791 | -1.667944084 |
| C | -4.266255326 | 3.742731861 | -1.633034948 |
| H | -4.107242084 | 4.487244115 | -2.407682309 |
| C | -5.186932739 | 3.988538731 | -0.622381821 |
| H | -5.744392721 | 4.920901985 | -0.615289658 |
| C | -5.387471537 | 3.058023587 | 0.386270802 |
| H | -6.097177890 | 3.271902311 | 1.179409273 |
| C | -4.675628547 | 1.856927504 | 0.411282227 |
| C | -2.546588904 | 2.301445647 | -2.791146083 |

| | | | |
|---|---|---|---|
| H | -2.156602567 | 1.281023417 | -2.703767984 |
| C | -3.186347544 | 2.427289729 | -4.181067503 |
| H | -3.536660490 | 3.447034505 | -4.373740318 |
| H | -2.443328815 | 2.180743797 | -4.947096659 |
| H | -4.035309468 | 1.747166141 | -4.306706683 |
| C | -1.343685034 | 3.244802208 | -2.669277681 |
| H | -1.651599884 | 4.292277240 | -2.768903012 |
| H | -0.855418648 | 3.134088938 | -1.697387327 |
| H | -0.610490354 | 3.016576059 | -3.449423787 |
| C | -4.888184959 | 0.862914275 | 1.535901175 |
| H | -4.079612584 | 0.123048282 | 1.495729110 |
| C | -4.805484786 | 1.538668783 | 2.910633955 |
| H | -3.887710682 | 2.125265444 | 3.008594215 |
| H | -5.662888231 | 2.198086390 | 3.085872336 |
| H | -4.803888309 | 0.776235904 | 3.696095504 |
| C | -6.227061373 | 0.125697537 | 1.376592684 |
| H | -6.344557427 | -0.616342309 | 2.174205414 |
| H | -7.063661610 | 0.831007169 | 1.443933006 |
| H | -6.299129796 | -0.389258925 | 0.413849615 |
| I | -1.204319337 | -0.024829215 | 3.202244917 |
| I | -1.319907462 | 3.795839496 | 1.413265618 |
| Ga | -0.674440612 | 1.357599099 | 1.018101593 |
| N | 2.376197912 | 1.574973520 | 1.313882754 |
| N | 3.036478118 | -0.393757198 | 0.686331345 |
| C | 3.454555716 | 0.751471760 | 1.277915896 |
| C | 1.265482913 | 0.942581879 | 0.762960176 |
| C | 1.691393939 | -0.297448718 | 0.338755080 |
| C | 4.799897211 | 1.046778422 | 1.777244587 |
| C | 5.353713857 | 2.330148487 | 1.639007011 |
| H | 4.796142131 | 3.119546829 | 1.152866224 |
| C | 6.627448558 | 2.603528225 | 2.117325734 |

| | | | |
|---|---|---|---|
| H | 7.034466053 | 3.602740517 | 1.995603154 |
| C | 7.377347921 | 1.612097368 | 2.741847195 |
| H | 8.373249520 | 1.831251496 | 3.115298025 |
| C | 6.838799929 | 0.337565622 | 2.882802158 |
| H | 7.408617943 | -0.447452637 | 3.370758333 |
| C | 5.565994622 | 0.053828409 | 2.407720855 |
| H | 5.169448605 | -0.944350572 | 2.533142329 |
| C | 2.407470390 | 2.948492483 | 1.753206236 |
| C | 2.474041144 | 3.223838555 | 3.122187886 |
| C | 2.611535544 | 4.563193420 | 3.496690300 |
| H | 2.670156829 | 4.816933791 | 4.550820305 |
| C | 2.662370049 | 5.567669372 | 2.545469924 |
| H | 2.771640754 | 6.602811106 | 2.856427779 |
| C | 2.541392429 | 5.263705271 | 1.193766561 |
| H | 2.539878056 | 6.069178998 | 0.468003070 |
| C | 2.401280706 | 3.947581165 | 0.760452385 |
| C | 2.369938548 | 2.155333162 | 4.193425526 |
| H | 2.183480229 | 1.189576895 | 3.707598025 |
| C | 1.173854904 | 2.438522561 | 5.114722535 |
| H | 0.254446903 | 2.578050468 | 4.540048106 |
| H | 1.346349520 | 3.336526887 | 5.718434147 |
| H | 1.020552168 | 1.596049701 | 5.797132313 |
| C | 3.663328110 | 2.041635429 | 5.014152203 |
| H | 3.850951649 | 2.965675787 | 5.572684739 |
| H | 4.533992752 | 1.846987265 | 4.381804061 |
| H | 3.574628176 | 1.225802687 | 5.740106571 |
| C | 2.258833345 | 3.618986740 | -0.719344142 |
| H | 1.551734623 | 2.784505896 | -0.809096577 |
| C | 3.584524246 | 3.153619514 | -1.344669651 |
| H | 3.979138647 | 2.251582838 | -0.868386628 |
| H | 4.344762517 | 3.940961816 | -1.271000429 |

| | | | |
|---|---|---|---|
| H | 3.429575892 | 2.918423943 | -2.402662842 |
| C | 1.685896462 | 4.779990430 | -1.534956483 |
| H | 2.404595677 | 5.601526910 | -1.635358828 |
| H | 0.766213079 | 5.168640027 | -1.086148782 |
| H | 1.452172374 | 4.427574741 | -2.544208744 |
| C | 3.776867207 | -1.630633777 | 0.635464811 |
| C | 3.540326645 | -2.553550791 | 1.667944084 |
| C | 4.266255326 | -3.742731861 | 1.633034948 |
| H | 4.107242084 | -4.487244115 | 2.407682309 |
| C | 5.186932739 | -3.988538731 | 0.622381821 |
| H | 5.744392721 | -4.920901985 | 0.615289658 |
| C | 5.387471537 | -3.058023587 | -0.386270802 |
| H | 6.097177890 | -3.271902311 | -1.179409273 |
| C | 4.675628547 | -1.856927504 | -0.411282227 |
| C | 2.546588904 | -2.301445647 | 2.791146083 |
| H | 2.156602567 | -1.281023417 | 2.703767984 |
| C | 3.186347544 | -2.427289729 | 4.181067503 |
| H | 3.536660490 | -3.447034505 | 4.373740318 |
| H | 2.443328815 | -2.180743797 | 4.947096659 |
| H | 4.035309468 | -1.747166141 | 4.306706683 |
| C | 1.343685034 | -3.244802208 | 2.669277681 |
| H | 1.651599884 | -4.292277240 | 2.768903012 |
| H | 0.855418648 | -3.134088938 | 1.697387327 |
| H | 0.610490354 | -3.016576059 | 3.449423787 |
| C | 4.888184959 | -0.862914275 | -1.535901175 |
| H | 4.079612584 | -0.123048282 | -1.495729110 |
| C | 4.805484786 | -1.538668783 | -2.910633955 |
| H | 3.887710682 | -2.125265444 | -3.008594215 |
| H | 5.662888231 | -2.198086390 | -3.085872336 |
| H | 4.803888309 | -0.776235904 | -3.696095504 |
| C | 6.227061373 | -0.125697537 | -1.376592684 |

| | | | |
|---|---|---|---|
| H | 6.344557427 | 0.616342309 | -2.174205414 |
| H | 7.063661610 | -0.831007169 | -1.443933006 |
| H | 6.299129796 | 0.389258925 | -0.413849615 |

--------------------------------------------------------------------

Optimized Cartesian coordinates of **5** (RKS singlet) at the r$^2$SCAN-3c level.

--------------------------------------------------------------------------------

| | | | |
|---|---|---|---|
| Ga | 0.69600315002191 | -1.72225383539395 | -0.87777539081627 |
| N | -2.38045685826612 | -1.60774006651531 | -1.12648549062545 |
| N | -3.02029112379306 | 0.41452670640028 | -0.69536420476719 |
| C | -3.43385149289043 | -0.75933905882796 | -1.23003071897959 |
| C | -1.27601578645314 | -0.98549241627789 | -0.53421551067795 |
| C | -1.70374902561820 | 0.29766150113461 | -0.24157783612418 |
| C | -4.73730616877915 | -1.05518409622315 | -1.82503617399316 |
| C | -5.33139832526624 | -2.31732238149366 | -1.65940631455217 |
| H | -4.83546642780118 | -3.08257730696195 | -1.07641081376714 |
| C | -6.56394815964010 | -2.59708790123194 | -2.23218844071344 |
| H | -7.00503592753229 | -3.57859364252463 | -2.08661686205167 |
| C | -7.23209411985315 | -1.63273157557451 | -2.98088451204055 |
| H | -8.19664763032008 | -1.85628838033706 | -3.42694935452096 |
| C | -6.65209847906431 | -0.38015844749221 | -3.15205181814390 |
| H | -7.15837060496701 | 0.38241962015324 | -3.73641534206808 |
| C | -5.41871664520813 | -0.09177064419355 | -2.58397607305366 |
| H | -4.98488903227025 | 0.88769429766896 | -2.73337531868711 |
| C | -2.38430073622631 | -2.98271218550842 | -1.54689915622361 |
| C | -2.38260882394467 | -3.27276709180320 | -2.91556104879900 |
| C | -2.44620528203811 | -4.61748477840376 | -3.28762474121586 |
| H | -2.44691611329642 | -4.87967054708299 | -4.34164672279198 |
| C | -2.49623972571506 | -5.61837917122535 | -2.33151856643897 |
| H | -2.54779670393181 | -6.65913767307785 | -2.63871435269627 |
| C | -2.45637408013042 | -5.30045644208224 | -0.97794074505390 |
| H | -2.46654073946213 | -6.10108431558049 | -0.24670733122574 |
| C | -2.39163407220770 | -3.97572000951330 | -0.54856813126884 |
| C | -2.27158502176462 | -2.20279450888803 | -3.98517529611454 |
| H | -2.28666186404779 | -1.22159207932006 | -3.49766196953328 |

| | | | |
|---|---|---|---|
| C | -0.92690684944155 | -2.32365018383221 | -4.71804254598192 |
| H | -0.09314752792660 | -2.28752876617039 | -4.00856097954202 |
| H | -0.86488151800449 | -3.26861468175484 | -5.26947106928128 |
| H | -0.81428821324020 | -1.50519934975081 | -5.43810888426027 |
| C | -3.44639567466163 | -2.25176501824700 | -4.97141370987903 |
| H | -3.43116861788264 | -3.17609485970078 | -5.55939099151589 |
| H | -4.40851637088604 | -2.19375087888450 | -4.45333232315453 |
| H | -3.38250694325414 | -1.41282050103556 | -5.67329483251924 |
| C | -2.35021815427949 | -3.62443197573665 | 0.93287507827229 |
| H | -1.64337402054255 | -2.79354939307824 | 1.05596843155513 |
| C | -3.71068009875699 | -3.13054426298402 | 1.45294769835506 |
| H | -4.05516130703456 | -2.23257489812200 | 0.93291352631535 |
| H | -4.47542467318317 | -3.90924084707828 | 1.34138564694533 |
| H | -3.62709612253465 | -2.87509310273957 | 2.51438336867911 |
| C | -1.85801208089658 | -4.77749181945489 | 1.80934948908961 |
| H | -2.59410748884243 | -5.58791003956312 | 1.86796542696900 |
| H | -0.91352100638701 | -5.19166001156915 | 1.44262636377644 |
| H | -1.69625686687800 | -4.41080621703007 | 2.82754546707306 |
| C | -3.76445155975564 | 1.64820848983033 | -0.68002387795812 |
| C | -3.52819459728127 | 2.55987056139917 | -1.72237756156749 |
| C | -4.22683129141084 | 3.76444935219870 | -1.68975392831562 |
| H | -4.06414710027046 | 4.50034964458450 | -2.47138009772682 |
| C | -5.13033600882120 | 4.03634477040532 | -0.66982661149327 |
| H | -5.66665720107315 | 4.98096909501911 | -0.66172833960798 |
| C | -5.34235711256980 | 3.11427365300454 | 0.34329150927788 |
| H | -6.04100591003966 | 3.34628008358143 | 1.14097934519887 |
| C | -4.65233123806623 | 1.90038073104102 | 0.37094114754276 |
| C | -2.55227105524250 | 2.26313747179598 | -2.84904741688950 |
| H | -2.34262845688766 | 1.18617153645714 | -2.84731104522273 |
| C | -3.11380529630764 | 2.61804377807141 | -4.23238752599316 |
| H | -3.22787577725773 | 3.69987825382375 | -4.35635839520886 |

| | | | |
|---|---|---|---|
| H | -2.42429739739706 | 2.27210999796201 | -5.00982234807056 |
| H | -4.08847814476678 | 2.15306598840950 | -4.41375166186036 |
| C | -1.22132969574351 | 2.98974780490842 | -2.61647867908922 |
| H | -1.37267909488145 | 4.07494026435293 | -2.60819318984763 |
| H | -0.78156093368893 | 2.71225408467464 | -1.65492920664661 |
| H | -0.50887825704599 | 2.74322453118689 | -3.41222989488019 |
| C | -4.87883650112880 | 0.91373481152773 | 1.49940627700317 |
| H | -4.05832730228368 | 0.18597797502092 | 1.48687734514466 |
| C | -4.84722514852775 | 1.59505086175557 | 2.87275996984041 |
| H | -3.94445451319764 | 2.20059229160333 | 2.99299668633786 |
| H | -5.72323136347582 | 2.23563193356542 | 3.02488471460869 |
| H | -4.84878458281487 | 0.83440698917148 | 3.65979157609047 |
| C | -6.20128970862744 | 0.15586738553435 | 1.30270298571815 |
| H | -6.33045545194922 | -0.58912603150006 | 2.09591430848421 |
| H | -7.04925692184295 | 0.84953087015095 | 1.34647117902319 |
| H | -6.23865325465825 | -0.35813900014149 | 0.33727436038857 |
| I | -1.26194299220090 | 0.02941666887214 | 3.33590840656928 |
| I | -1.35185011943510 | 3.83129680832301 | 1.44564602533509 |
| Ga | -0.71838845450988 | 1.36603116482552 | 1.11740833267855 |
| N | 2.34824911791094 | 1.60807023389506 | 1.30940741022091 |
| N | 2.94521111689455 | -0.40027222370132 | 0.76460848363113 |
| C | 3.40959151106787 | 0.76340360750216 | 1.27904225186589 |
| C | 1.20634159610054 | 0.95703834353981 | 0.83722670610920 |
| C | 1.57996612685355 | -0.32198341577495 | 0.46571506431323 |
| C | 4.76988357821798 | 1.03804656359300 | 1.74248206635922 |
| C | 5.36130492204350 | 2.30098501481259 | 1.57843307346356 |
| H | 4.81774958432793 | 3.10294057810634 | 1.09636240587429 |
| C | 6.65019684726876 | 2.54031997691673 | 2.03532563607448 |
| H | 7.08650629034627 | 3.52485163830120 | 1.89589516891738 |
| C | 7.37750343906831 | 1.53459771022228 | 2.66373755323948 |
| H | 8.38540361862552 | 1.72712430336411 | 3.01922755253761 |

| | | | |
|---|---|---|---|
| C | 6.79836142384755 | 0.28141141686804 | 2.83774605083175 |
| H | 7.34771773448379 | -0.51145558586380 | 3.33650118103290 |
| C | 5.51009492031782 | 0.03325938215214 | 2.38621340214524 |
| H | 5.07436099268291 | -0.94473147445566 | 2.54481051873300 |
| C | 2.41173026692924 | 2.98037515363057 | 1.74630797452770 |
| C | 2.50093865361853 | 3.25363705373857 | 3.11561288590240 |
| C | 2.66359456812047 | 4.58939470051573 | 3.49239649397583 |
| H | 2.74085447023536 | 4.83774932546423 | 4.54664501607634 |
| C | 2.71439149691455 | 5.59794069048331 | 2.54549643731124 |
| H | 2.84226368581551 | 6.63017570746815 | 2.85892631921709 |
| C | 2.56762653182414 | 5.29988061701761 | 1.19546890820741 |
| H | 2.56487370240054 | 6.10773927333752 | 0.47208062424350 |
| C | 2.40331156518364 | 3.98667683236974 | 0.76229375165382 |
| C | 2.39410729222843 | 2.18595336260326 | 4.18738630099524 |
| H | 2.18782047956120 | 1.22337084034913 | 3.70362682795652 |
| C | 1.21306326862449 | 2.48626977748489 | 5.12260684639470 |
| H | 0.28880786356314 | 2.63715950849996 | 4.55868502532108 |
| H | 1.40494835945478 | 3.38263500838128 | 5.72306115305470 |
| H | 1.05570698606273 | 1.64647624678061 | 5.80727949099456 |
| C | 3.69571598093649 | 2.05492975529330 | 4.99250834281497 |
| H | 3.89915693278487 | 2.97456746736064 | 5.55286485606644 |
| H | 4.55687742371153 | 1.85463064641561 | 4.34897642481280 |
| H | 3.60737563865533 | 1.23692964077693 | 5.71610600436658 |
| C | 2.23354745754058 | 3.66699750680035 | -0.71535496208638 |
| H | 1.55954759540035 | 2.80460578703908 | -0.78680862777144 |
| C | 3.56463639580756 | 3.26999250532967 | -1.37467882182245 |
| H | 4.01287184207224 | 2.38743210409402 | -0.90950668878271 |
| H | 4.28794038653347 | 4.09188006454562 | -1.30865456972741 |
| H | 3.40550722765159 | 3.04199169280002 | -2.43454648995478 |
| C | 1.58830983699820 | 4.81244450705950 | -1.49922673952950 |
| H | 2.26530490518691 | 5.66806921211376 | -1.60168613496051 |

| | | | |
|---|---|---|---|
| H | 0.66399653985944 | 5.14852966598670 | -1.01918695628022 |
| H | 1.34629125473062 | 4.47063010791550 | -2.51039031651709 |
| C | 3.70007069235062 | -1.61392002524829 | 0.60386143514837 |
| C | 3.50347652330855 | -2.62978608383164 | 1.55225733369901 |
| C | 4.23688911957450 | -3.80476955486407 | 1.39195103720858 |
| H | 4.10962332332036 | -4.61532825262604 | 2.10362540469415 |
| C | 5.12921468175313 | -3.95163649193537 | 0.33712778795548 |
| H | 5.69575703149485 | -4.87266316265844 | 0.23287645385745 |
| C | 5.29047723171811 | -2.93284054619457 | -0.59081372240101 |
| H | 5.97821727682723 | -3.06698864890902 | -1.42079613032305 |
| C | 4.56824832728170 | -1.74297809136100 | -0.48532121137521 |
| C | 2.52671872017406 | -2.48278049408650 | 2.70577426135469 |
| H | 2.18821881933236 | -1.44162902917918 | 2.75182644412235 |
| C | 3.15757350142294 | -2.82122725064026 | 4.06264305623140 |
| H | 3.43933769807842 | -3.87754268834330 | 4.12877211368576 |
| H | 2.43686524626292 | -2.62016093950405 | 4.86224186526104 |
| H | 4.05230358052367 | -2.21968632504058 | 4.25580811450134 |
| C | 1.28228084319422 | -3.34417835449602 | 2.46062719485668 |
| H | 1.53709644179838 | -4.41051088537198 | 2.45850310802377 |
| H | 0.82678228487789 | -3.10898710395123 | 1.49077537971657 |
| H | 0.53379081742258 | -3.15804468500208 | 3.23716611406773 |
| C | 4.72244974963532 | -0.66086791489633 | -1.53496735292333 |
| H | 4.00013477804045 | 0.13212953069260 | -1.31165939601503 |
| C | 4.38013903606852 | -1.19607069468764 | -2.93229387205151 |
| H | 3.38395192239904 | -1.65069545119336 | -2.93804379661577 |
| H | 5.10544539775958 | -1.94888032232788 | -3.26021076365366 |
| H | 4.39747009647657 | -0.37800445833775 | -3.66103024854036 |
| C | 6.12896971590510 | -0.04630943071558 | -1.50431087991301 |
| H | 6.19807344047413 | 0.77770578856498 | -2.22353197144661 |
| H | 6.88552888406190 | -0.79178010803876 | -1.77455862156231 |
| H | 6.37677838925763 | 0.34156153954313 | -0.51103381718865 |

Optimized Cartesian coordinates of **5′** (UKS triplet) at the r$^2$SCAN-3c level.

--------------------------------------------------------------------------------

| | | | |
|---|---|---|---|
| I | 2.281208763 | -0.713755901 | 3.593556010 |
| Ga | 0.574585490 | -0.397054478 | 1.692171615 |
| N | 0.390588611 | 2.653497013 | 1.601359081 |
| N | -0.905996508 | 2.987066882 | -0.110050947 |
| C | -0.329932118 | 3.621889685 | 0.954516298 |
| C | 0.259207713 | 1.424517696 | 0.967729030 |
| C | -0.552525794 | 1.643583718 | -0.145458137 |
| C | -0.470802263 | 5.014019397 | 1.340555984 |
| C | 0.555269252 | 5.692442758 | 2.033986723 |
| H | 1.480272053 | 5.187450815 | 2.276663104 |
| C | 0.404002240 | 7.018002105 | 2.409175255 |
| H | 1.214647618 | 7.509878178 | 2.938978201 |
| C | -0.762138048 | 7.718165256 | 2.108766079 |
| H | -0.874228913 | 8.756413504 | 2.405398462 |
| C | -1.781267089 | 7.064197385 | 1.420680016 |
| H | -2.701356919 | 7.588531692 | 1.178857792 |
| C | -1.644986513 | 5.737666534 | 1.043258854 |
| H | -2.460382150 | 5.259465111 | 0.518774963 |
| C | 1.133443649 | 2.854466103 | 2.814228380 |
| C | 0.428713877 | 3.011448470 | 4.011596668 |
| C | 1.173642405 | 3.274814775 | 5.163252917 |
| H | 0.661618375 | 3.402325253 | 6.112642163 |
| C | 2.554811834 | 3.368445704 | 5.110536110 |
| H | 3.117782130 | 3.577240571 | 6.015808531 |
| C | 3.229470779 | 3.165813714 | 3.911357211 |
| H | 4.313296951 | 3.201639820 | 3.900572528 |
| C | 2.536687473 | 2.891067958 | 2.734952693 |
| C | -1.079194082 | 2.873545770 | 4.100578308 |
| H | -1.472599875 | 2.703670942 | 3.092121914 |

| | | | |
|---|---|---|---|
| C | -1.452891513 | 1.646763218 | 4.946006525 |
| H | -0.987202280 | 0.739038781 | 4.548338000 |
| H | -1.123155005 | 1.767897035 | 5.983770663 |
| H | -2.539846728 | 1.508352840 | 4.950618374 |
| C | -1.739699235 | 4.146845694 | 4.646513285 |
| H | -1.445994913 | 4.331036019 | 5.685873773 |
| H | -1.464353876 | 5.024752865 | 4.053846381 |
| H | -2.830321661 | 4.043725442 | 4.622774952 |
| C | 3.265341125 | 2.648345206 | 1.421716397 |
| H | 2.706553818 | 1.873248996 | 0.881499360 |
| C | 3.284380575 | 3.905099915 | 0.535839552 |
| H | 2.277787127 | 4.251673477 | 0.283948897 |
| H | 3.808568272 | 4.725462313 | 1.040695637 |
| H | 3.808520982 | 3.693375799 | -0.402731710 |
| C | 4.689266911 | 2.122463702 | 1.616379005 |
| H | 5.357544803 | 2.893246903 | 2.017231039 |
| H | 4.704154429 | 1.258334582 | 2.288035789 |
| H | 5.097344255 | 1.811219608 | 0.649263966 |
| C | -1.798778928 | 3.573918512 | -1.070185131 |
| C | -3.177683118 | 3.393543092 | -0.879046432 |
| C | -4.030550685 | 3.950189122 | -1.830039616 |
| H | -5.104254020 | 3.824871857 | -1.725052270 |
| C | -3.526384264 | 4.662685132 | -2.911097649 |
| H | -4.208425950 | 5.089474400 | -3.640903696 |
| C | -2.157886138 | 4.821545060 | -3.071029093 |
| H | -1.778078769 | 5.370508917 | -3.927627540 |
| C | -1.259246965 | 4.268032259 | -2.157535326 |
| C | -3.740751662 | 2.641102111 | 0.315551466 |
| H | -2.922718851 | 2.458192651 | 1.022206192 |
| C | -4.815760401 | 3.448063503 | 1.057284835 |
| H | -5.706949237 | 3.596553353 | 0.438520335 |

| | | | |
|---|---|---|---|
| H | -5.127680214 | 2.907821706 | 1.957660202 |
| H | -4.447790474 | 4.432205897 | 1.365138746 |
| C | -4.293944609 | 1.272405706 | -0.104825558 |
| H | -5.112990315 | 1.385622997 | -0.824255861 |
| H | -3.521039704 | 0.662540749 | -0.580340064 |
| H | -4.674892198 | 0.734290436 | 0.770514078 |
| C | 0.235754909 | 4.428332493 | -2.354213076 |
| H | 0.744873153 | 3.783321159 | -1.628583388 |
| C | 0.674496558 | 3.967898562 | -3.750684836 |
| H | 0.324080980 | 2.951997970 | -3.958153270 |
| H | 0.285917542 | 4.629469848 | -4.532557110 |
| H | 1.767649711 | 3.980709678 | -3.819985676 |
| C | 0.672663474 | 5.876688359 | -2.088168187 |
| H | 1.760005225 | 5.969415983 | -2.188845969 |
| H | 0.206824839 | 6.559331641 | -2.808204702 |
| H | 0.390073022 | 6.203202650 | -1.082233185 |
| I | -2.281208763 | 0.713755901 | -3.593556010 |
| Ga | -0.574585490 | 0.397054478 | -1.692171615 |
| N | -0.390588611 | -2.653497013 | -1.601359081 |
| N | 0.905996508 | -2.987066882 | 0.110050947 |
| C | 0.329932118 | -3.621889685 | -0.954516298 |
| C | -0.259207713 | -1.424517696 | -0.967729030 |
| C | 0.552525794 | -1.643583718 | 0.145458137 |
| C | 0.470802263 | -5.014019397 | -1.340555984 |
| C | -0.555269252 | -5.692442758 | -2.033986723 |
| H | -1.480272053 | -5.187450815 | -2.276663104 |
| C | -0.404002240 | -7.018002105 | -2.409175255 |
| H | -1.214647618 | -7.509878178 | -2.938978201 |
| C | 0.762138048 | -7.718165256 | -2.108766079 |
| H | 0.874228913 | -8.756413504 | -2.405398462 |
| C | 1.781267089 | -7.064197385 | -1.420680016 |

| | | | |
|---|---|---|---|
| H | 2.701356919 | -7.588531692 | -1.178857792 |
| C | 1.644986513 | -5.737666534 | -1.043258854 |
| H | 2.460382150 | -5.259465111 | -0.518774963 |
| C | -1.133443649 | -2.854466103 | -2.814228380 |
| C | -0.428713877 | -3.011448470 | -4.011596668 |
| C | -1.173642405 | -3.274814775 | -5.163252917 |
| H | -0.661618375 | -3.402325253 | -6.112642163 |
| C | -2.554811834 | -3.368445704 | -5.110536110 |
| H | -3.117782130 | -3.577240571 | -6.015808531 |
| C | -3.229470779 | -3.165813714 | -3.911357211 |
| H | -4.313296951 | -3.201639820 | -3.900572528 |
| C | -2.536687473 | -2.891067958 | -2.734952693 |
| C | 1.079194082 | -2.873545770 | -4.100578308 |
| H | 1.472599875 | -2.703670942 | -3.092121914 |
| C | 1.452891513 | -1.646763218 | -4.946006525 |
| H | 0.987202280 | -0.739038781 | -4.548338000 |
| H | 1.123155005 | -1.767897035 | -5.983770663 |
| H | 2.539846728 | -1.508352840 | -4.950618374 |
| C | 1.739699235 | -4.146845694 | -4.646513285 |
| H | 1.445994913 | -4.331036019 | -5.685873773 |
| H | 1.464353876 | -5.024752865 | -4.053846381 |
| H | 2.830321661 | -4.043725442 | -4.622774952 |
| C | -3.265341125 | -2.648345206 | -1.421716397 |
| H | -2.706553818 | -1.873248996 | -0.881499360 |
| C | -3.284380575 | -3.905099915 | -0.535839552 |
| H | -2.277787127 | -4.251673477 | -0.283948897 |
| H | -3.808568272 | -4.725462313 | -1.040695637 |
| H | -3.808520982 | -3.693375799 | 0.402731710 |
| C | -4.689266911 | -2.122463702 | -1.616379005 |
| H | -5.357544803 | -2.893246903 | -2.017231039 |
| H | -4.704154429 | -1.258334582 | -2.288035789 |

| | | | |
|---|---|---|---|
| H | -5.097344255 | -1.811219608 | -0.649263966 |
| C | 1.798778928 | -3.573918512 | 1.070185131 |
| C | 3.177683118 | -3.393543092 | 0.879046432 |
| C | 4.030550685 | -3.950189122 | 1.830039616 |
| H | 5.104254020 | -3.824871857 | 1.725052270 |
| C | 3.526384264 | -4.662685132 | 2.911097649 |
| H | 4.208425950 | -5.089474400 | 3.640903696 |
| C | 2.157886138 | -4.821545060 | 3.071029093 |
| H | 1.778078769 | -5.370508917 | 3.927627540 |
| C | 1.259246965 | -4.268032259 | 2.157535326 |
| C | 3.740751662 | -2.641102111 | -0.315551466 |
| H | 2.922718851 | -2.458192651 | -1.022206192 |
| C | 4.815760401 | -3.448063503 | -1.057284835 |
| H | 5.706949237 | -3.596553353 | -0.438520335 |
| H | 5.127680214 | -2.907821706 | -1.957660202 |
| H | 4.447790474 | -4.432205897 | -1.365138746 |
| C | 4.293944609 | -1.272405706 | 0.104825558 |
| H | 5.112990315 | -1.385622997 | 0.824255861 |
| H | 3.521039704 | -0.662540749 | 0.580340064 |
| H | 4.674892198 | -0.734290436 | -0.770514078 |
| C | -0.235754909 | -4.428332493 | 2.354213076 |
| H | -0.744873153 | -3.783321159 | 1.628583388 |
| C | -0.674496558 | -3.967898562 | 3.750684836 |
| H | -0.324080980 | -2.951997970 | 3.958153270 |
| H | -0.285917542 | -4.629469848 | 4.532557110 |
| H | -1.767649711 | -3.980709678 | 3.819985676 |
| C | -0.672663474 | -5.876688359 | 2.088168187 |
| H | -1.760005225 | -5.969415983 | 2.188845969 |
| H | -0.206824839 | -6.559331641 | 2.808204702 |
| H | -0.390073022 | -6.203202650 | 1.082233185 |

Optimized Cartesian coordinates of **6** (RKS singlet) at the r$^2$SCAN-3c level.

--------------------------------------------------------------------------------

| | | | |
|---|---|---|---|
| N | 2.464306777 | 1.591524621 | 0.848897050 |
| N | 2.249933166 | 1.677976913 | -1.300164649 |
| C | 3.030818720 | 2.090319502 | -0.272925942 |
| C | 1.294993471 | 0.865919639 | 0.552446704 |
| C | 1.164968771 | 0.907619231 | -0.834163660 |
| C | 4.210839243 | 2.946962908 | -0.356241607 |
| C | 5.325142523 | 2.746281949 | 0.472239947 |
| H | 5.336493121 | 1.927452768 | 1.179576738 |
| C | 6.426564250 | 3.587376319 | 0.389351022 |
| H | 7.278578623 | 3.412542232 | 1.039708560 |
| C | 6.445588368 | 4.639705739 | -0.521020477 |
| H | 7.309689357 | 5.294491628 | -0.583496438 |
| C | 5.346609767 | 4.845833089 | -1.349913675 |
| H | 5.344234369 | 5.666164764 | -2.061667219 |
| C | 4.240302635 | 4.012747119 | -1.267280402 |
| H | 3.385703590 | 4.193465996 | -1.905615339 |
| C | 2.942764412 | 1.810057815 | 2.187221477 |
| C | 3.771286179 | 0.833827183 | 2.763966266 |
| C | 4.176878884 | 1.031965726 | 4.082482330 |
| H | 4.814329008 | 0.298005493 | 4.565444630 |
| C | 3.787363165 | 2.166116004 | 4.785504871 |
| H | 4.115020383 | 2.303545570 | 5.812086570 |
| C | 2.990041708 | 3.125518326 | 4.180251068 |
| H | 2.698904734 | 4.010717009 | 4.738078825 |
| C | 2.542829196 | 2.965514429 | 2.867501178 |
| C | 4.220780684 | -0.387715732 | 1.979702868 |
| H | 4.189712013 | -0.130053754 | 0.912707428 |
| C | 5.657488404 | -0.813593610 | 2.303600404 |
| H | 6.360498927 | 0.023707747 | 2.234316142 |

| | | | |
|---|---|---|---|
| H | 5.979186977 | -1.587433325 | 1.598921695 |
| H | 5.737054622 | -1.238990459 | 3.309608167 |
| C | 3.255766823 | -1.559895722 | 2.192879207 |
| H | 3.202516659 | -1.822121759 | 3.255422891 |
| H | 3.591814429 | -2.441057224 | 1.633773070 |
| H | 2.249727617 | -1.303598492 | 1.848761803 |
| C | 1.678184150 | 4.030490997 | 2.221895159 |
| H | 1.330616737 | 3.647492907 | 1.255064957 |
| C | 0.431772101 | 4.335525859 | 3.062776061 |
| H | -0.120891324 | 3.414633966 | 3.277705034 |
| H | -0.226613759 | 5.022479482 | 2.519524588 |
| H | 0.696191976 | 4.813566140 | 4.012612162 |
| C | 2.496966969 | 5.304970066 | 1.963475144 |
| H | 2.828923443 | 5.749884013 | 2.908589555 |
| H | 1.887213258 | 6.048328880 | 1.437395590 |
| H | 3.385503254 | 5.096236482 | 1.358916043 |
| C | 2.622183794 | 1.802861221 | -2.686392508 |
| C | 1.999966309 | 2.773898155 | -3.484405136 |
| C | 2.405542489 | 2.876310000 | -4.814721486 |
| H | 1.940827086 | 3.616131004 | -5.459935897 |
| C | 3.392419834 | 2.045232701 | -5.327510002 |
| H | 3.698865504 | 2.144200747 | -6.364987068 |
| C | 3.976756146 | 1.079151556 | -4.521685411 |
| H | 4.733490226 | 0.420046355 | -4.937482127 |
| C | 3.597858870 | 0.928831428 | -3.187407473 |
| C | 0.882572812 | 3.654992079 | -2.959318813 |
| H | 0.845895642 | 3.548001238 | -1.868250337 |
| C | 1.073291713 | 5.142085781 | -3.285983136 |
| H | 2.031899169 | 5.529306771 | -2.925505519 |
| H | 0.276248724 | 5.729240492 | -2.817359716 |
| H | 1.023473029 | 5.325923904 | -4.364261607 |

| | | | |
|---|---|---|---|
| C | -0.455954170 | 3.157449801 | -3.520684156 |
| H | -0.501224155 | 3.317117586 | -4.604389929 |
| H | -1.293661594 | 3.689587445 | -3.056912231 |
| H | -0.577499312 | 2.083662731 | -3.342647217 |
| C | 4.222360886 | -0.169474619 | -2.347615972 |
| H | 3.737285308 | -0.166411612 | -1.364970102 |
| C | 5.724394281 | 0.069669735 | -2.139216179 |
| H | 6.139056955 | -0.686448148 | -1.462297923 |
| H | 5.918046751 | 1.058640925 | -1.712360018 |
| H | 6.266428418 | 0.000018375 | -3.089157632 |
| C | 3.962000348 | -1.550047081 | -2.966424764 |
| H | 4.476781026 | -1.659974541 | -3.927398496 |
| H | 2.889380832 | -1.702880355 | -3.128848788 |
| H | 4.331320071 | -2.336015980 | -2.297922205 |
| Ga | -0.121320222 | 0.440674385 | 2.082708648 |
| N | -2.464306777 | -1.591524621 | -0.848897050 |
| N | -2.249933166 | -1.677976913 | 1.300164649 |
| C | -3.030818720 | -2.090319502 | 0.272925942 |
| C | -1.294993471 | -0.865919639 | -0.552446704 |
| C | -1.164968771 | -0.907619231 | 0.834163660 |
| C | -4.210839243 | -2.946962908 | 0.356241607 |
| C | -5.325142523 | -2.746281949 | -0.472239947 |
| H | -5.336493121 | -1.927452768 | -1.179576738 |
| C | -6.426564250 | -3.587376319 | -0.389351022 |
| H | -7.278578623 | -3.412542232 | -1.039708560 |
| C | -6.445588368 | -4.639705739 | 0.521020477 |
| H | -7.309689357 | -5.294491628 | 0.583496438 |
| C | -5.346609767 | -4.845833089 | 1.349913675 |
| H | -5.344234369 | -5.666164764 | 2.061667219 |
| C | -4.240302635 | -4.012747119 | 1.267280402 |
| H | -3.385703590 | -4.193465996 | 1.905615339 |

| | | | |
|---|---|---|---|
| C | -2.942764412 | -1.810057815 | -2.187221477 |
| C | -3.771286179 | -0.833827183 | -2.763966266 |
| C | -4.176878884 | -1.031965726 | -4.082482330 |
| H | -4.814329008 | -0.298005493 | -4.565444630 |
| C | -3.787363165 | -2.166116004 | -4.785504871 |
| H | -4.115020383 | -2.303545570 | -5.812086570 |
| C | -2.990041708 | -3.125518326 | -4.180251068 |
| H | -2.698904734 | -4.010717009 | -4.738078825 |
| C | -2.542829196 | -2.965514429 | -2.867501178 |
| C | -4.220780684 | 0.387715732 | -1.979702868 |
| H | -4.189712013 | 0.130053754 | -0.912707428 |
| C | -5.657488404 | 0.813593610 | -2.303600404 |
| H | -6.360498927 | -0.023707747 | -2.234316142 |
| H | -5.979186977 | 1.587433325 | -1.598921695 |
| H | -5.737054622 | 1.238990459 | -3.309608167 |
| C | -3.255766823 | 1.559895722 | -2.192879207 |
| H | -3.202516659 | 1.822121759 | -3.255422891 |
| H | -3.591814429 | 2.441057224 | -1.633773070 |
| H | -2.249727617 | 1.303598492 | -1.848761803 |
| C | -1.678184150 | -4.030490997 | -2.221895159 |
| H | -1.330616737 | -3.647492907 | -1.255064957 |
| C | -0.431772101 | -4.335525859 | -3.062776061 |
| H | 0.120891324 | -3.414633966 | -3.277705034 |
| H | 0.226613759 | -5.022479482 | -2.519524588 |
| H | -0.696191976 | -4.813566140 | -4.012612162 |
| C | -2.496966969 | -5.304970066 | -1.963475144 |
| H | -2.828923443 | -5.749884013 | -2.908589555 |
| H | -1.887213258 | -6.048328880 | -1.437395590 |
| H | -3.385503254 | -5.096236482 | -1.358916043 |
| C | -2.622183794 | -1.802861221 | 2.686392508 |
| C | -1.999966309 | -2.773898155 | 3.484405136 |

| | | | |
|---|---|---|---|
| C | -2.405542489 | -2.876310000 | 4.814721486 |
| H | -1.940827086 | -3.616131004 | 5.459935897 |
| C | -3.392419834 | -2.045232701 | 5.327510002 |
| H | -3.698865504 | -2.144200747 | 6.364987068 |
| C | -3.976756146 | -1.079151556 | 4.521685411 |
| H | -4.733490226 | -0.420046355 | 4.937482127 |
| C | -3.597858870 | -0.928831428 | 3.187407473 |
| C | -0.882572812 | -3.654992079 | 2.959318813 |
| H | -0.845895642 | -3.548001238 | 1.868250337 |
| C | -1.073291713 | -5.142085781 | 3.285983136 |
| H | -2.031899169 | -5.529306771 | 2.925505519 |
| H | -0.276248724 | -5.729240492 | 2.817359716 |
| H | -1.023473029 | -5.325923904 | 4.364261607 |
| C | 0.455954170 | -3.157449801 | 3.520684156 |
| H | 0.501224155 | -3.317117586 | 4.604389929 |
| H | 1.293661594 | -3.689587445 | 3.056912231 |
| H | 0.577499312 | -2.083662731 | 3.342647217 |
| C | -4.222360886 | 0.169474619 | 2.347615972 |
| H | -3.737285308 | 0.166411612 | 1.364970102 |
| C | -5.724394281 | -0.069669735 | 2.139216179 |
| H | -6.139056955 | 0.686448148 | 1.462297923 |
| H | -5.918046751 | -1.058640925 | 1.712360018 |
| H | -6.266428418 | -0.000018375 | 3.089157632 |
| C | -3.962000348 | 1.550047081 | 2.966424764 |
| H | -4.476781026 | 1.659974541 | 3.927398496 |
| H | -2.889380832 | 1.702880355 | 3.128848788 |
| H | -4.331320071 | 2.336015980 | 2.297922205 |
| Ga | 0.121320222 | -0.440674385 | -2.082708648 |

---------------------------------------------------------------------------

# References


1. G. R. Fulmer, A. J. M. Miller, N. H. Sherden, H. E. Gottlieb, A. Nudelman, B. M. Stoltz, J. E. Bercaw and K. I. Goldberg, *Organometallics*, 2010, **29**, 2176–2179.
2. N. K. T. Ho, B. Neumann, H.-G. Stammler, V. H. Menezes da Silva, D. G. Watanabe, A. A. C. Braga and R. S. Ghadwal, *Dalton Trans.*, 2017, **46**, 12027–12031.
3. M.-C. Brandys, M. C. Jennings and R. J. Puddephatt, *J. Chem. Soc., Dalton Trans.*, 2000, 4601–4606.
4. A. E. Shirk, D. F. Shriver, J. A. Dilts and R. W. Nutt, in *Inorganic Syntheses*, 1977, p. 45–47.
5. O. V. Dolomanov, L. J. Bourhis, R. J. Gildea, J. A. K. Howard and H. Puschmann, *J. Appl. Cryst.*, 2009, **42**, 339–341.
6. G. Sheldrick, *Acta Cryst. A*, 2015, **71**, 3–8.
7. G. Sheldrick, *Acta Cryst. C*, 2015, **71**, 3–8.
8. L. J. Bourhis, O. V. Dolomanov, R. J. Gildea, J. A. K. Howard and H. Puschmann, *Acta Cryst. A*, 2015, **71**, 59–75.
9. F. Kleemiss, O. V. Dolomanov, M. Bodensteiner, N. Peyerimhoff, L. Midgley, L. J. Bourhis, A. Genoni, L. A. Malaspina, D. Jayatilaka, J. L. Spencer, F. White, B. Grundkötter-Stock, S. Steinhauer, D. Lentz, H. Puschmann and S. Grabowsky, *Chem. Sci.*, 2021, **12**, 1675–1692.
10. S. Grimme, A. Hansen, S. Ehlert and J.-M. Mewes, *J. Chem. Phys.*, 2021, **154**, 064103.
11. (a) C. Adamo and V. Barone, *J. Chem. Phys.*, 1999, **110**, 6158–6170; (b) S. Grimme, S. Ehrlich and L. Goerigk, *J. Comput. Chem.*, 2011, **32**, 1456–1465; (c) F. Weigend and R. Ahlrichs, *Phys. Chem. Chem. Phys.*, 2005, **7**, 3297–3305.
12. F. Neese, *WIREs Comp. Mol. Sci.*, 2022, **12**, e1606.
13. F. Neese, F. Wennmohs, A. Hansen and U. Becker, *Chem. Phys.*, 2009, **356**, 98–109.
14. C. A. Bauer, A. Hansen and S. Grimme, *Chem. Eur. J.*, 2017, **23**, 6150–6164.
15. C. R. Landis and F. Weinhold, *Valency and Bonding: A Natural Bond Orbital Donor-Acceptor Perspective*, Cambridge University Press, Cambridge, 2005.
16. E. D. Glendening, J. K. Badenhoop, A. E. Reed, J. E. Carpenter, J. A. Bohmann, C. M. Morales, P. Karafiloglou, C. R. Landis and F. Weinhold, *NBO 7.0*, Theoretical Chemistry Institute, University of Wisconsin, Madison, 2018.
17. M. J. Frisch, G. W. Trucks, H. B. Schlegel, G. E. Scuseria, M. A. Robb, J. R. Cheeseman, G. Scalmani, V. Barone, G. A. Petersson, H. Nakatsuji, X. Li, M. Caricato, A. Marenich, J. Bloino, B. G. Janesko, R. Gomperts, B. Mennucci, H. P. Hratchian, J. V. Ortiz, A. F. Izmaylov, J. L. Sonnenberg, D. Williams-Young, F. Ding, F. Lipparini, F. Egidi, J. Goings, B. Peng, A. Petrone, T. Henderson, D. Ranasinghe, V. G. Zakrzewski, J. Gao, N. Rega, G. Zheng, W. Liang, M. Hada, M. Ehara, K. Toyota, R. Fukuda, J. Hasegawa, M. Ishida, T. Nakajima, Y. Honda, O. Kitao, H. Nakai, T. Vreven, K. Throssell, J. J. A. Montgomery, J. E. Peralta, F. Ogliaro, M. Bearpark, J. J. Heyd, E. Brothers, K. N. Kudin, V. N. Staroverov, T. Keith, R. Kobayashi, J. Normand, K. Raghavachari, A. Rendell, J. C. Burant, S. S. Iyengar, J. Tomasi, M. Cossi, J. M. Millam, M. Klene, C. Adamo, R. Cammi, J. W. Ochterski, R. L. Martin, K. Morokuma, O. Farkas, J. B. Foresman and D. J. Fox, *Gaussian 16 Rev. C.01*, Wallingford, CT, 2016.
